# Regioselective formal hydrocyanation of allenes: synthesis of β,γ-unsaturated nitriles with α-all-carbon quaternary centers

**DOI:** 10.3762/bjoc.21.63

**Published:** 2025-04-17

**Authors:** Seeun Lim, Teresa Kim, Yunmi Lee

**Affiliations:** 1 Department of Chemistry, Kwangwoon University, Seoul 01897, Republic of Korea

**Keywords:** α-quaternary nitrile, Cu catalysis, hydrocyanation, regioselectivity, tosyl cyanide

## Abstract

This study introduces a highly selective hydrocyanation method based on copper-catalyzed hydroalumination of allenes with diisobutylaluminum hydride, followed by the regio- and stereoselective allylation with *p*-toluenesulfonyl cyanide. The proposed methodology is efficient for accessing acyclic β,γ-unsaturated nitriles with α-all-carbon quaternary centers and achieves yields up to 99% and excellent regio- and *E*-selectivity. The reaction proceeds under mild conditions and shows broad applicability to di- and trisubstituted allenes. Its practicality is demonstrated through the gram-scale synthesis and functional group transformations of amines, amides, and lactams, emphasizing its versatility and synthetic significance.

## Introduction

Acyclic nitriles that incorporate α-all-carbon quaternary centers are highly valuable structural motifs typically found in natural products, biologically active compounds, and synthetic pharmaceuticals [1–5]. These compounds are important intermediates in organic syntheses because of the versatility of the cyano group, which can be readily transformed into a wide range of functional groups, including amides, carboxylic acids, amines, aldehydes, ketones, and *N*-heterocycles [6–8]. However, the synthesis of all-carbon quaternary centers that contain functional groups is challenging mainly because of their sterically demanding property [9–11]. In this context, the incorporation of cyano groups at the quaternary carbon centers is promising for the development of versatile acyclic all-carbon quaternary stereocenters with diverse functional groups [12–14]. Consequently, the development of selective and predictable strategies for the introduction of cyano groups into quaternary carbon frameworks has become necessary in organic synthesis.

The transition-metal-catalyzed hydrocyanation of carbon–carbon double bonds is one of the most efficient and atom-economical approaches for synthesizing alkyl nitriles [15–16]. Among the potential substrates, allenes have attracted significant attention because of their unique structural features, which consist of two orthogonal and contiguous C=C bonds. This dual π-system configuration promotes selective functionalization, enabling the synthesis of various complex products through a single transformation [17–19]. Therefore, allenes have become versatile intermediates in numerous transition-metal-catalyzed reactions [20–21]. Despite extensive studies on the catalytic hydrocyanation of alkenes [22], including the industrially relevant DuPont adiponitrile process from 1,3-butadiene using nickel catalysts [23], the hydrocyanation of allenes to produce functionalized β,γ-unsaturated nitriles with quaternary carbon centers has not been investigated extensively [24]. The limited investigation of allene hydrocyanation can be attributed to the significant challenges posed by the two orthogonal π-systems in allenes. These challenges include achieving high regioselectivity and controlling (*E*)/(*Z*)-stereoselectivity, as 1,2-addition processes to allenes can generate up to four possible regioisomeric products.

**Scheme 1:**
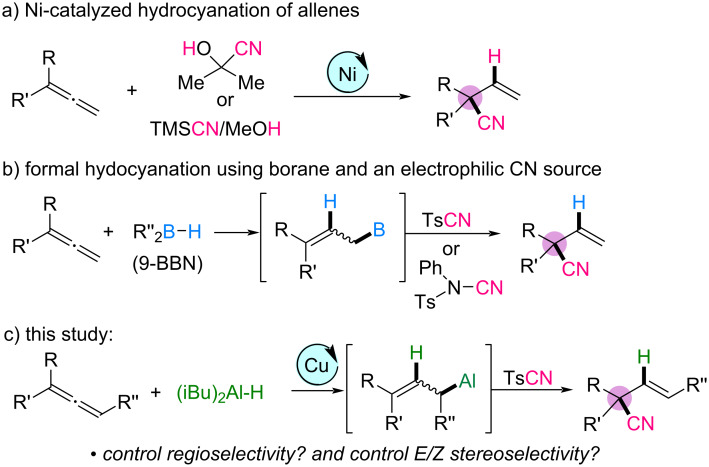
Synthesis of acyclic nitrile-substituted quaternary carbon centers from allenes.

Recent research has addressed some of these challenges. Arai [25–26], Fang [27] and Breit [28] investigated the nickel-catalyzed regio- and enantioselective hydrocyanation of 1,1-disubstituted allenes using acetone cyanohydrin or TMSCN/MeOH as the precursor for the in situ generation of hydrogen cyanide (Scheme 1a). This method achieved high regioselectivity and enantioselectivity, highlighting the potential of allene hydrocyanation for the synthesis of complex nitrile-containing products. In another approach, the Minakata group used electrophilic cyanating reagents, such as *p*-toluenesulfonyl cyanide (TsCN) and *N*-cyano-*N*-phenyl-*p*-toluenesulfonamide [29]. The hydroboration of allenes with 9-BBN (9-borabicyclo[3.3.1]nonane) as the hydride source, followed by regioselective cyanation with allylic boranes, provided nitrile-substituted quaternary carbon centers (Scheme 1b). Although both methodologies achieved high regioselectivity, their substrate scopes were limited, and most studies focused on the hydrocyanation of terminal allenes.

Given the synthetic importance of nitriles that bear all-carbon quaternary centers and the distinctive reactivity of allenes, the development of hydrocyanation methodologies with a broadened substrate scope and improved regio- and stereoselectivity is of significant interest. Inspired by our study on the construction of all-carbon quaternary centers via functionalized allylaluminum reagents obtained from the copper-catalyzed regioselective hydroalumination of allenes using diisobutylaluminum hydride (DIBAL-H), we envisioned that the nucleophilic attack of allylaluminum reagents on electrophilic cyanating reagents could provide a regioselective pathway for the synthesis of alkyl nitriles bearing quaternary carbon centers [30–33]. Herein, we report a mild and efficient method for the regio- and (*E*)-stereoselective formal hydrocyanation of di- and trisubstituted allenes. Using DIBAL-H as the hydride source and TsCN as a readily available and bench-stable cyanating agent in the presence of a copper catalyst, we synthesized new and versatile functionalized acyclic nitriles that include all-carbon quaternary centers with high selectivity (Scheme 1c). Compared to previous methodologies, our approach enables the efficient generation of tertiary nitrile products with a broader substrate scope, highlighting its synthetic utility and potential applicability in complex molecule synthesis.

## Results and Discussion

**Scheme 2:**
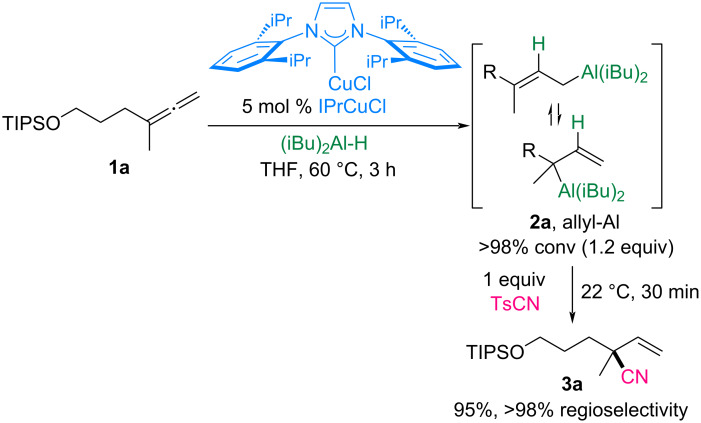
Hydrocyanation of allene **1a** with tosyl cyanide.

We began by optimizing the hydrocyanation of allene **1a** using DIBAL-H as the hydride source and *p*-toluenesulfonyl cyanide as the cyanating reagent (Scheme 2). Under previously established conditions, the hydride addition of DIBAL-H to allene **1a** catalyzed by 5 mol % IPrCuCl as the optimal catalyst selectively generated the allylaluminum intermediate **2a** with >98% conversion [30]. Subsequent addition of one equivalent of TsCN to **2a** in a single vessel at room temperature proceeded regioselectively, achieving complete conversion within 30 min and yielding the desired α-quaternary nitrile **3a** in 95% yield. Moreover, no byproducts, such as regioisomeric nitriles or derivatives from over-addition of allylaluminum, were observed.

**Scheme 3:**
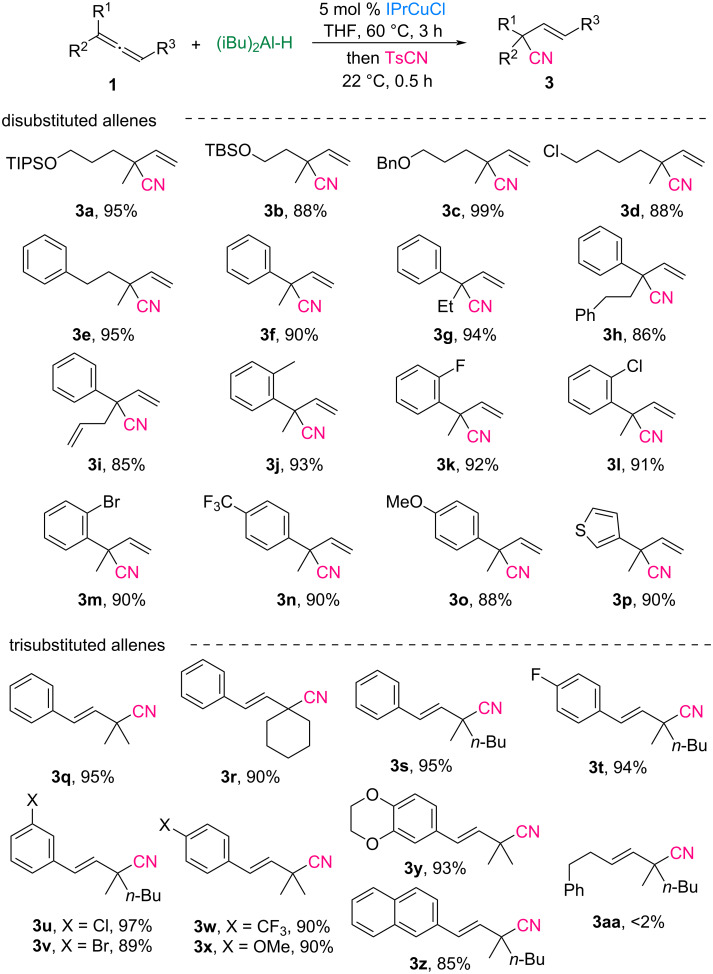
Hydrocyanation with various di- or trisubstituted allenes. Reaction conditions: allene **1** (0.3 mmol), (iBu)_2_Al-H (0.3 mmol), IPrCuCl (5 mol %), TsCN (0.25 mmol), THF (0.2 M), under N_2_. The yields of the isolated products are given.

After having established the optimized reaction conditions, the substrate scope for the formal hydrocyanation with 1,1-disubstituted and 1,1,3-trisubstituted allenes was examined (Scheme 3). All reactions were performed in the presence of 5 mol % IPrCuCl to generate the allylaluminum reagents in situ, followed by cyanation at room temperature for 30 min. This method efficiently constructed α-all-carbon quaternary centers on β,γ-unsaturated nitriles with excellent >98% regioselectivity and >98% (*E*)-selectivity. 1,1-Disubstituted allenes bearing silyl ether- and benzyl ether-tethered propyl groups were successfully converted into the desired nitriles **3a**–**c** in yields ranging from 88% to 99%. Similarly, chloro-substituted allene **1d** exhibited good tolerance under these conditions, affording the corresponding nitrile **3d** in an 88% yield, whereas phenethyl-substituted allene **1e** provided **3e** in a 95% yield. Allenes **1f**–**i** featuring phenyl and alkyl substituents, including methyl, ethyl, phenethyl, and allyl groups, also underwent smooth cyanation, resulting in α-quaternary nitriles **3f**–**i** in yields of 85–94%. Furthermore, aryl-substituted allenes **1j**–**o**, incorporating electron-donating or electron-withdrawing substituents such as methyl, fluoro, chloro, bromo, trifluoromethyl, or methoxy groups on the phenyl ring, were compatible with the reaction, producing nitriles **3j**–**o** in 88–93% yields. In particular, thienyl-substituted allene **1p** was efficiently transformed into the desired nitrile **3p**.

We further demonstrated the versatility of this protocol using 1,1,3-trisubstituted allenes. Trisubstituted allenes **1q**–**s** bearing phenyl and dialkyl groups, including a cyclohexyl moiety, underwent selective cyanation to deliver the (*E*)-isomers of the corresponding nitriles **3q**–**s** in yields of 90–95%. In addition, aryl- and dialkyl-substituted allenes **1t**–**x** containing substituents, such as fluoro, chloro, bromo, trifluoromethyl, or methoxy groups on the phenyl ring, were smoothly converted into β,γ-unsaturated nitriles **3t**–**x** with high efficiency. Particularly, allenes **1y** and **1z** containing benzodioxane or naphthalene moieties were well-tolerated under these reaction conditions, affording nitriles **3y** and **3z** in 85% and 93% yield, respectively. Unfortunately, the 1,1,3-trialkyl-substituted allene **1aa** was not suitable for Cu-catalyzed hydroalumination under the established conditions, resulting in less than 2% conversion to allylaluminum reagents.

**Scheme 4:**
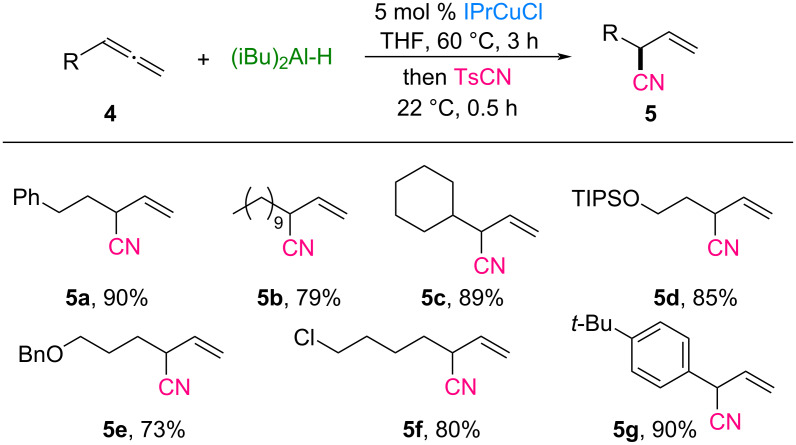
Hydrocyanation with various monosubstituted allenes. Reaction conditions: allene **4** (0.3 mmol), (iBu)_2_Al-H (0.3 mmol), IPrCuCl (5 mol %), TsCN (0.25 mmol), THF (0.2 M), under N_2_. The yields of the isolated products are given.

In a previous study on the electrophilic cyanation of allylic boranes conducted by the Minakata group (Scheme 1b), only two examples of β,γ-unsaturated nitrile products bearing α-tertiary carbon centers were established, and the yields were moderate [29]. To broaden the applicability of the system, we extended it to the synthesis of nitriles containing both quaternary and tertiary carbon centers. The scope of monosubstituted allenes is illustrated in Scheme 4. Allenes **4a**–**c** substituted with alkyl groups, including phenethyl, decyl, and cyclohexyl groups, smoothly underwent hydrocyanation, yielding the corresponding nitriles **5a**–**c** in 79–90% yield with excellent regioselectivity (>98%). Functional groups such as silyl ether, benzyl ether, and chloro moieties on allenes **4d**–**f** were well tolerated under the reaction conditions, producing nitrile-substituted tertiary carbon products **5d**–**f** in yields ranging from 73% to 85%. Moreover, aryl-substituted allene **4g** was efficiently converted to the desired nitrile **5g** in high yield.

**Scheme 5:**
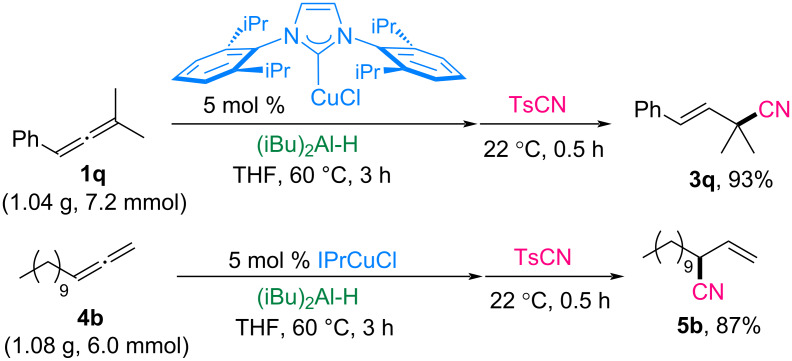
Gram scale reaction.

Gram-scale reactions were conducted using allenes to demonstrate the practical applicability of this hydrocyanation method (Scheme 5). Allene **1q** (1.04 g, 7.2 mmol) and allene **4b** (1.08 g, 6.0 mmol) were effectively transformed into nitrile products **3q** and **5b**, achieving yields of 93% and 87%, respectively. When the catalyst loading was reduced to 3 mol % for the reaction of allene **4b**, the hydroalumination did not reach full conversion even with an extended reaction time (6 h vs 3 h). As a result, the incomplete hydroalumination led to a side reaction between the remaining DIBAL-H and TsCN, ultimately yielding the cyanation product **5b** in only 54%.

**Scheme 6:**
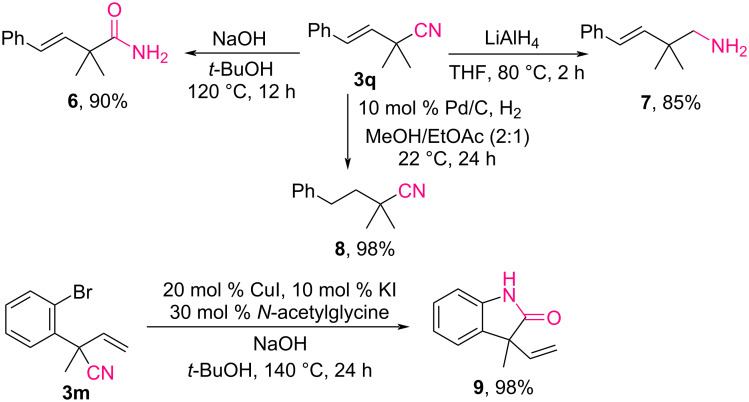
Synthetic applications.

The synthetic potential of the obtained β,γ-unsaturated nitriles featuring α-quaternary carbon centers was further illustrated using a series of transformations (Scheme 6). Nitrile **3q** was hydrolyzed to amide **6** in a 90% yield under basic conditions using sodium hydroxide and *tert*-butanol. The reduction of nitrile **3q** with lithium aluminum hydride generated amine **7** in an 85% yield, whereas the selective hydrogenation of the alkene moiety of **3q** using a Pd/C catalyst in a H_2_ gas environment smoothly produced product **8** in a 98% yield. *Ortho*-bromoaryl-substituted nitrile **3m** also underwent tandem amidation and copper-catalyzed cyclization, efficiently producing lactam **9** in a 98% yield.

**Scheme 7:**
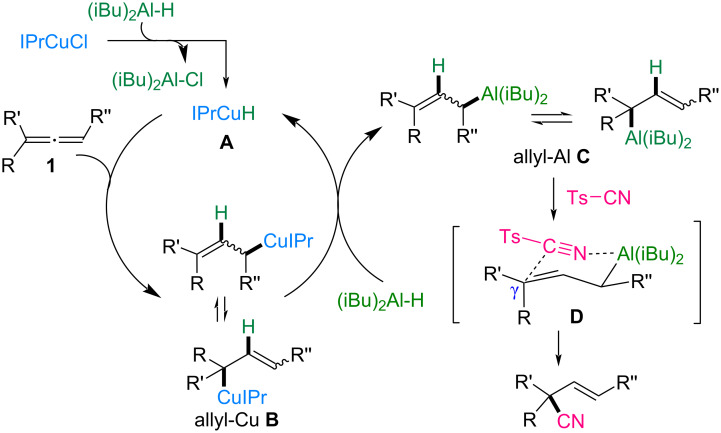
Proposed mechanism.

Scheme 7 illustrates a plausible reaction mechanism based on previous studies [34]. The process begins with the formation of NHC–copper hydride complex **A** through the reaction of IPrCuCl with DIBAL-H [35]. Copper hydride species **A** reacts regioselectively with allene **1** to form the allylcopper intermediate **B**. Subsequent transmetalation between allyl-Cu **B** and DIBAL-H generates allylaluminum species **C** and regenerates IPrCuH (**A**). The final step involves the regioselective nucleophilic attack of allylaluminum **C** on tosyl cyanide, which proceeds at the γ-position via six-membered ring transition state **D**, leading to the formation of the desired nitrile product. Transition state **D** is responsible for the *E*-selectivity observed in trisubstituted allenes, as it minimizes the allylic strain between the R and R'' groups.

## Conclusion

In this study, we developed a highly regio- and (*E*)-selective formal hydrocyanation protocol for allenes using a copper-catalyzed hydroalumination/cyanation sequence with DIBAL-H and tosyl cyanide. This approach offers mild reaction conditions, broad functional group compatibility, and high efficiency, enabling the synthesis of new and versatile functionalized β,γ-unsaturated nitriles containing α-all-carbon quaternary centers with exceptional selectivity. The practicality of this approach was validated through gram-scale synthesis and the successful transformation of nitrile products into amines, amides, and lactams. Further studies are underway to broaden the scope and application of the proposed method.

## Supporting Information

File 1 General information, experimental procedures, characterization data and copies of spectra.

## Data Availability

All data that supports the findings of this study is available in the published article and/or the supporting information of this article.
