# Supplementary material for: Regioselective formal hydrocyanation of allenes: synthesis of β,γ-unsaturated nitriles with α-all-carbon quaternary centers
Source: Beilstein J Org Chem. 2025 Apr 17;21:800–6. doi: 10.3762/bjoc.21.63 (PMC12018896; doi:10.3762/bjoc.21.63)
Supplement: File 1 — General information, experimental procedures, characterization data and copies of spectra. [file Beilstein_J_Org_Chem-21-800-s001.pdf]

BEILSTEIN JOURNAL OF ORGANIC CHEMISTRY

Supporting Information

for

Regioselective formal hydrocyanation of allenes: synthesis of β,γ-unsaturated nitriles with α-all-carbon quaternary centers

Seeun Lim, Teresa Kim and Yunmi Lee

Beilstein J. Org. Chem. **2025,** *21,* 800–806. doi:10.3762/bjoc.21.63

General information, experimental procedures, characterization data and copies of spectra

License and Terms: This is a supporting information file under the terms of the Creative Commons Attribution License (https://creativecommons.org/licenses/by/4.0). Please note that the reuse, redistribution and reproduction in particular requires that the author(s) and source are credited and that individual graphics may be subject to special legal provisions.
The license is subject to the *Beilstein Journal of Organic Chemistry* terms and conditions: (https://www.beilstein-journals.org/bjoc/terms)

Table of contents

1. General information S2

2. Preparation of substrates S2

3. General procedure for the synthesis of β,γ-unsaturated nitriles S3

4. Characterization data for all products S3

5. Procedure for gram-scale reaction S13

6. Synthetic applications S14

7. Copies of ^{1}H and ^{13}C NMR spectra for all products S17

1. General information

^{1}H NMR spectra were recorded a JEOL JNM-AL400 (400 MHz) spectrometer. Chemical shifts are reported in ppm from tetramethylsilane, with the solvent resonance as the internal standard ($CDCl_3$: δ 7.27 ppm). Data are reported as follows: chemical shift, multiplicity (s = singlet, d = doublet, t = triplet, q = quartet, quint = quintet, m = multiplet), coupling constants (Hz), and integration. ^{13}C NMR spectra were recorded on a JEOL JNM-AL400 (100 MHz) spectrometer with complete proton decoupling. Chemical shifts are reported in ppm from tetramethylsilane with the solvent resonance as the internal standard ($CDCl_3$: δ 77.00 ppm). High-resolution mass spectra (HRMS) were performed at the Korea Basic Science Institute for technical assistance using an electrospray ionization (ESI) time-of-flight mass spectrometer. Partially, HRMS data were acquired on a Supercritical Fluid Chromatography combined with Xevo G2-Xs QTQF Mass Spectrometer (Waters, Milford, MA, USA) at the Chiral Material Core Facility Center of Sungkyunkwan University.

Unless otherwise noted, all reactions were carried out with distilled solvents under an atmosphere of dry N_2 in oven-dried (130 °C) glassware. Tetrahydrofuran was purified by distillation from Na immediately prior to use. Diisobutylaluminum hydride (DIBAL-H) was purchased from Sigma-Aldrich Corporation and used as received. *p*-Toluenesulfonyl cyanide was purchased from Sigma-Aldrich Corporation and used as received. All work-up and purification procedures were carried out with reagent grade solvents in air. The NHC-CuCl complex was synthesized according to previously reported experimental procedures.[1]

2. Preparation of substrates

- 1,1-Disubsitutited allenes **1a–p** were prepared according to reported experimental procedures.[2]
- 1,1,3-Trisubstituted allenes **1q–z** were prepared according to reported experimental procedures.[3]
- Monosubstituted allenes **4a–g** were prepared according to reported experimental procedures.[4]

---

(1) Yoo, W.-J.; Nguyen, T. V. Q.; Kobayashi, S. *Angew. Chem., Int. Ed.* **2014**, *53*, 10213–10217.

(2) (a) Lee, S.; Lee, S.; Lee, Y. *Org. Lett.* **2020**, *22*, 5806−5810. (b) Boreux, A.; Indukuri, K.; Gagosz, F.; Riant, O. *ACS Catal.* **2017**, *7*, 8200−8204. (c) Gobé, V.; Guinchard, X. *Chem. Eur. J.*, **2015**, *21*, 8511–8520. (d) Mentink, G.; Van Maarseveen, J. H.; Hiemstra, H. *Org. Lett.* **2002**, *4*, 3497–3500.

(3) (a) Lee, K.; Cho, S.; Lim, S.; Lee, Y. *Org. Chem. Front.* **2024**, *11*,1366–1371. (b) Vial, X. D.; Mascarenas, J. L.; Gulias, M. I. *Org. Lett.* **2021**, *23*, 5323−5328. (c) Eshon, J.; Landis, C. R.; Schomaker, J. M. *J. Org. Chem.* **2017**, *82*, 9270−9278. (d) Ting, C.-M.; Hsu. Y.-L.; Liu, R.-S. *Chem. Commun.* **2012**, 48, 6577−6579.

(4) (a) Rej, S.; Klare, H. F. T.; Oestreich, M. *Org. Lett.* **2022**, *24,* 1346−1350. (b) Kim, Y.; Lee, H.; Park, S.; Lee, Y. *Org. Lett.* **2018**, *20*, 5478−5481. (c) Kuang, J.; Ma, S. *J. Org. Chem.* **2009**, *74*, 1763–1765.

3. General procedure for the synthesis of β,γ-unsaturated nitriles

Under an inert atmosphere within a glove box, IPrCuCl (7.32 mg, 1.50 × 10^{-2} mmol) was added to an 8 mL vial equipped with a stirring bar. The vial was then sealed with a phenolic open-top cap (gray PTFE/silicone) and removed from the glove box. The vial was purged with nitrogen (N_2) gas for 5 minutes. Subsequently, tetrahydrofuran (THF, 1.0 mL) and diisobutylaluminum hydride (DIBAL-H, 54.0 μL, 0.300 mmol) were introduced into the vial. The mixture was allowed to premix for 10 minutes before adding a THF solution (0.5 mL) of triisopropyl((4-methylhexa-4,5-dien-1-yl)oxy)silane (**1a**, 80.6 mg, 0.300 mmol). The reaction mixture was stirred at 60 °C on a preheated heating block for 3 hours. Following this, *p*-toluenesulfonyl cyanide (45.3 mg, 0.250 mmol) was added to the solution via syringe, and the mixture was stirred at ambient temperature for an additional 30 minutes. The reaction was quenched by adding 1 N aqueous HCl (1 mL), and the mixture was subsequently washed with ethyl acetate (1 mL × 3). The organic layers were combined and dried over $MgSO_4$. After filtration, the volatiles were removed under reduced pressure. The crude product was purified by silica gel column chromatography (EtOAc/hexanes 1:20) to yield the desired product **3a** as a colorless oil (70.2 mg, 0.237 mmol, 95% yield).

4. Characterization data for all products

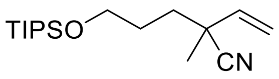

2-Methyl-5-((triisopropylsilyl)oxy)-2-vinylpentanenitrile (3a). Yield: 95% (70.2 mg, 0.237 mmol), colorless oil, silica gel column chromatography (EtOAc:hexanes = 1:20). **^{1}H NMR** ($CDCl_3$, 400 MHz): δ 5.60 (dd, *J* = 17.0, 10.1 Hz, 1H), 5.46 (d, *J* = 17.0 Hz, 1H), 5.24 (d, *J* = 10.1 Hz, 1H), 3.73 (t, *J* = 5.5 Hz, 2H), 1.78-1.64 (m, 4H), 1.45 (s, 3H), 1.11-1.03 (m, 21H); **^{13}C{^{1}H} NMR** ($CDCl_3$, 100 MHz): δ 138.2, 122.3, 115.7, 62.6, 40.4, 36.5, 28.7, 26.0, 18.0, 11.9; **HRMS** (ESI) *m/z*: $[M+H]^+$ Calcd for $C_{17}H_{34}NOSi$ 296.2410, Found 296.2409.

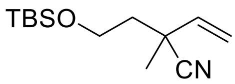

2-(2-((*tert*-Butyldimethylsilyl)oxy)ethyl)-2-methylbut-3-enenitrile (3b). Yield: 88% (52.7 mg, 0.220 mmol), colorless oil, silica gel column chromatography (EtOAc:hexanes = 1:20). This compound has been previously reported, and the spectral data match the described data.[5] **^{1}H NMR** ($CDCl_3$, 400 MHz): δ 5.66 (dd, *J* = 17.0, 10.1 Hz, 1H), 5.46 (d, *J* = 17.0 Hz, 1H), 5.22 (d, *J* = 10.5

(5) Kiyokawa, K.; Hata, S.; Kainuma, S.; Minakata, S. *Chem. Commun.* **2019**, *55*, 458–461.

Hz, 1H), 3.80-3.73 (m, 2H), 1.92-1.89 (m, 1H), 1.84-1.82 (m, 1H), 1.47 (s, 3H), 0.90 (s, 9H), 0.07 (s, 3H), 0.06 (s, 3H)); **^{13}C{^{1}H} NMR** ($CDCl_3$, 100 MHz): δ 138.1, 121.6, 115.4, 59.6, 42.1, 38.7, 26.3, 25.8, 18.2, -5.44, -5.46.

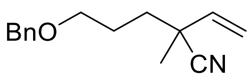

5-(Benzyloxy)-2-methyl-2-vinylpentanenitrile (3c). Yield: 99% (56.8 mg, 0.248mmol), colorless oil, silica gel column chromatography (EtOAc:hexanes = 1:20). **^{1}H NMR** ($CDCl_3$, 400 MHz): δ 7.38-7.28 (m. 5H), 5.60 (dd, J = 17.0, 10.0 Hz, 1H), 5.47 (d, J = 17.0 Hz, 1H), 5.24 (d, J = 10.0 Hz, 1H), 4.50 (s, 2H), 3.51 (t, J = 6.0 Hz, 2H), 1.81-1.67 (m, 4H), 1.44 (s, 3H); **^{13}C{^{1}H} NMR** ($CDCl_3$, 100 MHz): δ 138.3, 138.0, 128.4, 127.6, 122.2, 115.9, 72.9, 69.5, 40.2, 36.8, 25.9, 25.7; **HRMS** (ESI) *m/z*: $[M+Na]^+$ Calcd for $C_{15}H_{19}NNaO$ 252.1364, Found 252.1360.

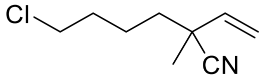

6-Chloro-2-methyl-2-vinylhexanenitrile (3d). Yield: 88% (37.8 mg, 0.220 mmol), colorless oil, silica gel column chromatography (EtOAc:hexanes = 1:20). **^{1}H NMR** ($CDCl_3$, 400 MHz): δ 5.60 (dd, J = 17.0, 10.0 Hz, 1H), 5.47 (d, J = 17.0 Hz, 1H), 5.25 (d, J = 10.0 Hz, 1H), 3.54 (t, J = 6.4 Hz, 2H), 1.81-1.67 (m, 2H), 1.70-1.56 (m, 2H), 1.44 (s, 3H), 1.29-1.19 (m, 2H); **^{13}C{^{1}H} NMR** ($CDCl_3$, 100 MHz): δ 137.8, 122.1, 116.0, 44.4, 29.2, 32.2, 25.9, 22.7, 21.5; **HRMS** (ESI) *m/z*: $[M+Na]^+$ Calcd for $C_9H_{14}ClNNa$ 194.0712, Found 194.0706.

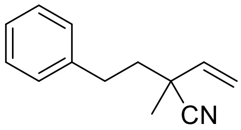

2-Methyl-2-phenethylbut-3-enenitrile (3e). Yield: 95% (43.9 mg, 0.237 mmol), colorless oil, silica gel column chromatography (EtOAc:hexanes = 1:20). This compound has been previously reported, and the spectral data match the described data.[5] **^{1}H NMR** ($CDCl_3$, 400 MHz): δ 7.30 (t, J = 7.8 Hz, 2H), 7.23-7.18 (m, 3H), 5.66 (dd, J = 16.9, 10.0 Hz, 1H), 5.54 (d, J = 17.0 Hz, 1H), 5.30 (d, J = 10.0 Hz, 1H), 2.81-2.68 (m, 2H), 2.02-1.94 (m, 1H), 1.88-1.80 (m, 1H), 1.50 (s, 3H); **^{13}C{^{1}H} NMR** ($CDCl_3$, 100 MHz): δ 141.0, 138.2, 128.9, 128.6, 126.6, 122.3, 116.5, 42.2, 41.0, 32.0, 26.3.

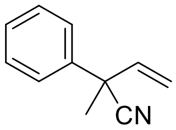

2-Methyl-2-phenylbut-3-enenitrile (3f). Yield: 90% (35.4 mg, 0.225 mmol), colorless oil, silica gel column chromatography (EtOAc:hexanes = 1:20). This compound has been previously reported, and the spectral data match the described data.[5] **^{1}H NMR** ($CDCl_3$, 400 MHz): δ 7.46 (d, J = 7.8 Hz, 2H),

7.40 (t, J = 7.8 Hz, 2H), 7.33 (t, J = 7.4 Hz, 1H), 5.97 (dd, J = 17.2, 10.3 Hz, 1H), 5.54 (d, J = 17.0 Hz, 1H), 5.33 (d, J = 10.1 Hz, 1H), 1.83 (s, 3H); **^{13}C{^{1}H} NMR** ($CDCl_3$, 100 MHz): δ 139.5, 138.3, 129.0, 128.1, 125.9, 121.8, 115.7, 44.5, 26.7.

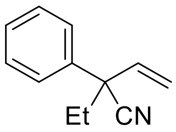

2-Ethyl-2-phenylbut-3-enenitrile (3g). Yield: 94% (40.2 mg, 0.235 mmol), colorless oil, silica gel column chromatography (EtOAc:hexanes = 1:20). This compound has been previously reported, and the spectral data match the described data.[5] **^{1}H NMR** ($CDCl_3$, 400 MHz): δ 7.45 (d, J = 7.3 Hz, 2H), 7.40 (t, J = 7.4 Hz, 2H), 7.33 (t, J = 7.4 Hz, 1H), 5.93 (dd, J = 17.0, 10.1 Hz, 1H), 5.55 (d, J = 17.0 Hz, 1H), 5.34 (d, J = 10.1 Hz, 1H), 2.15-2.01 (m, 2H), 1.04 (t, J = 7.4 Hz, 3H); **^{13}C{^{1}H} NMR** ($CDCl_3$, 100 MHz): δ 138.5, 137.3, 129.0, 127.9, 126.1, 120.6, 116.4, 51.1, 32.9, 9.6.

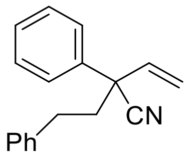

2-Phenethyl-2-phenylbut-3-enenitrile (3h). Yield: 86% (53.2 mg, 0.215 mmol), colorless oil, silica gel column chromatography (EtOAc:hexanes = 1:20). **^{1}H NMR** ($CDCl_3$, 400 MHz): δ 7.50 (d, J = 7.3 Hz, 2H), 7.43 (t, J = 7.4 Hz, 2H), 7.36 (t, J = 7.4 Hz, 1H), 7.30 (t, J = 7.4 Hz, 2H), 7.23-7.17 (m, 3H), 6.00 (dd, J = 17.2, 10.3 Hz, 1H), 5.62 (d, J = 17.0 Hz, 1H), 5.39 (d, J = 10.1 Hz, 1H), 2.82 (td, J = 13.2, 5.0 Hz, 1H), 2.66 (td, J = 13.0, 5.3 Hz, 1H), 2.41-2.26 (m, 2H); **^{13}C{^{1}H} NMR** ($CDCl_3$, 100 MHz): δ 140.46, 138.3, 137.3, 129.1, 128.5, 128.3, 128.1, 126.3, 126.0, 120.4, 116.7, 50.3, 41.7, 31.7 ; **HRMS** (ESI) *m/z*: $[M+H]^+$ Calcd for $C_{18}H_{18}N$ 248.1439, Found 248.1441.

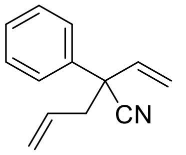

2-Phenyl-2-vinylpent-4-enenitrile (3i). Yield: 85% (38.9 mg, 0.213 mmol), colorless oil, silica gel column chromatography (EtOAc:hexanes = 1:20). **^{1}H NMR** ($CDCl_3$, 400 MHz): δ 7.46 (d, J = 7.3 Hz, 2H), 7.40 (t, J = 7.4 Hz, 2H), 7.33 (t, J = 7.4 Hz, 1H), 5.98 (dd, J = 17.0, 10.1 Hz, 1H), 5.77-5.66 (m, 1H), 5.54 (d, J = 17.0 Hz, 1H), 5.36 (d, J = 10.1 Hz, 1H), 5.20 (d, J = 16.1 Hz, 1H), 5.19 (d, J = 11.5 Hz, 1H), 2.86-2.74 (m, 2H); **^{13}C{^{1}H} NMR** ($CDCl_3$, 100 MHz): δ 138.5, 136.8, 131.4, 129.0, 128.1, 126.2, 120.3, 116.9, 50.1, 44.1; **HRMS** (ESI) *m/z*: $[M+H]^+$ Calcd for $C_{13}H_{14}N$ 184.1126, Found 184.1121.

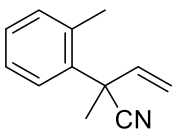

2-Methyl-2-(*o*-tolyl)but-3-enenitrile (3j). Yield: 93% (39.8 mg, 0.232 mmol), colorless oil, silica gel column chromatography (EtOAc:hexanes = 1:20). **^{1}H NMR** ($CDCl_3$, 400 MHz): δ 7.35-7.33 (m, 1H), 7.26-7.19 (m, 3H), 5.83 (dd, J = 17.2, 10.3 Hz, 1H), 5.46 (d, J = 17.0 Hz, 1H), 5.32 (d, J = 10.1 Hz, 1H), 2.50 (s, 3H), 1.88 (s, 3H); **^{13}C{^{1}H} NMR** ($CDCl_3$, 100 MHz): δ 138.1, 137.4, 135.7, 132.5, 128.4, 126.4, 125.5, 121.6, 115.9, 42.8, 26.9, 21.0; **HRMS** (ESI) *m/z*: $[M+Na]^+$ Calcd for $C_{12}H_{13}NNa$ 194.0946, Found 194.0940.

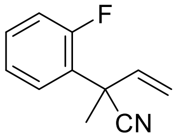

2-(2-Fluorophenyl)-2-methylbut-3-enenitrile (3k). Yield: 92% (40.5 0.231 mmol), colorless oil, silica gel column chromatography (EtOAc:hexanes = 1:20). **^{1}H NMR** ($CDCl_3$, 400 MHz): δ 7.50 (td, J = 7.8, 1.3 Hz, 1H), 7.38-7.32 (m, 1H), 7.18 (t, J = 7.4 Hz, 1H), 7.10 (dd, J = 11.4, 8.2 Hz, 1H), 6.80 (dd, J = 17.2, 10.3 Hz, 1H), 5.56 (d, J = 17.0 Hz, 1H), 5.35 (d, J = 10.1 Hz, 1H), 1.89 (s, 3H); **^{13}C{^{1}H} NMR** ($CDCl_3$, 100 MHz): δ 160.3 (d, $J_{C\text{-}F}$ = 250.5 Hz), 136.4, 130.2 (d, $J_{C\text{-}F}$ = 8.6 Hz), 127.5 (d, $J_{C\text{-}F}$ = 2.8 Hz), 126.3 (d, $J_{C\text{-}F}$ = 10.6 Hz), 124.5 (d, $J_{C\text{-}F}$ = 3.8 Hz), 120.8, 116.7 (d, $J_{C\text{-}F}$ = 22.1 Hz), 116.4, 42.4, 25.7; ^{19}F NMR ($CDCl_3$, 376 MHz): δ -110.38; **HRMS** (ESI) *m/z*: $[M+H]^+$ Calcd for $C_{11}H_{11}FN$ 176.0876, Found 176.0874.

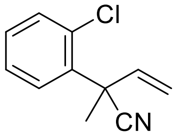

2-(2-Chlorophenyl)-2-methylbut-3-enenitrile (3l). Yield: 91% (43.5 mg, 0.227 mmol), colorless oil, silica gel column chromatography (EtOAc:hexanes = 1:20). **^{1}H NMR** ($CDCl_3$, 400 MHz): δ 7.7.54-7.51 (m, 1H), 7.46-7.43 (m, 1H), 7.33-7.30 (m, 2H), 6.06 (dd, J = 17.2, 10.3 Hz, 1H), 5.53 (d, J = 17.0 Hz, 1H), 5.41 (d, J = 10.1 Hz, 1H), 1.97 (s, 3H); **^{13}C{^{1}H} NMR** ($CDCl_3$, 100 MHz): δ 136.9, 135.6, 130.2, 129.8, 128.2, 127.8, 127.0, 121.2, 118.0, 44.5, 26.2; **HRMS** (ESI) *m/z*: $[M+H]^+$ Calcd for $C_{11}H_{11}ClN$ 192.0580, Found 192.0578.

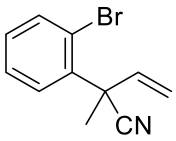

2-(2-Bromophenyl)-2-methylbut-3-enenitrile (3m). Yield: 90% (53.1 mg, 0.225 mmol), yellowish oil, silica gel column chromatography (EtOAc:hexanes = 1:20). **^{1}H NMR** ($CDCl_3$, 400 MHz): δ 7.67

(dd, J = 7.8, 1.0 Hz, 1H), 7.53 (dd, J = 8.3, 1.4 Hz, 1H), 7.36 (td, J = 8.3, 1.4 Hz, 1H), 7.22 (td, J = 7.4, 1.4 Hz, 1H), 6.07 (dd, J = 17.2, 10.3 Hz, 1H), 5.52 (d, J = 17.0 Hz, 1H), 5.43 (d, J = 10.1 Hz, 1H), 1.99 (s, 3H); **^{13}C{^{1}H} NMR** ($CDCl_3$, 100 MHz): δ 136.9, 135.6, 130.2, 129.8, 128.2, 127.8, 127.0, 120.9, 117.4, 44.7, 26.2; **HRMS** (ESI) *m/z*: $[M+Na]^+$ Calcd for $C_{11}H_{10}BrNNa$ 257.9894, Found 257.9889.

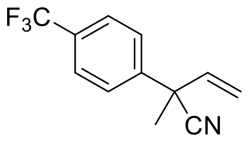

2-Methyl-2-(4-(trifluoromethyl)phenyl)but-3-enenitrile (3n). Yield: 90% (50.9 mg, 0.226 mmol), colorless oil, silica gel column chromatography (EtOAc:hexanes = 1:20). **^{1}H NMR** ($CDCl_3$, 400 MHz): δ 7.67 (d, J = 8.2 Hz, 2H), 7.59 (d, J = 8.3 Hz, 2H), 5.96 (dd, J = 17.2, 10.3 Hz, 1H), 5.57 (d, J = 17.0 Hz, 1H), 5.40 (d, J = 10.1 Hz, 1H), 1.85 (s, 3H); **^{13}C{^{1}H} NMR** ($CDCl_3$, 100 MHz): δ 143.4, 137.4, 130.2 (q, $J_{C\text{-}F}$ = 32.7 Hz), 126.4 (q, $J_{C\text{-}F}$ = 272.6 Hz), 126.5, 125.0 (q, $J_{C\text{-}F}$ = 3.8 Hz), 121.1, 116.7, 44.4, 26.6; **^{19}F NMR** ($CDCl_3$, 376 MHz): δ -110.73; **HRMS** (ESI) *m/z*: $[M+H]^+$ Calcd for $C_{12}H_{11}F_3N$ 226.0844, Found 226.0844.

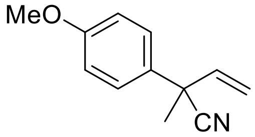

2-(4-Methoxyphenyl)-2-methylbut-3-enenitrile (3o). Yield: 88% (41.4 mg, 0.221 mmol), white solid, silica gel column chromatography (EtOAc:hexanes = 1:10). This compound has been previously reported, and the spectral data match the described data.[5] **^{1}H NMR** ($CDCl_3$, 400 MHz): δ 7.34 (d, J = 8.7 Hz, 2H), 6.90 (d, J = 8.7 Hz, 2H), 5.90 (dd, J = 17.2, 10.3 Hz, 1H), 5.50 (d, J = 17.0 Hz, 1H), 5.28 (d, J = 10.1 Hz, 1H), 3.80 (s, 3H), 1.78 (s, 3H); **^{13}C{^{1}H} NMR** ($CDCl_3$, 100 MHz): δ 159.0, 138.6, 130.9, 127.1, 122.0, 115.3, 114.2, 55.3, 43.7, 26.6.

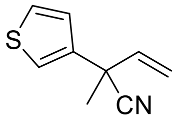

2-Methyl-2-(thiophen-3-yl)but-3-enenitrile (3p). Yield: 90% (36.9 mg, 0.226 mmol), yellowish oil, silica gel column chromatography (EtOAc:hexanes = 1:10). **^{1}H NMR** ($CDCl_3$, 400 MHz): δ 7.36-7.34 (m, 1H), 7.25-7.24 (m, 1H), 7.06 (dd, J = 5.0, 0.9 Hz, 1H), 5.91 (dd, J = 17.2, 10.3 Hz, 1H), 5.51 (d, J = 17.0 Hz, 1H), 5.29 (d, J = 10.1 Hz, 1H), 1.80 (s, 3H); **^{13}C{^{1}H} NMR** ($CDCl_3$, 100 MHz): δ 140.3, 137.8, 127.1, 125.7, 121.5, 121.5, 115.6, 41.4, 26.7; **HRMS** (ESI) *m/z*: $[M+H]^+$ Calcd for $C_9H_{10}NS$ 164.0534, Found 164.0522.

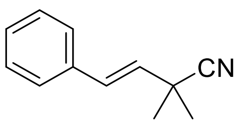

(*E*)-2,2-Dimethyl-4-phenylbut-3-enenitrile (3q). Yield: 95% (40.6 mg, 0.237 mmol), colorless oil, silica gel column chromatography (EtOAc:hexanes = 1:20). This compound has been previously reported, and the spectral data match the described data.[6] **^{1}H NMR** ($CDCl_3$, 400 MHz): δ 7.41-7.28 (m, 5H), 6.77 (d, *J* = 15.9 Hz, 2H), 6.04 (d, *J* = 15.9 Hz, 2H), 1.56 (s, 6H); **^{13}C{^{1}H} NMR** ($CDCl_3$, 100 MHz): δ 135.7, 130.4, 129.8, 128.7, 128.1, 126.5, 123.3, 34.9, 27.6.

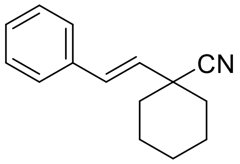

(*E*)-1-Styrylcyclohexane-1-carbonitrile (3r). Yield: 90% (47.5 mg, 0.225 mmol), pale yellow, silica gel column chromatography (EtOAc:hexanes = 1:20). This compound has been previously reported, and the spectral data match the described data.[6] **^{1}H NMR** ($CDCl_3$, 400 MHz): δ 7.41-7.39 (m, 2H), 7.34 (t, *J* = 7.3 Hz, 2H), 7.29-7.27 (m, 1H), 6.82 (d, *J* = 16.0 Hz, 1H), 6.03 (d, *J* = 16.0 Hz, 1H), 2.06-2.03 (m, 2H), 1.83-1.72 (m, 5H), 1.57-1.50 (m, 2H), 1.29-1.20 (m, 1H); **^{13}C{^{1}H} NMR** ($CDCl_3$, 100 MHz): δ 136.0, 130.4, 130.3, 128.6, 128.0, 126.5 122.0, 41.6, 36.3, 25.0, 22.8.

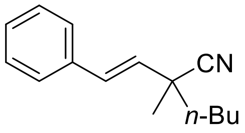

(*E*)-2-Methyl-2-styrylhexanenitrile (3s). Yield: 95% (50.4 mg, 0.236 mmol), colorless oil, silica gel column chromatography (EtOAc:hexanes = 1:20). **^{1}H NMR** ($CDCl_3$, 400 MHz): δ 7.40 (d, *J* = 7.8 Hz, 2H), 7.35 (t, *J* = 6.8 Hz, 2H), 7.29 (d, *J* = 7.3 Hz, 1H), 6.79 (d, *J* = 16.0 Hz, 1H), 5.92 (d, *J* = 16.0 Hz, 1H), 1.80-1.64 (m, 2H), 1.53 (s, 3H), 1.53-1.27 (m, 4H), 0.93 (t, *J* = 6.8 Hz, 3H); **^{13}C{^{1}H} NMR** ($CDCl_3$, 100 MHz): δ 135.8, 130.5, 129.6, 128.6, 128.0, 126.5, 122.6, 40.3, 40.0, 27.4, 26.3, 22.5, 13.8; **HRMS** (ESI) *m/z*: $[M+Na]^+$ Calcd for $C_{15}H_{19}NNa$ 236.1415, Found 236.1406.

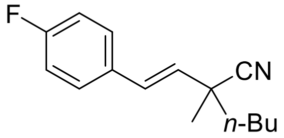

(*E*)-2-(4-Fluorostyryl)-2-methylhexanenitrile (3t). Yield: 94% (54.3 mg, 0.235 mmol), colorless oil, silica gel column chromatography (EtOAc:hexanes = 1:20). **^{1}H NMR** ($CDCl_3$, 400 MHz): δ 7.36

(6) Irudayanathan, F. M.; Lee S. *Org. Lett.* **2017**, *19*, 2318–2321.

(dd, J = 8.7, 5.5 Hz, 2H), 7.03 (t, J = 8.7 Hz, 2H), 7.29 (d, J = 7.3 Hz, 1H), 6.75 (d, J = 16.0 Hz, 1H), 5.84 (d, J = 16.0 Hz, 1H), 1.78-1.60 (m, 2H), 1.52 (s, 3H), 1.48-1.21 (m, 4H), 0.93 (t, J = 6.8 Hz, 3H); **^{13}C{^{1}H} NMR** ($CDCl_3$, 100 MHz): δ 162.5 (d, $J_{C\text{-}F}$ = 247.5 Hz), 132.0 (d, $J_{C\text{-}F}$ = 3.8 Hz), 129.4 (d, $J_{C\text{-}F}$ = 4.8 Hz), 128.1 (d, $J_{C\text{-}F}$ = 8.7 Hz), 127.0, 122.6, 115.5 (d, $J_{C\text{-}F}$ = 22.1 Hz), 40.3, 40.0, 27.5, 26.3, 22.5, 13.8; ^{19}F NMR ($CDCl_3$, 376 MHz): δ -113.54; **HRMS** (ESI) *m/z*: $[M+H]^+$ Calcd for $C_{15}H_{19}FN$ 232.1502, Found 232.1499.

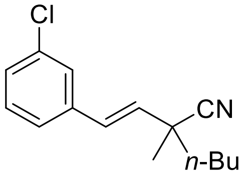

(*E*)-2-(3-Chlorostyryl)-2-methylhexanenitrile (3u). Yield: 97% (60.1 mg, 0.242 mmol), pale yellow oil, silica gel column chromatography (EtOAc:hexanes = 1:20). **^{1}H NMR** ($CDCl_3$, 400 MHz): δ 7.37 (s, 1H), 7.24-7.23 (m, 3H), 6.71 (d, J = 16.0 Hz, 1H), 5.91 (d, J = 16.0 Hz, 1H), 1.77-1.58 (m, 2H), 1.50 (s, 3H), 1.48-1.24 (m, 4H), 0.90 (t, J = 7.3 Hz, 3H); **^{13}C{^{1}H} NMR** ($CDCl_3$, 100 MHz): δ 137.6, 134.6, 131.2, 129.9, 129.3, 127.9, 126.2, 124.9, 122.3, 40.2, 40.1, 27.4, 26.2, 22.5, 13.8; **HRMS** (ESI) *m/z*: $[M+H]^+$ Calcd for $C_{15}H_{19}ClN$ 248.1206, Found 248.1197.

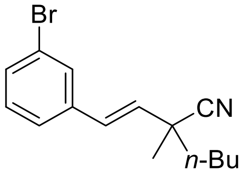

(*E*)-2-(3-Bromostyryl)-2-methylhexanenitrile (3v). Yield: 89% (64.8 mg, 0.222 mmol), pale yellow oil, silica gel column chromatography (EtOAc:hexanes = 1:20). **^{1}H NMR** ($CDCl_3$, 400 MHz): δ 7.56 (s, 1H), 7.42-7.39 (m, 1H), 7.30 (d, J = 7.8 Hz, 1H), 7.21 (d, J = 7.8 Hz, 1H), 6.72 (d, J = 16.0 Hz, 1H), 5.93 (d, J = 16.0 Hz, 1H), 1.78-1.60 (m, 2H), 1.52 (s, 3H), 1.50-1.33 (m, 4H), 0.93 (t, J = 7.3 Hz, 3H); **^{13}C{^{1}H} NMR** ($CDCl_3$, 100 MHz): δ 137.9, 131.2, 130.9, 130.2, 129.2, 129.1, 125.4, 122.8, 122.3, 40.2, 40.1, 27.4, 26.3, 22.5, 13.8; **HRMS** (ESI) *m/z*: $[M+Na]^+$ Calcd for $C_{15}H_{18}BrNNa$ 314.0520, Found 314.0516.

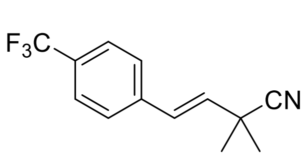

(*E*)-2,2-Dimethyl-4-(4-(trifluoromethyl)phenyl)but-3-enenitrile (3w). Yield: 90% (53.8 mg, 0.225 mmol), colorless oil, silica gel column chromatography (EtOAc:hexanes = 1:20). This compound has been previously reported, and the spectral data match the described data.[6] **^{1}H NMR** ($CDCl_3$, 400 MHz): δ 7.58 (d, J = 8.3 Hz, 2H), 7.40 (d, J = 8.3 Hz, 2H), 6.77 (d, J = 16.0 Hz, 1H), 6.14 (d, J =

16.0 Hz, 1H), 1.56 (s, 6H); **$^{13}C\{^1H\}$ NMR** ($CDCl_3$, 100 MHz): δ 139.4, 133.1, 129.9 (q, $J_{C\text{-}F}$ = 32.5 Hz), 128.4, 126.8, 126.2 (q, $J_{C\text{-}F}$ = 269.7 Hz), 125.6 (q, $J_{C\text{-}F}$ = 3.8 Hz), 123.6, 34.5, 27.7.

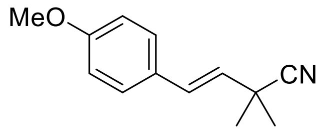

(*E*)-4-(4-Methoxyphenyl)-2,2-dimethylbut-3-enenitrile (3x). Yield: 90% (45.1 mg, 0.224 mmol), colorless oil, silica gel column chromatography (EtOAc:hexanes = 1:10). This compound has been previously reported, and the spectral data match the described data.[6] **^1H NMR** ($CDCl_3$, 400 MHz): 7.31 (d, J = 8.7 Hz, 2H), 6.85 (d, J = 8.7 Hz, 2H), 6.68 (d, J = 16.1 Hz, 1H), 5.89 (d, J = 16.0 Hz, 1H), 3.80 (s, 3H), 1.52 (s, 6H); **$^{13}C\{^1H\}$ NMR** ($CDCl_3$, 100 MHz): δ 159.5, 129.2, 128.4, 128.2, 127.7, 123.6, 114.0, 55.2, 34.8, 27.7.

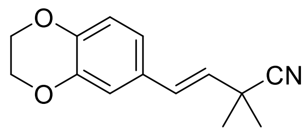

(*E*)-4-(2,3-Dihydrobenzo[b][1,4]dioxin-6-yl)-2,2-dimethylbut-3-enenitrile (3y). Yield: 93% (53.2 mg, 0.232 mmol), colorless oil, silica gel column chromatography (EtOAc:hexanes = 1:20). **^1H NMR** ($CDCl_3$, 400 MHz): δ 6.91-6.84 (m, 3H), 6.63 (d, J = 16.0 Hz, 1H), 5.88 (d, J = 16.0 Hz, 1H), 4.26 (s, 4H), 1.53 (s, 6H); **$^{13}C\{^1H\}$ NMR** ($CDCl_3$, 100 MHz): δ 143.7, 143.6, 129.5, 129.2, 128.8, 123.6, 120.0, 117.4, 115.1, 64.4, 64.3, 34.8, 27.7; **HRMS** (ESI) *m/z*: $[M+H]^+$ Calcd for $C_{14}H_{16}NO_2$ 230.1181, Found 230.1172.

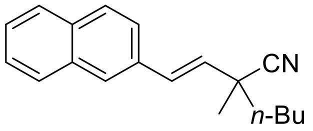

(*E*)-2-Methyl-2-(2-(naphthalen-2-yl)vinyl)hexanenitrile (3z). Yield: 85% (56.1 mg, 0.213 mmol), pale yellow oil, silica gel column chromatography (EtOAc:hexanes = 1:20). **^1H NMR** ($CDCl_3$, 400 MHz): δ 7.83-7.80 (m, 4H), 7.60-7.58 (m, 1H), 7.52-7.46 (m, 2H), 6.96 (d, J = 16.0 Hz, 1H), 6.06 (d, J = 15.6 Hz, 1H), 1.82-1.69 (m, 2H), 1.57 (s, 3H), 1.55-1.22 (m, 4H), 0.94 (t, J = 7.3 Hz, 3H); **$^{13}C\{^1H\}$ NMR** ($CDCl_3$, 100 MHz): δ 133.5, 133.2, 133.0, 130.7, 130.0, 128.3, 128.0, 127.6, 127.0, 126.8, 126.4, 126.1, 123.3, 40.4, 40.2, 27.5, 26.4, 22.6, 13.9; **HRMS** (ESI) *m/z*: $[M+H]^+$ Calcd for $C_{19}H_{22}N$ 264.1752, Found 264.1750.

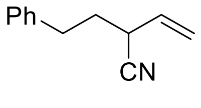

2-Phenethylbut-3-enenitrile (5a). Yield: 90% (38.6 mg, 0.225 mmol), colorless oil, silica gel column chromatography (EtOAc:hexanes = 1:20). This compound has been previously reported, and

the spectral data match the described data.[5] **^{1}H NMR** ($CDCl_3$, 400 MHz): δ 7.34-7.30 (m, 2H), 7.28-7.26 (m, 3H) 5.74 (ddd, J = 17.2, 10.0, 6.8 Hz, 1H), 5.45 (d, J = 17.2 Hz, 1H), 5.32 (d, J = 10.0 Hz, 1H), 3.30 (ddd, J = 6.8, 6.8, 6.8 Hz, 1H), 2.67–2.53 (m, 2H), 2.09–2.02 (m, 2H); **^{13}C{^{1}H} NMR** ($CDCl_3$, 100 MHz): δ 140.7, 131.8, 128.8, 128.6., 126.6, 119.8, 118.8, 34.5, 34.4, 32.9.

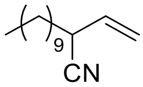

2-Vinyldodecanenitrile (5b). Yield: 79% (41.0 mg, 0.198 mmol), colorless oil, silica gel column chromatography (EtOAc:hexanes = 1:20). **^{1}H NMR** ($CDCl_3$, 400 MHz): δ 5.76-5.68 (m, 1H), 5.43 (dd, J = 17.0, 0.9 Hz, 1H), 5.28 (d, J = 10.0 Hz, 1H), 3.36 (q, J = 6.9 Hz, 1H), 1.71-1.64 (m, 2H), 1.53-1.39 (m, 2H), 1.33-1.21 (m, 14H), 0.89 (t, J = 6.4 Hz, 3H); **^{13}C{^{1}H} NMR** ($CDCl_3$, 100 MHz): δ 132.1, 120.0, 118.2, 35.1, 32.8, 31.9, 29.5, 29.5, 29.3, 29.3, 29.0, 26.7, 22.7, 14.1; **HRMS** (ESI) *m/z*: $[M+Na]^+$ Calcd for $C_{14}H_{25}NNa$ 230.1885, Found 230.1879.

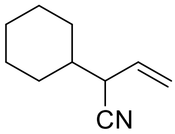

2-Cyclohexylbut-3-enenitrile (5c). Yield: 89% (33.1 mg, 0.222 mmol), colorless oil, silica gel column chromatography (EtOAc:hexanes = 1:20). This compound has been previously reported, and the spectral data match the described data.[7] **^{1}H NMR** ($CDCl_3$, 400 MHz): δ 5.71 (ddd, J = 17.0, 10.1, 6.0 Hz, 1H), 5.43 (d, J = 17.0 Hz, 1H), 5.32 (d, J = 10.1 Hz, 1H), 3.16 (m, 1H), 1.87-1.78 (m, 4H), 1.71-1.68 (m, 1H), 1.61-1.57 (m, 1H), 1.28-1.12 (m, 5H); **^{13}C{^{1}H} NMR** ($CDCl_3$, 100 MHz): δ 131.0, 119.2, 119.1, 42.1, 40.4, 31.0, 29.5, 26.0.

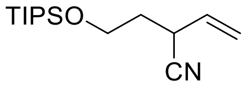

2-(2-((Triisopropylsilyl)oxy)ethyl)but-3-enenitrile (5d). Yield: 85% (56.8 mg, 0.212 mmol), colorless oil, silica gel column chromatography (EtOAc:hexanes = 1:20). This compound has been previously reported, and the spectral data match the described data.[7] **^{1}H NMR** ($CDCl_3$, 400 MHz): δ 5.79 (ddd, J = 17.0, 10.1, 6.0 Hz, 1H), 5.47 (dd, J = 17.0, 0.9 Hz, 1H), 5.30 (d, J = 10.1 Hz, 1H), 3.90-3.82 (m, 2H), 3.60 (q, J = 7.4 Hz, 1H), 1.93-1.87 (m, 2H), 1.09-1.05 (m, 21H); **^{13}C{^{1}H} NMR** ($CDCl_3$, 100 MHz): δ 132.3, 120.0, 118.5, 59.8, 36.2, 31.5, 18.0, 12.1.

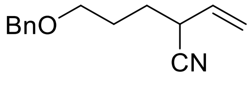

(7) Bury, T.; Kullmann, S.; Breit, B. *Adv. Synth. Catal.* **2023**, *365*, 335–341.

5-(Benzyloxy)-2-vinylpentanenitrile (5e). Yield: 73% (39.3 mg, 0.182 mmol), colorless oil, silica gel column chromatography (EtOAc:hexanes = 1:20). **^{1}H NMR** ($CDCl_3$, 400 MHz): δ 7.42-7.30 (m, 5H), 5.72 (ddd, *J* = 17.0, 10.1, 6.0 Hz, 1H), 5.44 (dd, *J* = 17.0, 1.4 Hz, 1H), 5.29 (dd, *J* = 10.1, 0.9 Hz, 1H), 4.51 (s, 2H), 3.55-3.50 (m, 2H), 3.31 (q, *J* = 5.5 Hz, 1H), 1.84-1.74 (m, 4H); **^{13}C{^{1}H} NMR** ($CDCl_3$, 100 MHz): δ 137.9, 135.2, 128.3, 127.6, 127.5, 122.0, 117.8, 72.9, 69.6, 49.7, 31.7, 26.0; **HRMS** (ESI) *m/z*: $[M+Na]^+$ Calcd for $C_{14}H_{17}NNaO$ 238.1208, Found 238.1202.

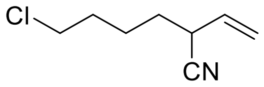

6-Chloro-2-vinylhexanenitrile (5f). Yield: 80% (31.7 mg, 0.201 mmol), colorless oil, silica gel column chromatography (EtOAc:hexanes = 1:20). **^{1}H NMR** ($CDCl_3$, 400 MHz): δ 5.73 (ddd, *J* = 17.0, 10.1, 6.0 Hz, 1H), 5.46 (dd, *J* = 17.0, 1.4 Hz, 1H), 5.32 (d, *J* = 10.1 Hz, 1H), 3.56 (t, *J* = 6.4 Hz, 2H), 3.30 (q, *J* = 6.4 Hz, 1H), 1.87-1.80 (m, 2H), 1.77-1.70 (m, 2H), 1.69-1.58 (m, 2H); **^{13}C{^{1}H} NMR** ($CDCl_3$, 100 MHz): δ 137.5, 121.1, 116.6, 44.1, 33.6, 30.3, 29.3, 26.2; **HRMS** (ESI) *m/z*: $[M+H]^+$ Calcd for $C_8H_{13}ClN$ 158.0737, Found 158.0728.

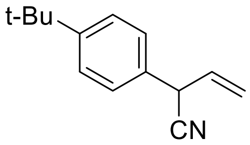

2-(4-(*tert*-Butyl)phenyl)but-3-enenitrile (5g). Yield: 90% (44.6 mg, 0.224 mmol), colorless oil, silica gel column chromatography (EtOAc:hexanes = 1:20). **^{1}H NMR** ($CDCl_3$, 400 MHz): δ 7.42 (d, *J* = 8.2 Hz, 2H), 7.27 (d, *J* = 8.5 Hz, 2H), 5.90 (ddd, *J* = 17.0, 10.1, 6.0 Hz, 1H), 5.54 (dd, *J* = 17.0, 1.4 Hz, 1H), 5.36 (dd, *J* = 10.1, 1.4 Hz, 1H), 4.51 (t, *J* = 6.0 Hz, 1H), 1.33 (s, 9H); **^{13}C{^{1}H} NMR** ($CDCl_3$, 100 MHz): δ 151.5, 132.3, 131.2, 130.2, 127.2, 126.2, 118.2, 40.3, 34.6, 31.2; **HRMS** (ESI) *m/z*: $[M+H]^+$ Calcd for $C_{14}H_{18}N$ 200.1439, Found 200.1441.

5. Procedure for gram-scale reaction

(*E*)-2,2-Dimethyl-4-phenylbut-3-enenitrile (3q). In a glove box, IPrCuCl (175 mg, 0.360 mmol) was added to a 100 mL round-bottom flask equipped with a magnetic stirring bar. The flask was sealed with a rubber septum fitted with a reflux condenser and removed from the glove box. After purging the flask with N_2gas for 5 minutes, THF (30.0 mL) and diisobutylaluminum hydride (1.28 mL, 7.2 mmol) were added via syringes. The reaction mixture was premixed for 10 minutes before a solution of (3-methylbuta-1,2-dien-1-yl)benzene (**1q**, 1.04 g, 7.20 mmol) in THF (6.0 mL) was added. The mixture was stirred at 60 °C on a preheated heating block for 3 hours. After cooling to room temperature, the reaction mixture was transferred via cannula to a separate 100 mL round-bottom flask containing *p*-toluenesulfonyl cyanide (1.09 g, 6.00 mmol). The mixture was stirred at 22 °C for 0.5 hours. The reaction was quenched by adding 1 N aqueous HCl (15 mL) and extracted with ethyl acetate (3 × 15 mL). The combined organic layers were dried over $MgSO_4$, filtered, and concentrated under reduced pressure. The crude product was purified by silica gel column chromatography (EtOAc/hexanes 1:20) to yield the desired product, **3q** (956 mg, 5.58 mmol, 93%), as a colorless oil.

2-Vinyldodecanenitrile (5b). In a glove box, **IPrCuCl** (146 mg, 0.300 mmol) was added to a 100 mL round-bottom flask equipped with a magnetic stirring bar. The flask was sealed with a rubber septum fitted with a reflux condenser and removed from the glove box. After purging the flask with N_2gas for 5 minutes, THF (24.0 mL) and diisobutylaluminum hydride (1.07 mL, 6.00 mmol) were added via syringes. The reaction mixture was premixed for 10 minutes before a solution of (3-methylbuta-1,2-dien-1-yl)benzene (**4b**, 1.08 g, 6.00 mmol) in THF (6.0 mL) was added. The mixture was stirred at 60 °C on a preheated heating block for 3 hours. After cooling to room temperature, the reaction mixture was transferred via cannula to a separate 100 mL round-bottom flask containing *p*-toluenesulfonyl cyanide (906 mg, 5.00 mmol). The mixture was stirred at 22 °C for 0.5 hours. The reaction was quenched by adding 1 N aqueous HCl (15 mL) and extracted with ethyl acetate (3 × 15 mL). The combined organic layers were dried over $MgSO_4$, filtered, and concentrated under reduced pressure. The crude product was purified by silica gel column chromatography (EtOAc/hexanes 1:20) to yield the desired product, **5b** (902 mg, 4.35 mmol, 87%), as a colorless oil.

6. Synthetic applications

(1) Synthesis of an amide **6**

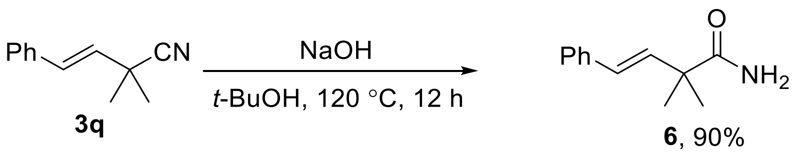

(*E*)-2,2-Dimethyl-4-phenylbut-3-enamide (6). (*E*)-2,2-Dimethyl-4-phenylbut-3-enenitrile (**3q**, 17.1 mg, 0.10 mmol), NaOH (1.20 mg, 0.30 mmol) and *tert*-butanol (0.1 mL) were added to an 8 mL Kimax vial equipped with a stirring bar. The vial was sealed with a phenolic open-top cap fitted with a gray PTFE/silicone septum, and the reaction mixture was stirred at 120 °C on a preheated heating block for 24 hours. After completion, the reaction mixture was cooled to room temperature, and H_2O (0.5 mL) was added. The resulting mixture was extracted with ethyl acetate (3 × 1 mL). The organic layers were combined, dried over anhydrous $MgSO_4$, filtered, and concentrated in vacuo. The crude product was purified by silica gel column chromatography (EtOAc/hexanes 1:1) to obtain amide **6** (17.0 mg, 0.090 mmol, 90%) as a white solid. This compound has been previously reported, and the spectral data match the described data.[8] **^{1}H NMR** ($CDCl_3$, 400 MHz): δ 7.39 (d, *J* = 7.3 Hz, 2H), 7.32 (t, *J* = 7.5 Hz, 2H), 7.27-7.23 (m, 1H), 6.55 (d, *J* = 16.3 Hz, 1H), 6.38 (d, *J* = 16.3 Hz, 1H), 5.70 (br s, 1H), 5.57 (br s, 1H), 1.41 (s, 6H); **^{13}C{^{1}H} NMR** ($CDCl_3$, 100 MHz): δ 178.8, 136.5, 134.5, 129.2, 128.7, 127.8, 126.3, 44.8, 25.2.

(2) Synthesis of amine **7**

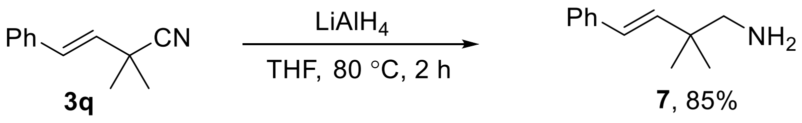

(*E*)-2,2-Dimethyl-4-phenylbut-3-en-1-amine (7). (*E*)-2,2-Dimethyl-4-phenylbut-3-enenitrile (**3q**, 17.1 mg, 0.100 mmol) and $LiAlH_4$ (4.90 mg, 0.130 mmol) were added to an 8 mL Kimax vial equipped with a stirring bar. The vial was sealed with a phenolic open-top cap fitted with a gray PTFE/silicone septum, and THF (0.2 mL) was added under an N_2 atmosphere. The reaction mixture was stirred at 80 °C on a preheated heating block for 2 hours. After cooling to room temperature, a 10% NaOH solution (0.5 mL) was added dropwise. The resulting mixture was extracted with ethyl acetate (3 × 1 mL). The combined organic layers were dried over anhydrous $MgSO_4$, filtered, and

(8) Cao, Z.-z.; Nie, Z.; Yang, T.; Su, M.; Li, H.; Luo, W.-p.; Liu, Q.; Guo, C.-C. *J. Org. Chem.* **2020**, *85*, 3287−3296.

concentrated under reduced pressure. The crude product was purified by silica gel column chromatography (EtOAc/hexanes 1:3), yielding the desired amine **7** (15.0 mg, 0.085 mmol, 85%) as a colorless oil. **^{1}H NMR** ($CDCl_3$, 400 MHz): δ 7.39-7.37 (m, 2H), 7.33-7.29 (m, 2H), 7.23-7.19 (m, 1H), 6.35 (d, *J* = 16.5 Hz, 1H), 6.14 (d, *J* = 16.0 Hz, 1H), 2.59 (s, 2H), 1.10 (s, 6H) ; **^{13}C{^{1}H} NMR** ($CDCl_3$, 100 MHz): δ 138.2, 136.5, 129.2, 128.8, 127.8, 126.3, 55.3, 36.2, 25.0; **HRMS** (ESI) *m/z*: $[M+H]^+$ Calcd for $C_{12}H_{18}N$ 176.1439, Found 176.1432.

(3) Synthesis of 2,2-dimethyl-4-phenylbutanenitrile (**8**)

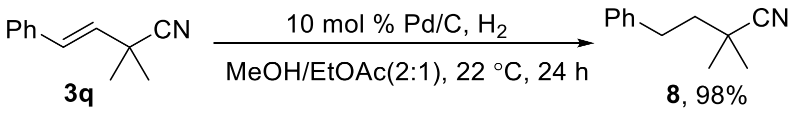

2,2-Dimethyl-4-phenylbutanenitrile (8). (*E*)-2,2-Dimethyl-4-phenylbut-3-enenitrile (**3q**, 17.1 mg, 0.100 mmol) and Pd/C (1.06 mg, 10 mol%) were added to an 8 mL Kimax vial equipped with a stirring bar. The vial was sealed with a phenolic open-top cap fitted with a gray PTFE/silicone septum, and a mixture of methanol and ethyl acetate (2:1, 2 mL) was added under an N_2 atmosphere. The reaction mixture was stirred at 22 °C for 24 hours. After completion, the solvent was removed under reduced pressure. The crude mixture was filtered through a plug of Celite and eluted with diethyl ether. The filtrate was concentrated under reduced pressure to afford the desired product, **8** (17.2 mg, 0.099 mmol, 98%), as a colorless oil. This compound has been previously reported, and the spectral data match the described data.[9] **^{1}H NMR** ($CDCl_3$, 400 MHz): δ 7.34-7.30 (m, 2H), 7.24-7.21 (m, 3H), 2.84-2.80 (m, 2H), 1.86-1.82 (m, 2H), 1.42 (s, 6H); **^{13}C{^{1}H} NMR** ($CDCl_3$, 100 MHz): δ 140.8, 128.6, 128.3, 126.2, 124.8, 43.0, 32.4, 31.8, 26.7.

(4) Synthesis of lactam **9**

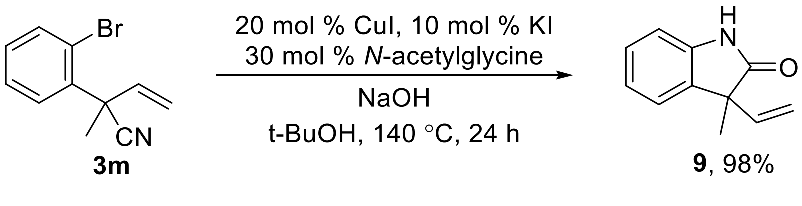

3-Methyl-3-vinylindolin-2-one (9). Nitrile **3m** (39.1 mg, 0.17 mmol) and NaOH (157 mg, 0.68 mmol) were added to an 8 mL Kimax vial equipped with a stirring bar. The vial was sealed with a phenolic open-top cap fitted with a gray PTFE/silicone septum, and *tert*-BuOH (0.2 mL) was added under an

(9) Lee, S.; Kang, G.; Han, S. *Org. Lett.* **2024**, *26*, 5640−5645.

N_2 atmosphere. The reaction mixture was stirred at 140 °C on a preheated heating block for 2 hours. After cooling to room temperature, CuI (5.50 mg, 20 mol %), KI (2.80 mg, 10 mol %), and *N*-acetylglycine (8.40 mg, 30 mol %) were added to the vial. The reaction mixture was stirred at 140 °C for an additional 22 hours. Upon completion, the reaction was cooled to room temperature, and distilled water (1 mL) was added. The mixture was extracted with ethyl acetate (3 × 1 mL). The combined organic layers were dried over anhydrous $MgSO_4$, filtered, and concentrated under reduced pressure. The crude product was purified to yield the desired lactam **9** as a brown solid (28.9 mg, 0.167 mmol, 98%). This compound has been previously reported, and the spectral data match the described data.[10] **^{1}H NMR** ($CDCl_3$, 400 MHz): δ 8.70 (s, 1H), 7.26-7.22 (m, 1H), 7.19-7.17 (m, 1H), 7.07 (t, *J* = 7.4 Hz, 1H), 6.96 (d, *J* = 7.8 Hz, 1H), 5.98 (dd, *J* = 17.2, 10.3 Hz, 1H), 5.22-5.16 (m, 2H), 1.53 (s, 3H); **^{13}C{^{1}H} NMR** ($CDCl_3$, 100 MHz): δ 181.7, 140.2, 137.7, 133.2, 128.0, 124.1, 122.5, 115.6, 110.2, 51.8, 22.3.

(10) Ueda, S.; Okada, T.; Nagasawa, H. *Chem. Commun.* **2010**, 46, 2462–2464.

7. Copies of ^{1}H and ^{13}C NMR spectra for all products

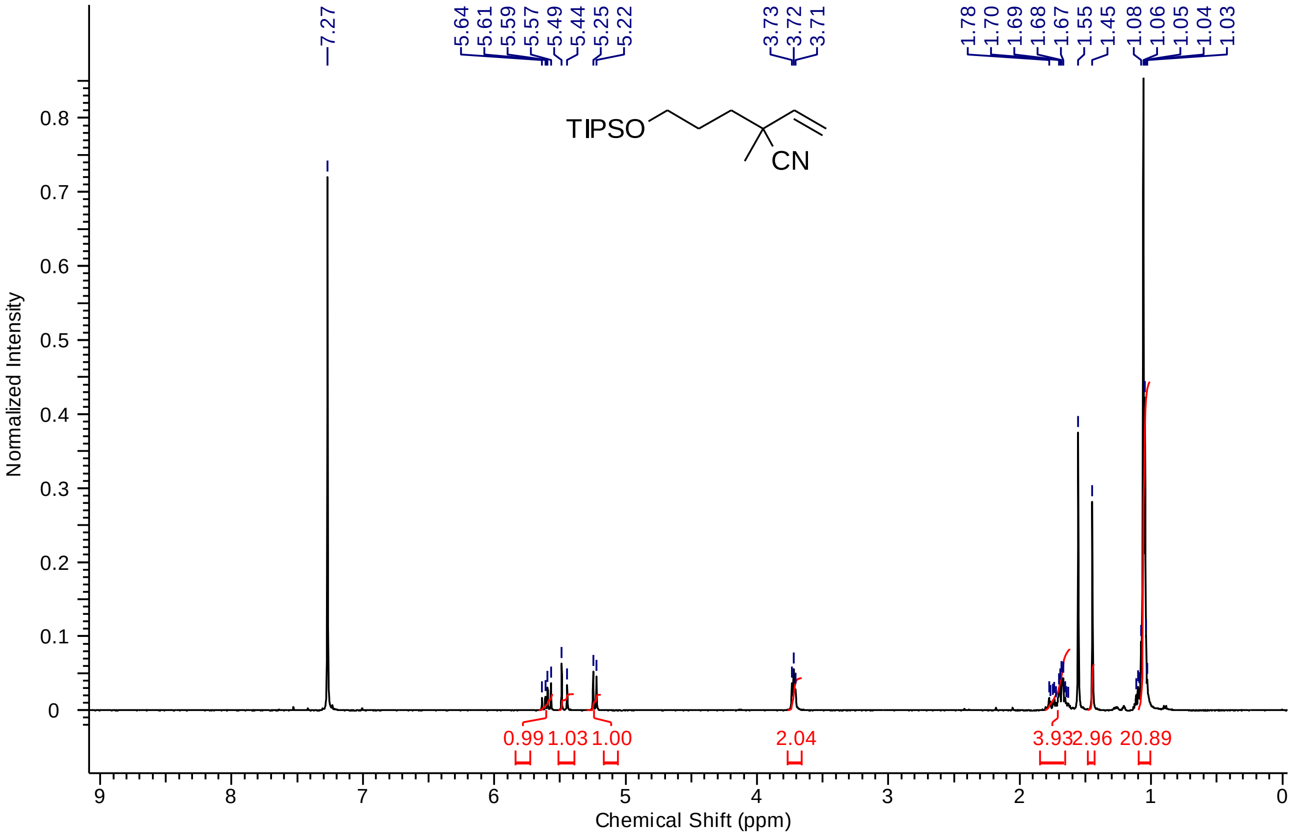

^{1}H NMR spectrum of the compound **3a** in $CDCl_3$, 400 MHz

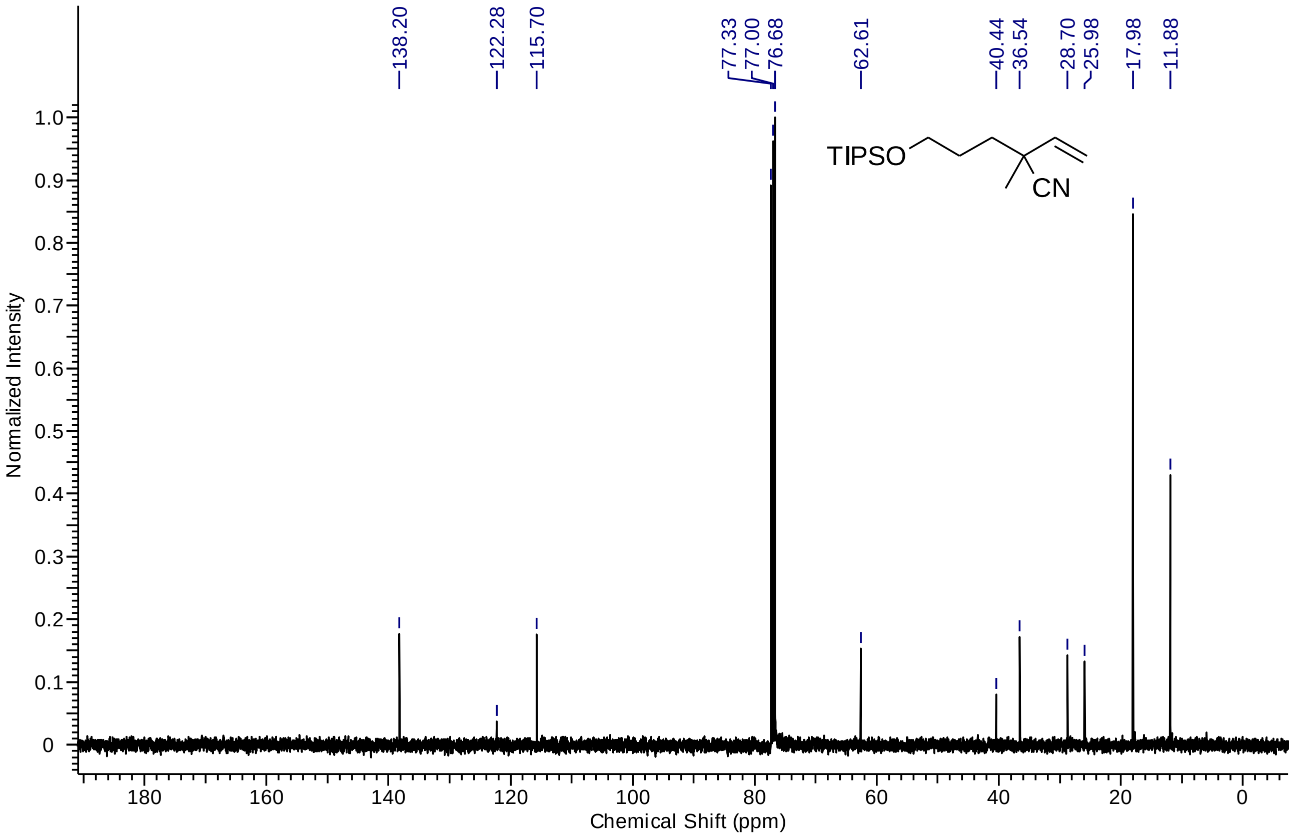

^{13}C NMR spectrum of the compound **3a** in $CDCl_3$, 100 MHz

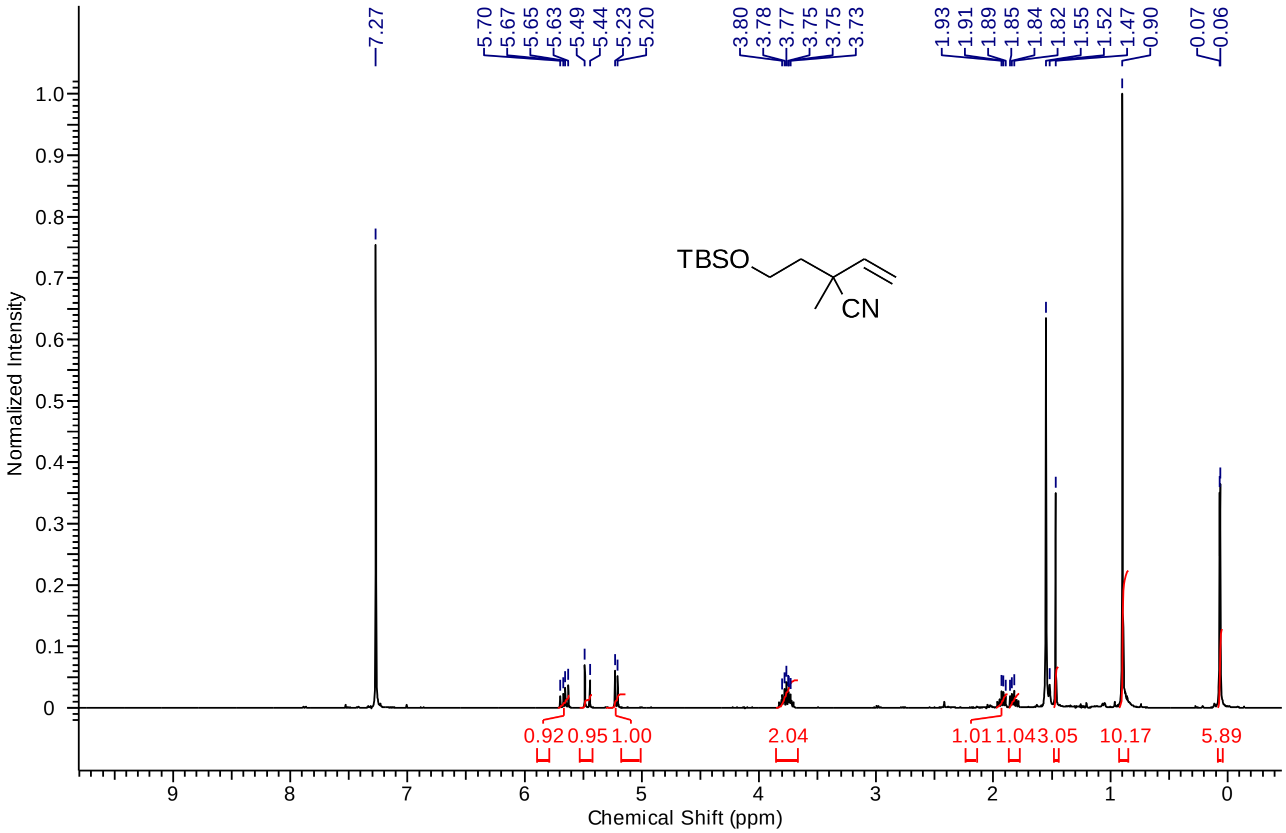

^{1}H NMR spectrum of the compound **3b** in $CDCl_3$, 400 MHz

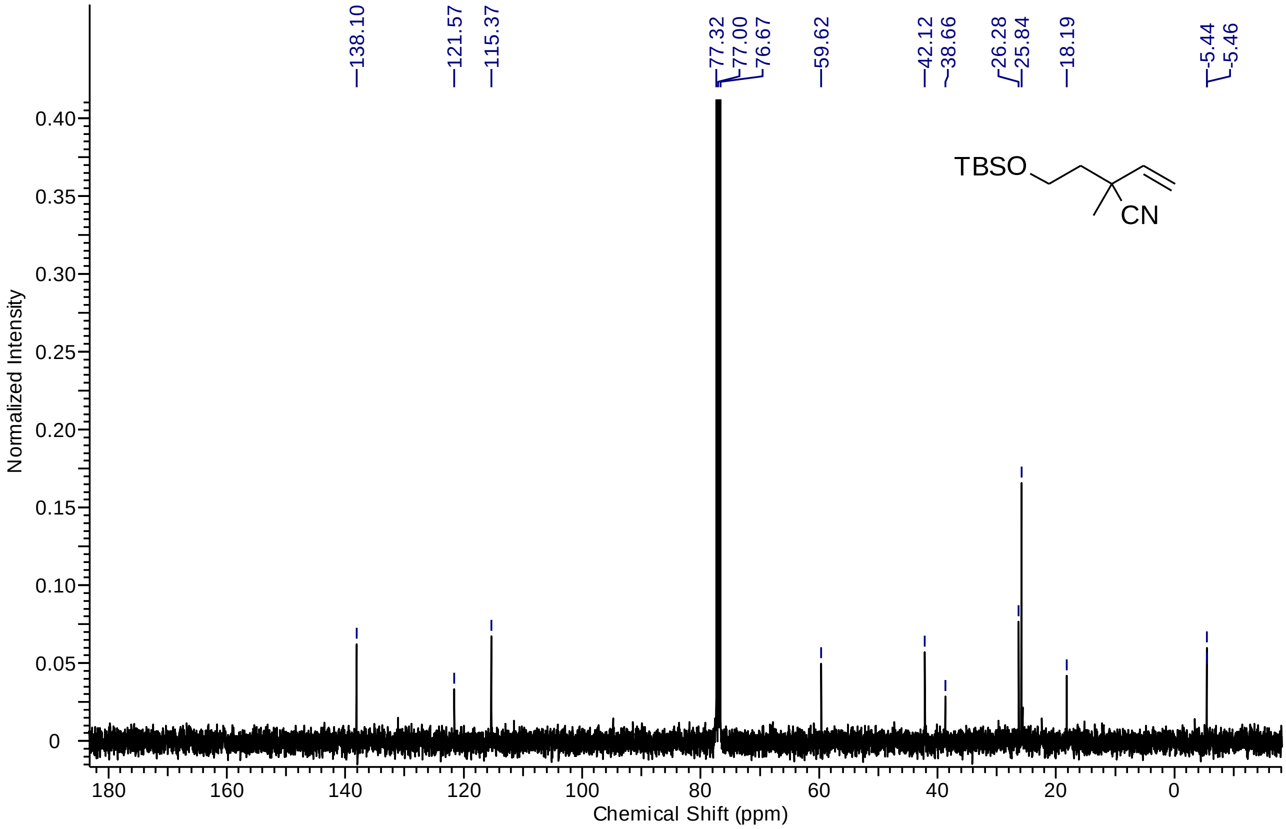

^{13}C NMR spectrum of the compound **3b** in $CDCl_3$, 100 MHz

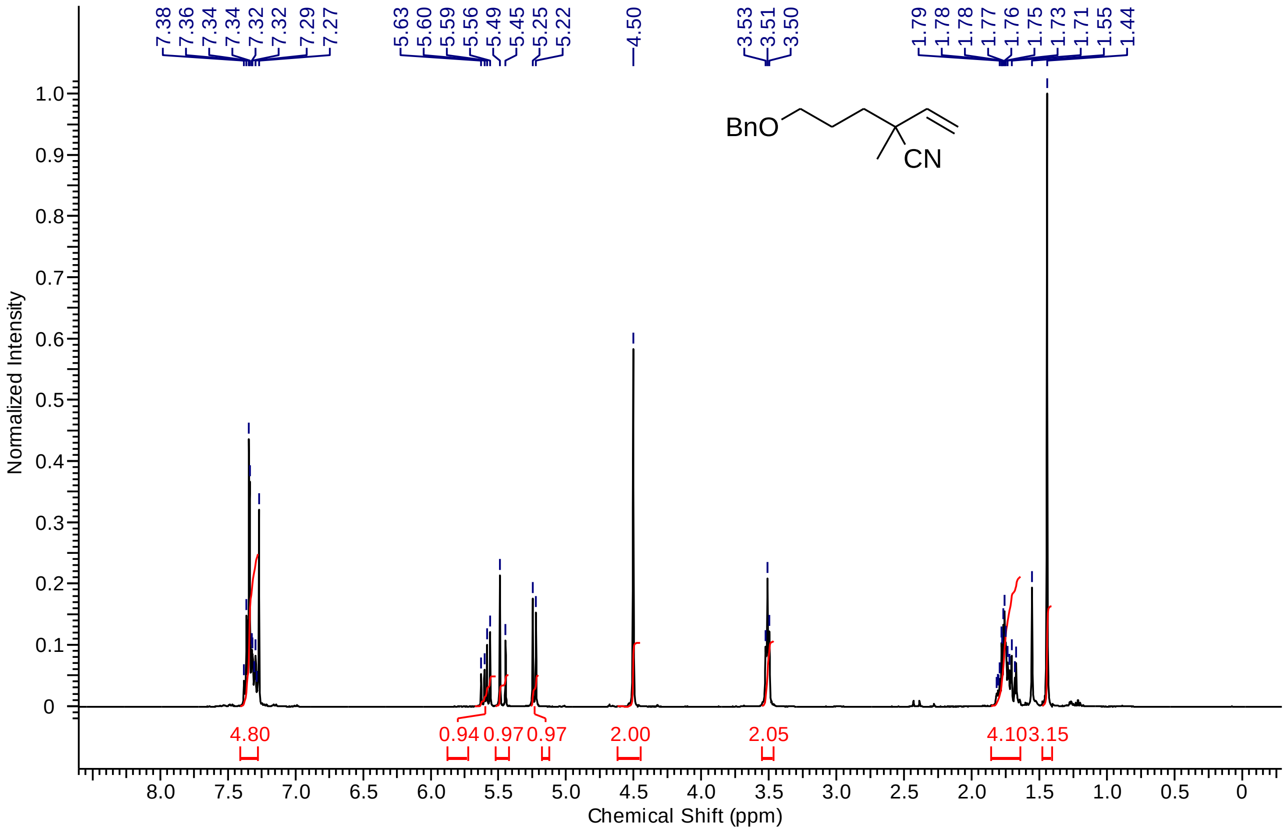

^{1}H NMR spectrum of the compound **3c** in $CDCl_3$, 400 MHz

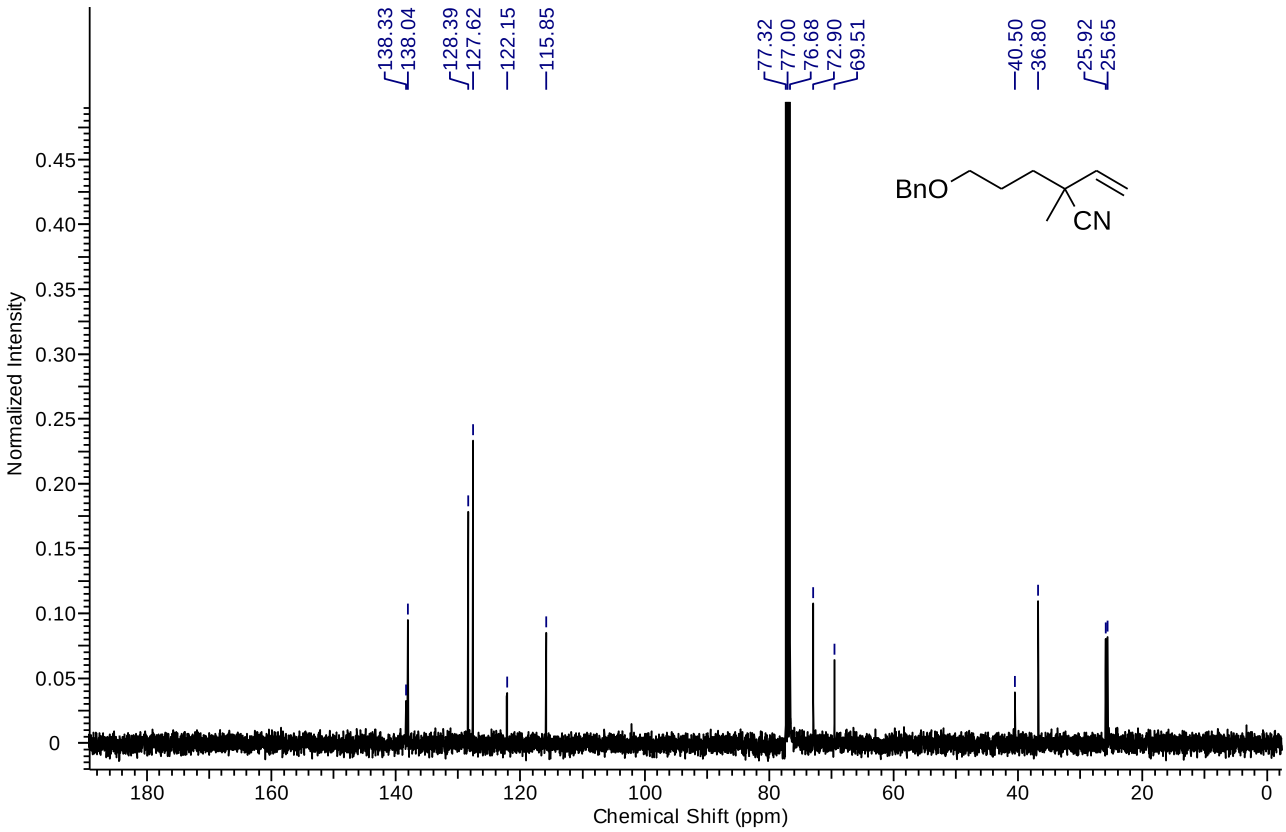

^{13}C NMR spectrum of the compound **3c** in $CDCl_3$, 100 MHz

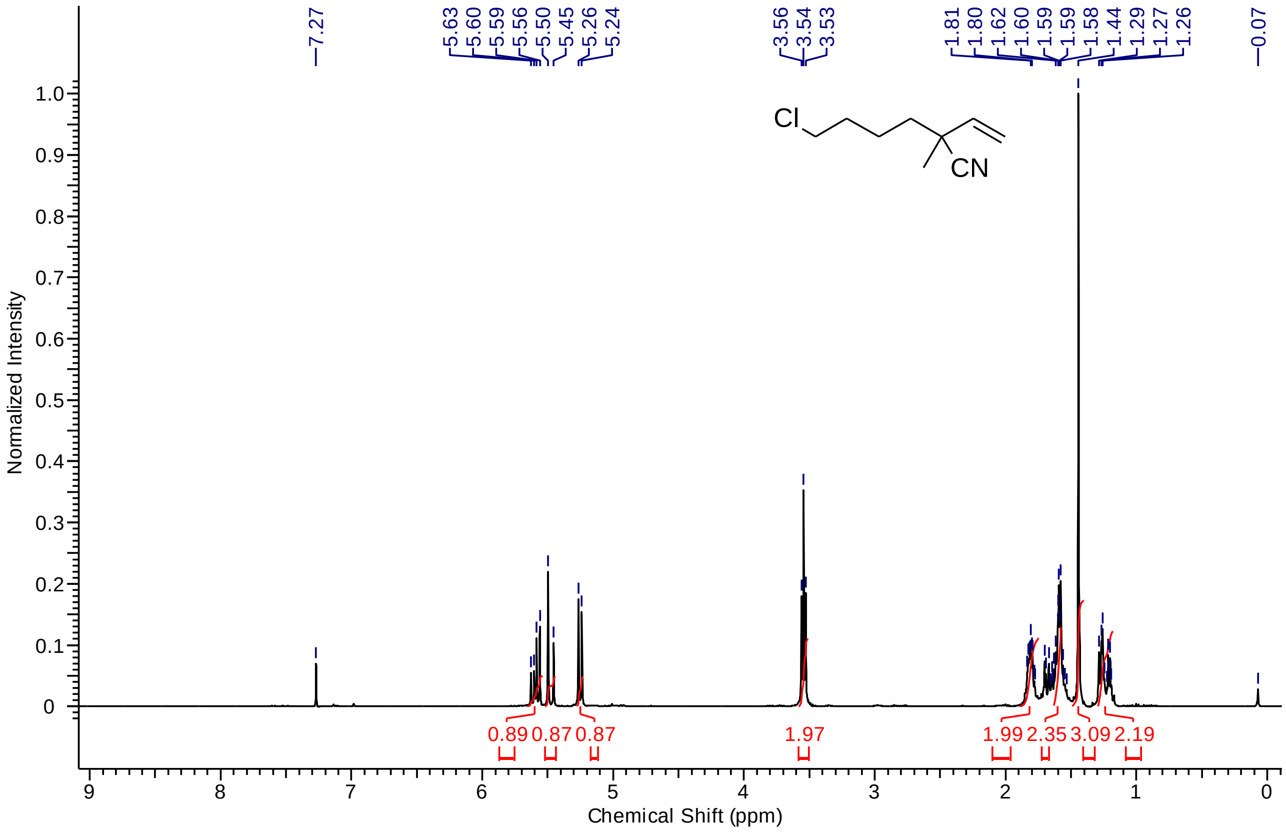

^{1}H NMR spectrum of the compound **3d** in $CDCl_3$, 400 MHz

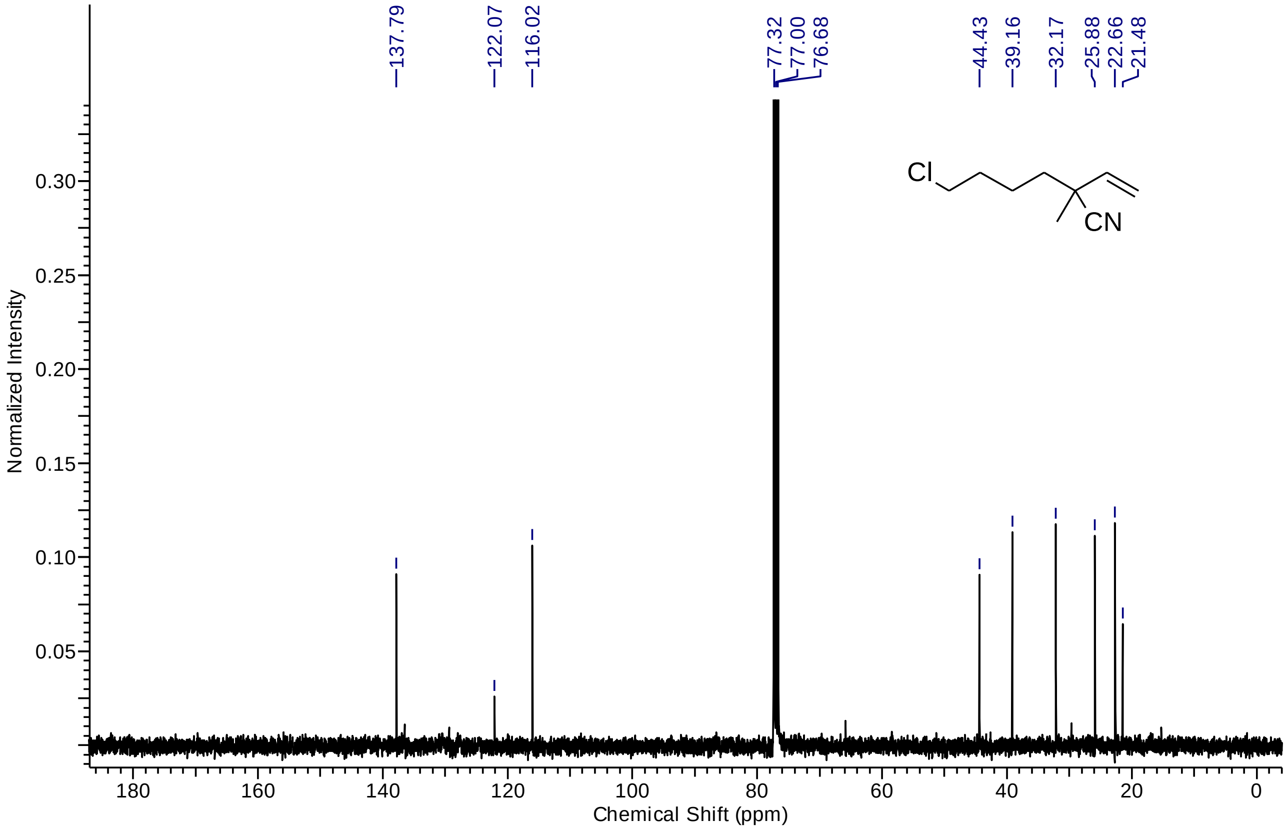

^{13}C NMR spectrum of the compound **3d** in $CDCl_3$, 100 MHz

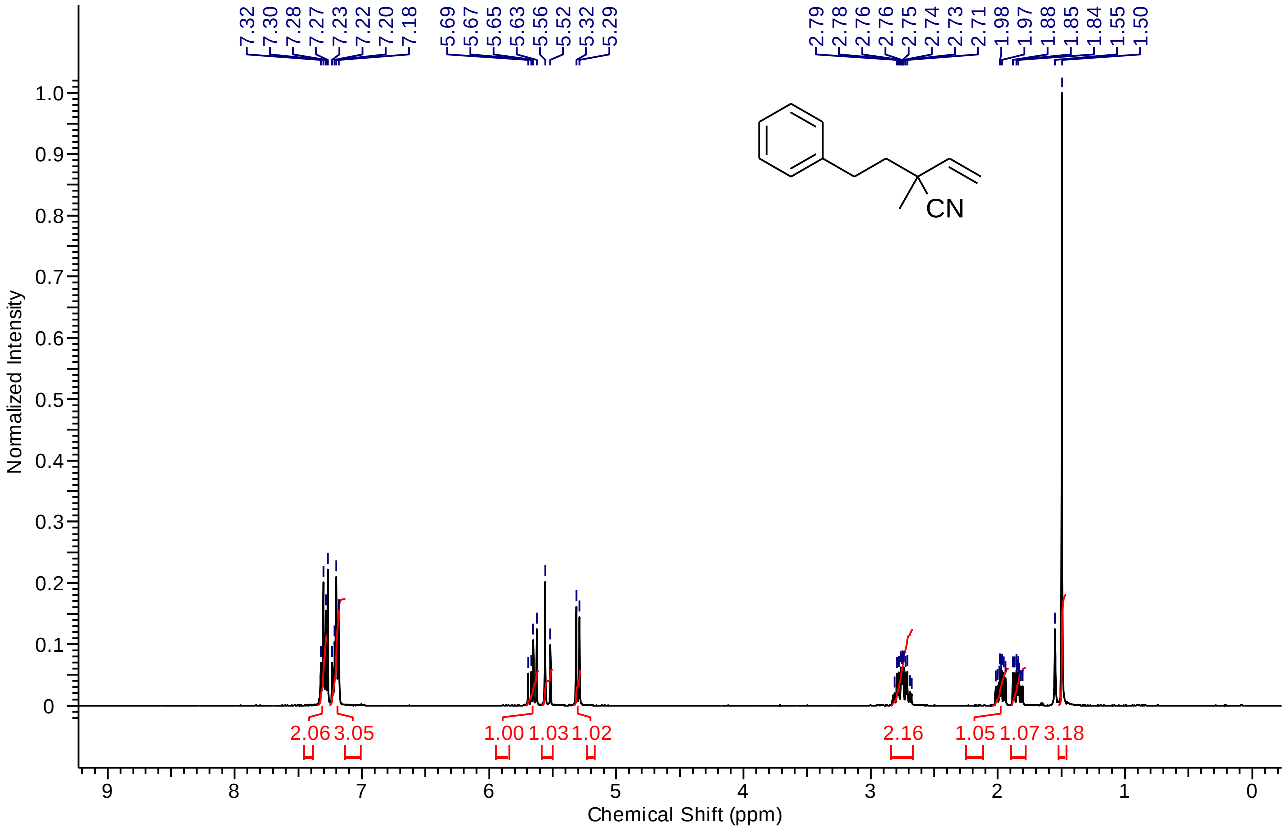

^{1}H NMR spectrum of the compound **3e** in $CDCl_3$, 400 MHz

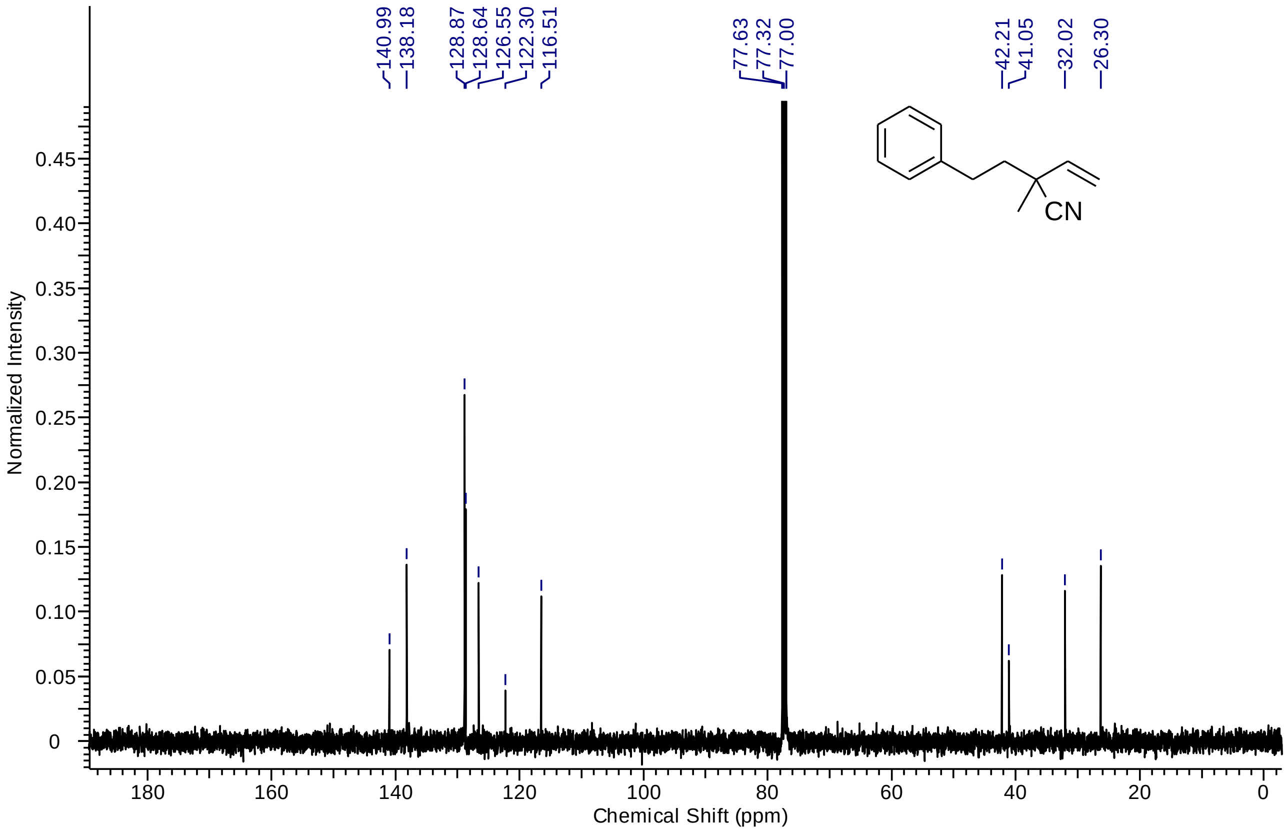

^{13}C NMR spectrum of the compound **3e** in $CDCl_3$, 100 MHz

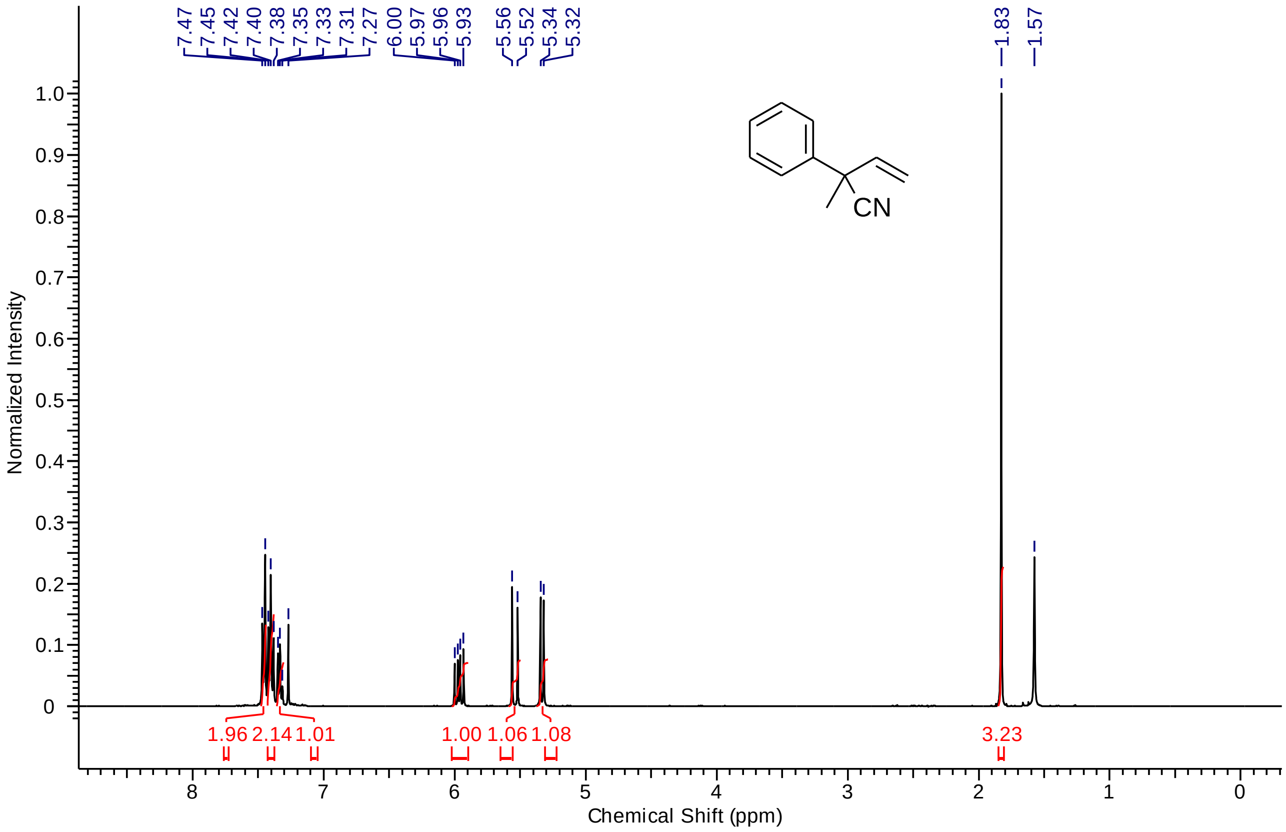

^{1}H NMR spectrum of the compound **3f** in $CDCl_3$, 400 MHz

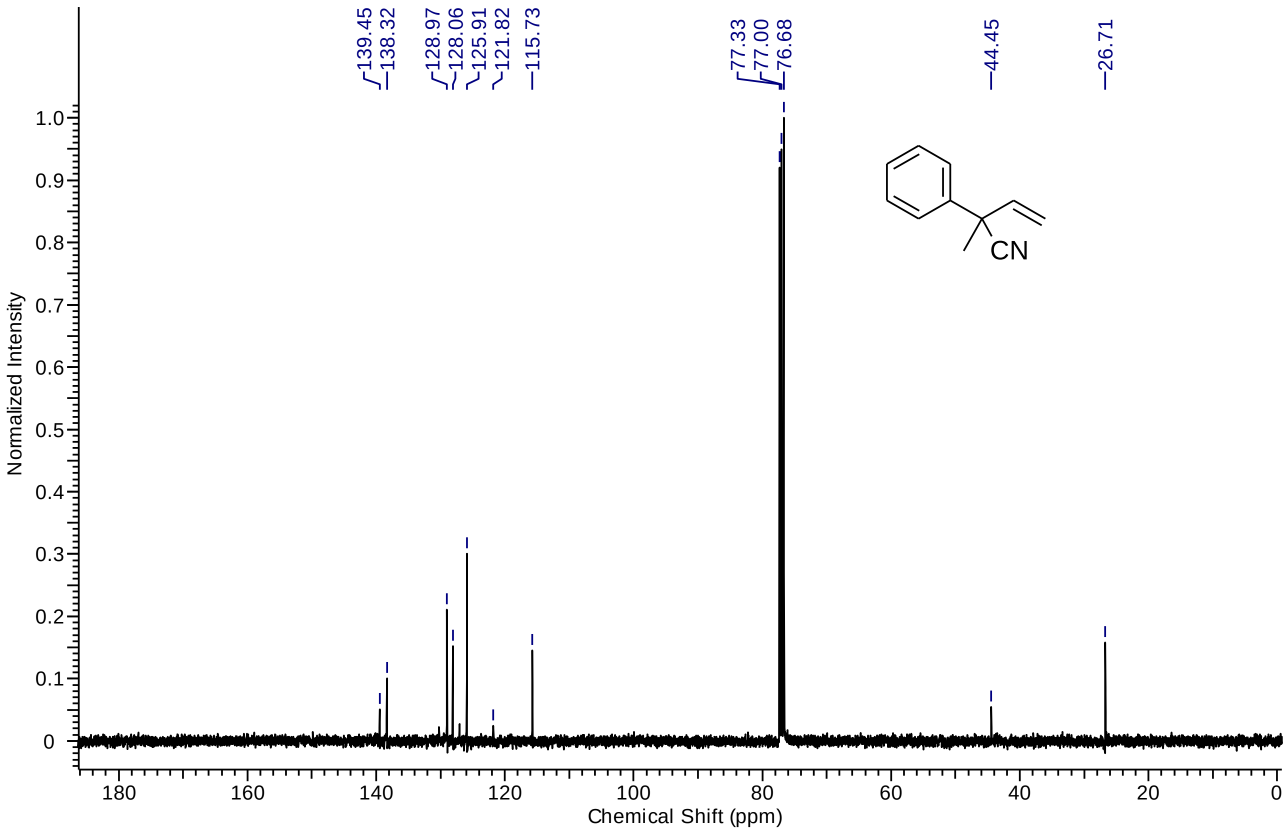

^{13}C NMR spectrum of the compound **3f** in $CDCl_3$, 100 MHz

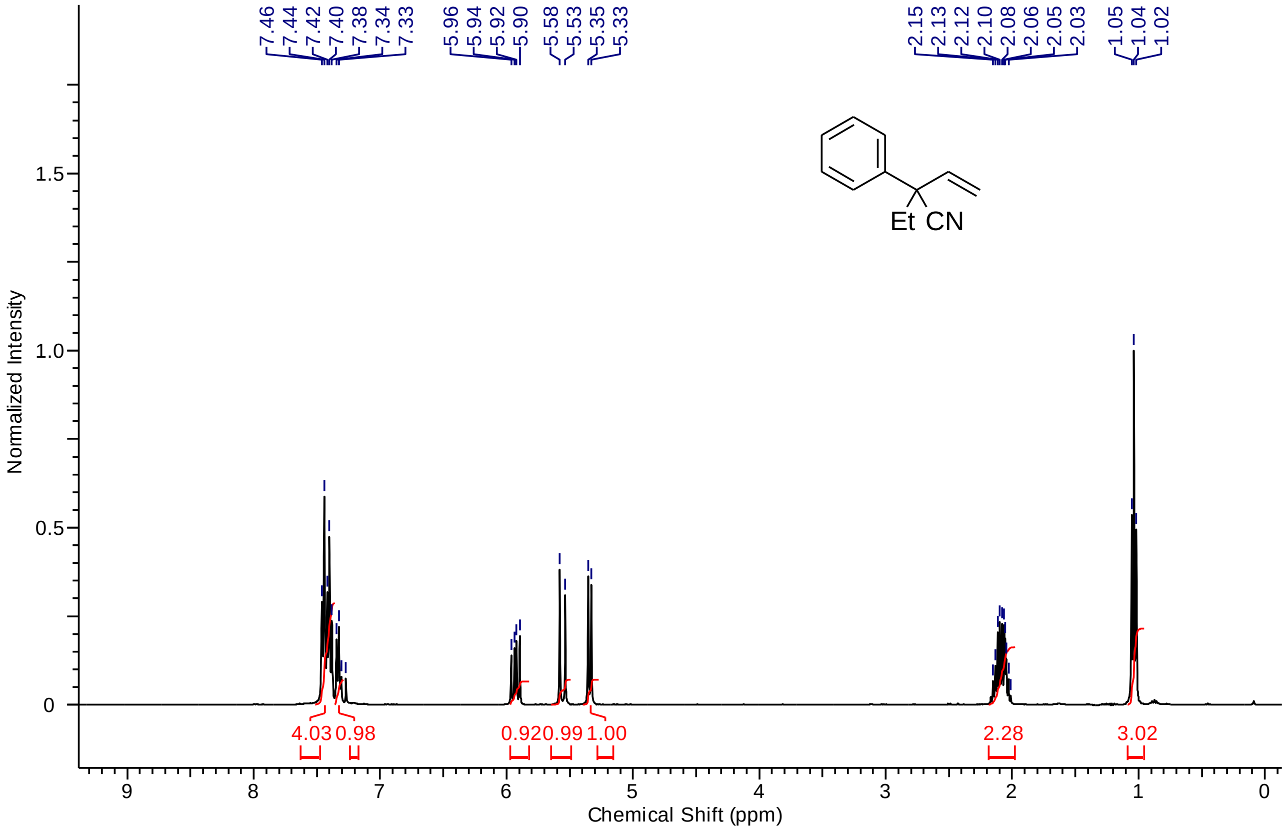

^{1}H NMR spectrum of the compound **3g** in $CDCl_3$, 400 MHz

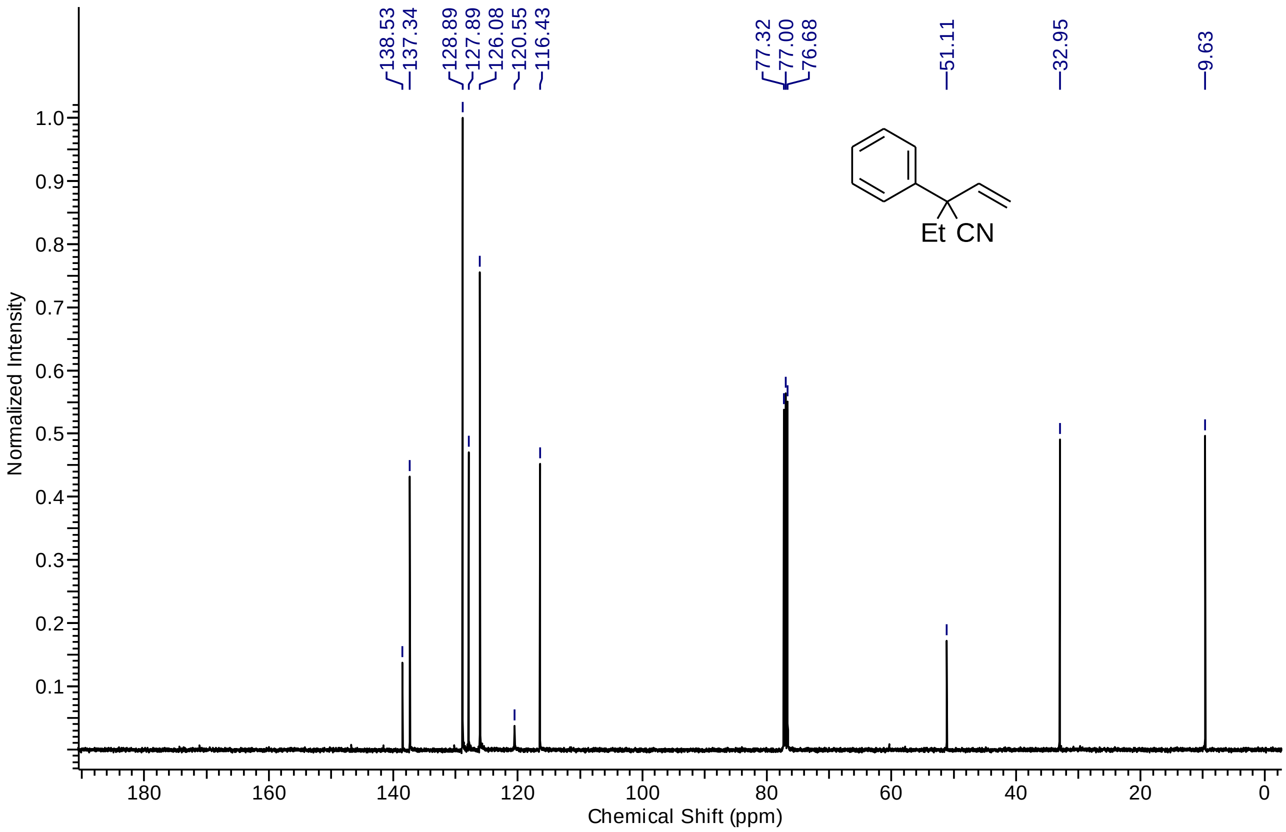

^{13}C NMR spectrum of the compound **3g** in $CDCl_3$, 100 MHz

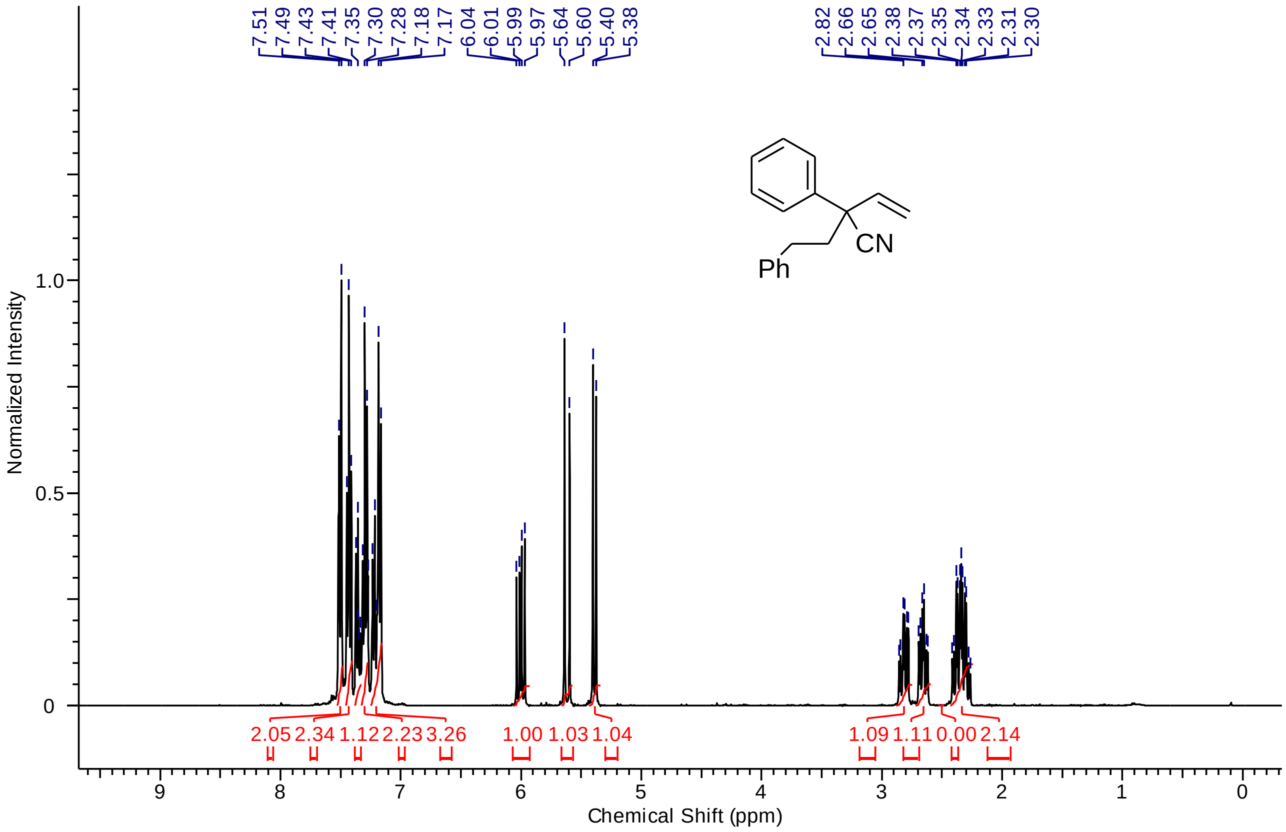

^{1}H NMR spectrum of the compound **3h** in $CDCl_3$, 400 MHz

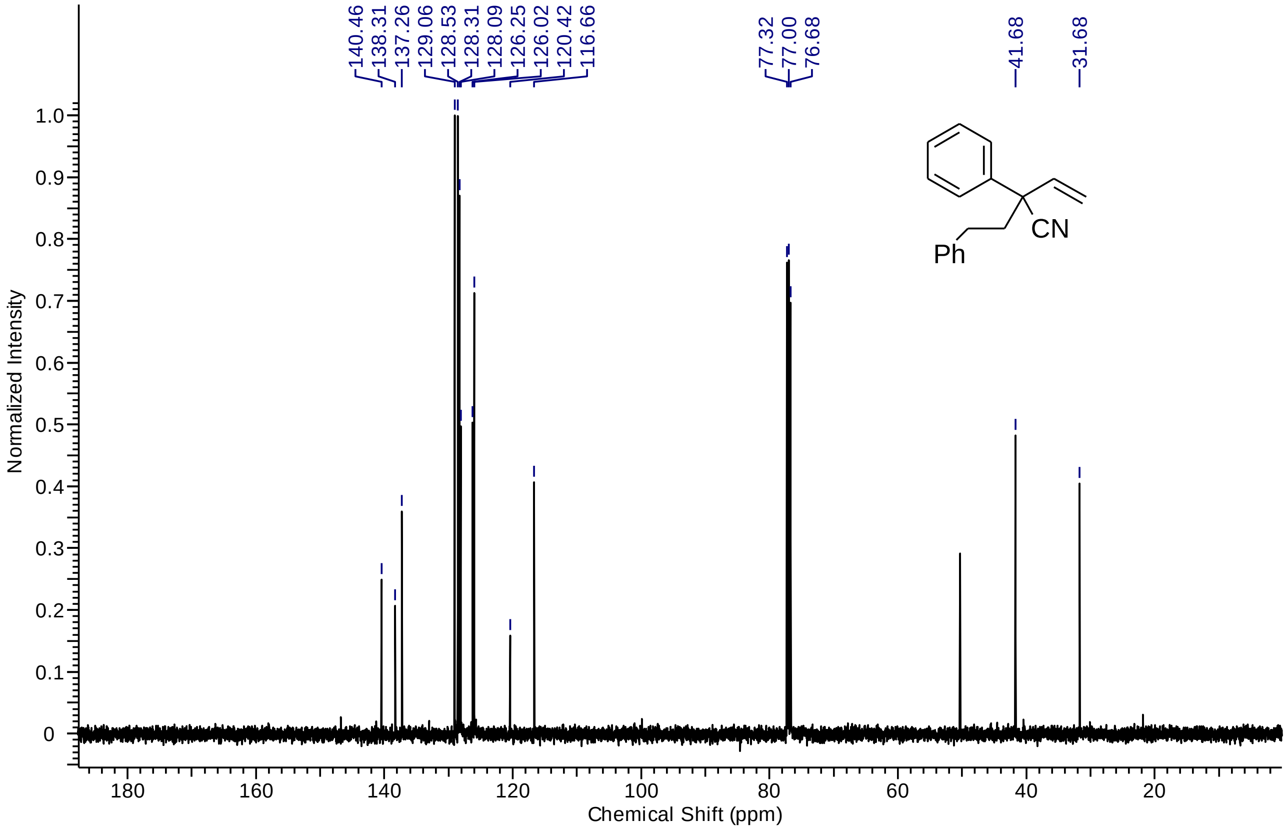

^{13}C NMR spectrum of the compound **3h** in $CDCl_3$, 100 MHz

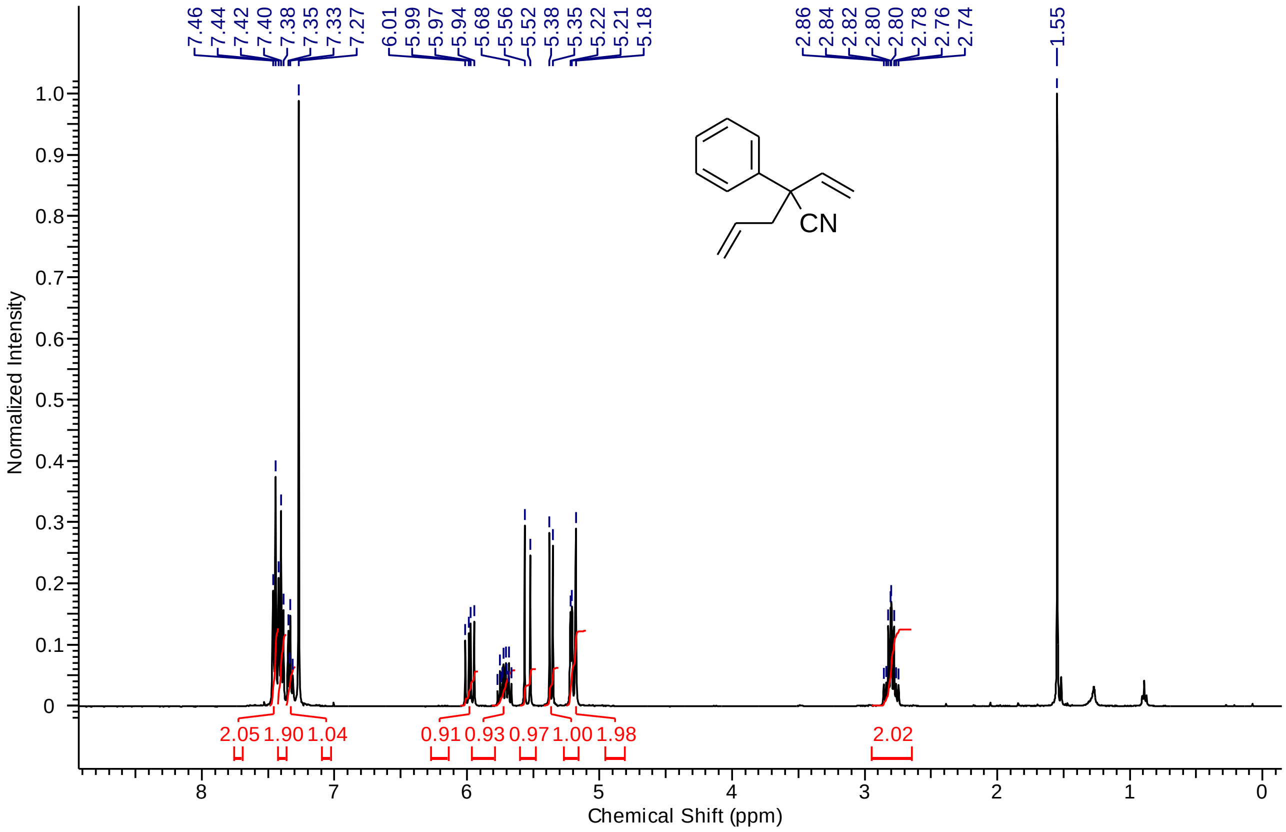

^{1}H NMR spectrum of the compound **3i** in $CDCl_3$, 400 MHz

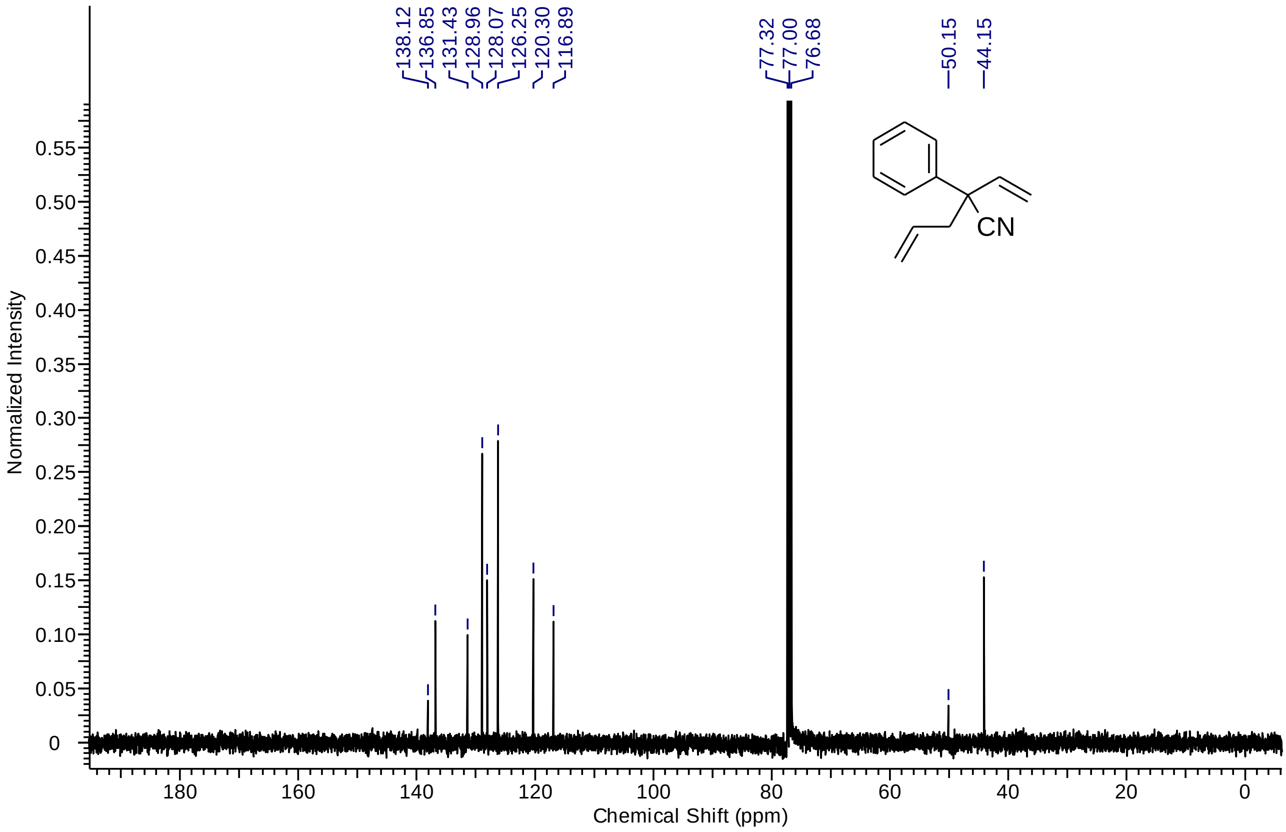

^{13}C NMR spectrum of the compound **3i** in $CDCl_3$, 100 MHz

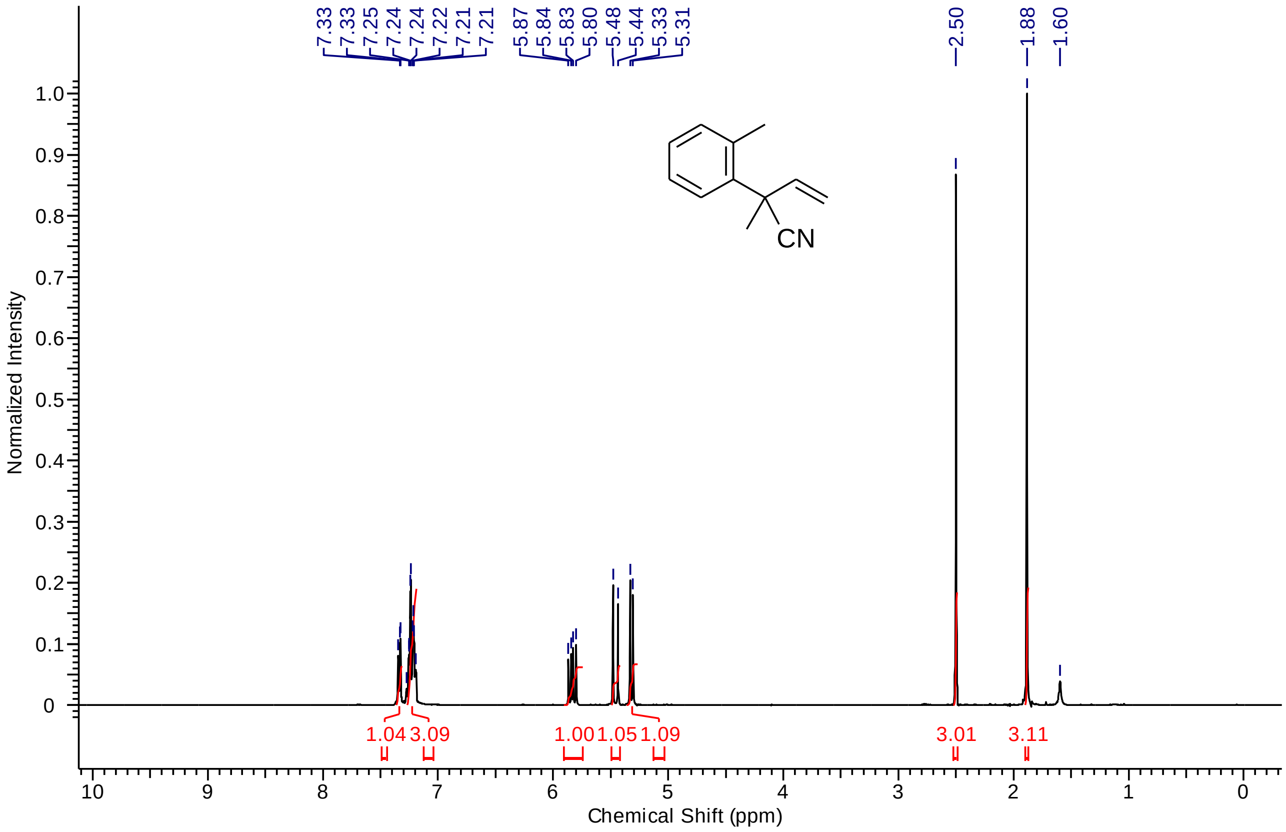

^{1}H NMR spectrum of the compound **3j** in $CDCl_3$, 400 MHz

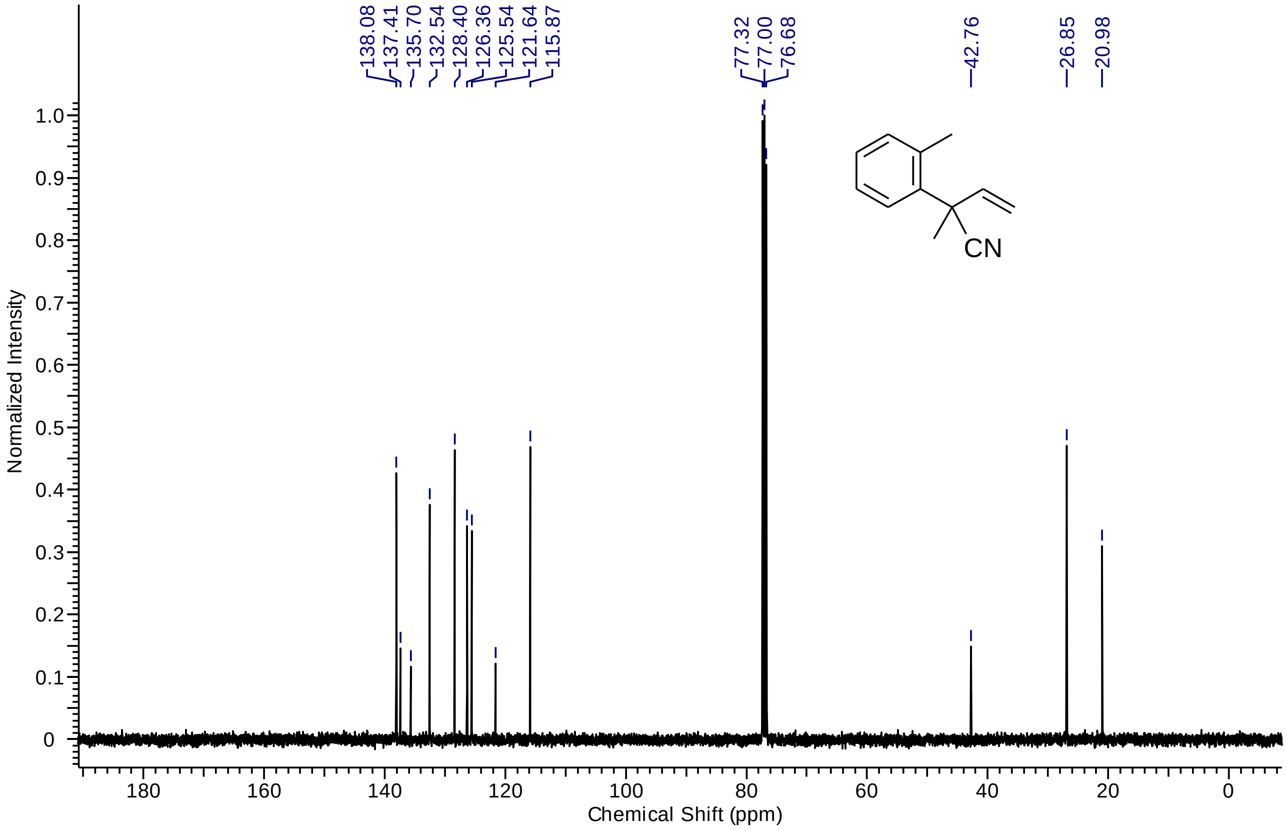

^{13}C NMR spectrum of the compound **3j** in $CDCl_3$, 100 MHz

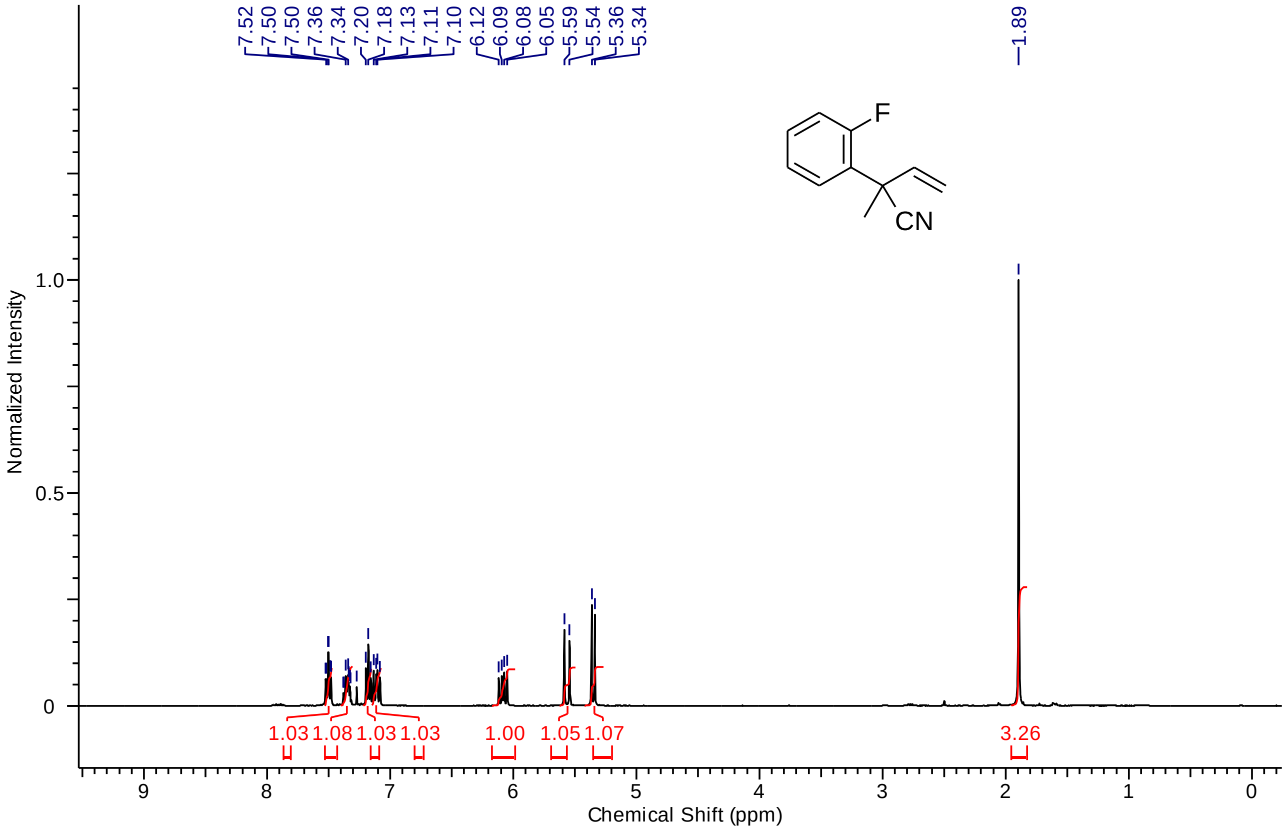

^{1}H NMR spectrum of the compound **3k** in $CDCl_3$, 400 MHz

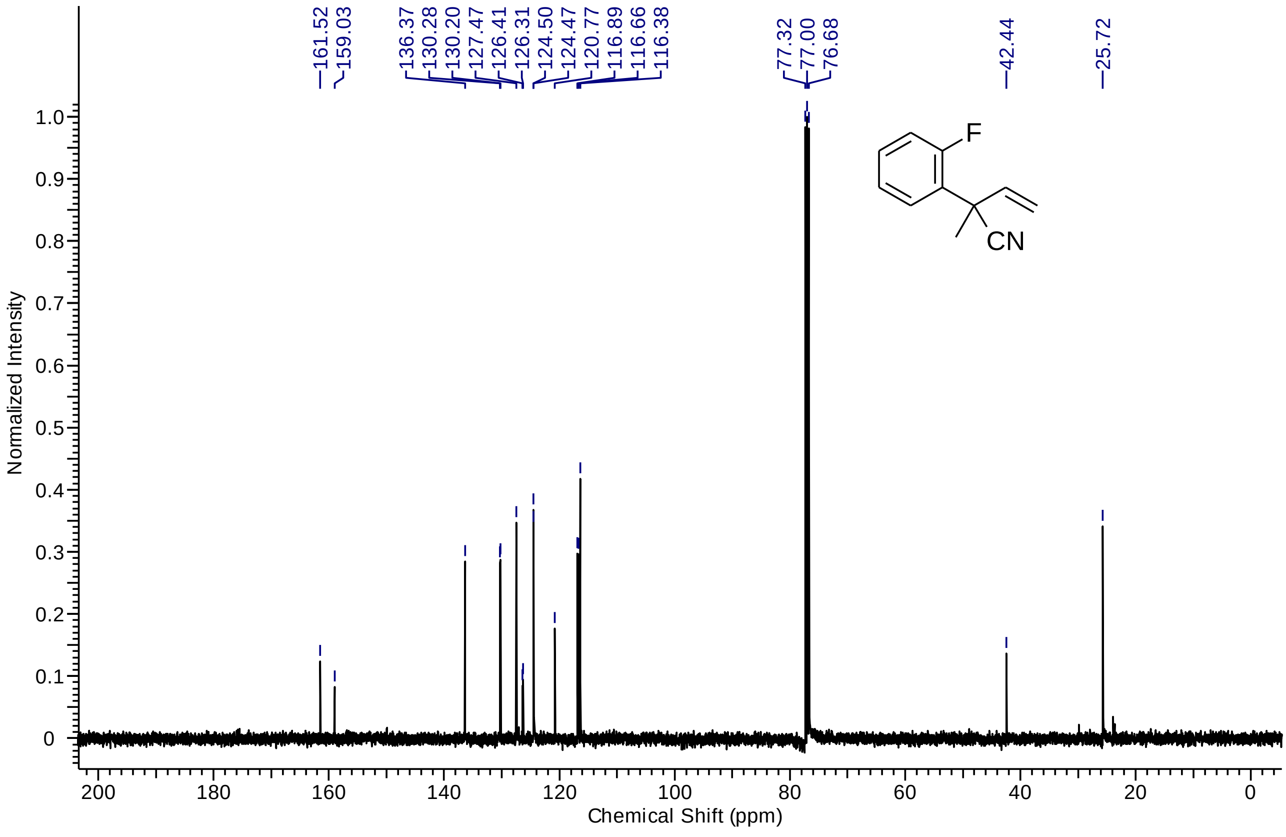

^{13}C NMR spectrum of the compound **3k** in $CDCl_3$, 100 MHz

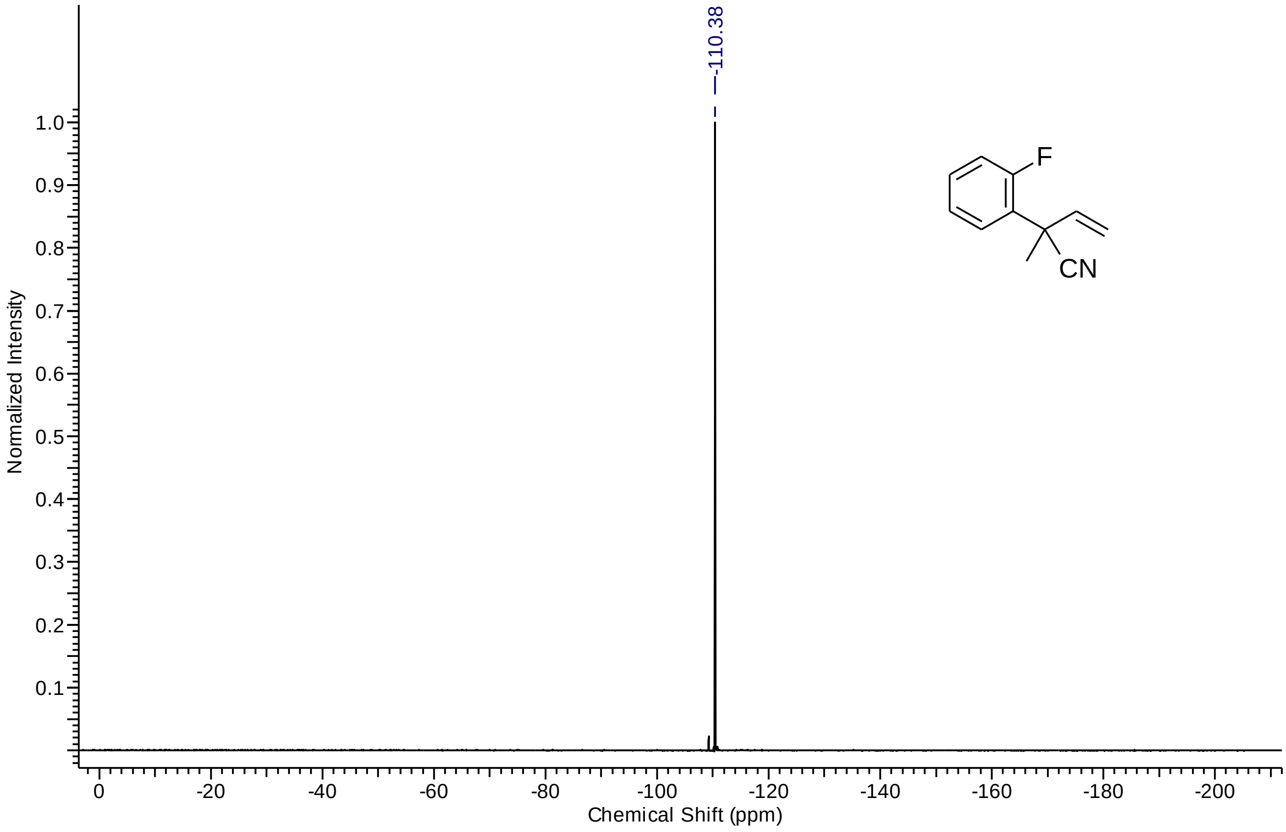

^{19}F NMR spectrum of the compound **3k** in $CDCl_3$, 376 MHz

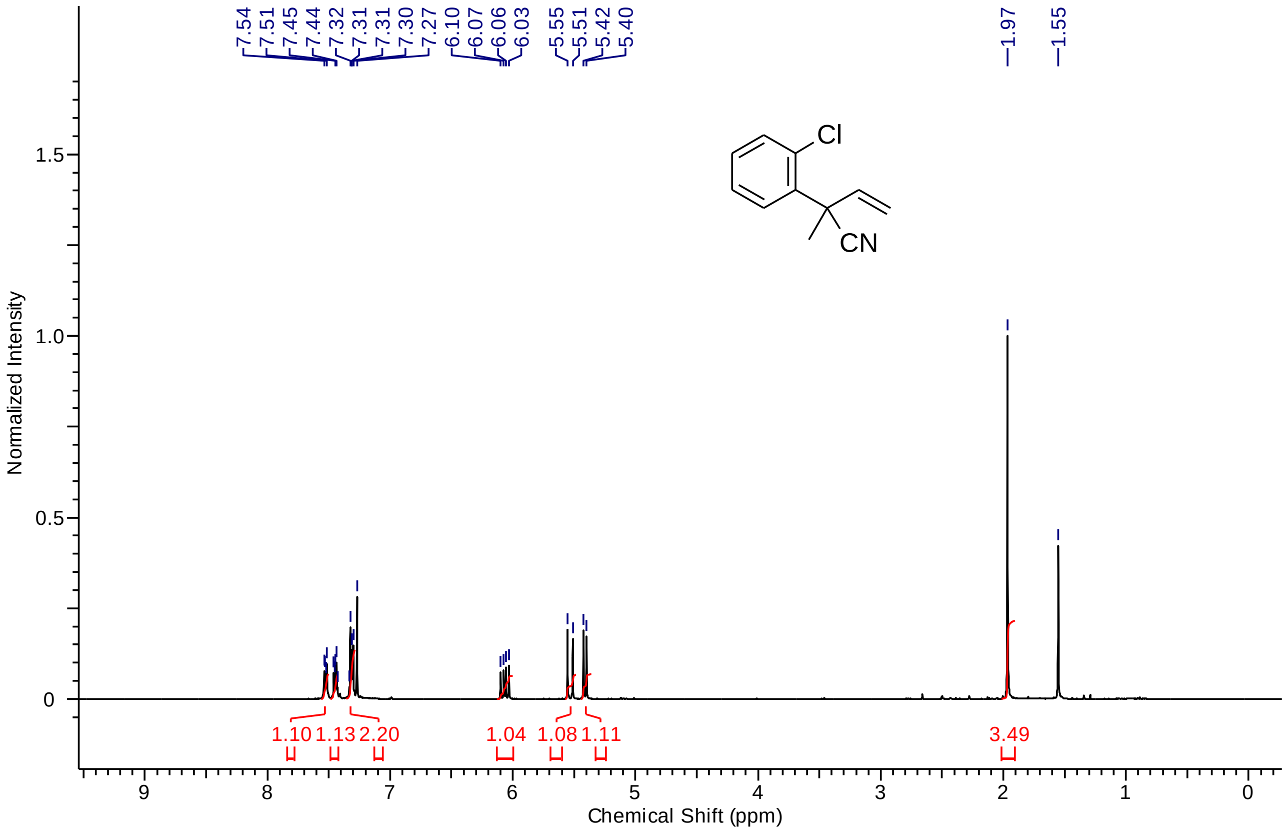

^{1}H NMR spectrum of the compound **3l** in $CDCl_3$, 400 MHz

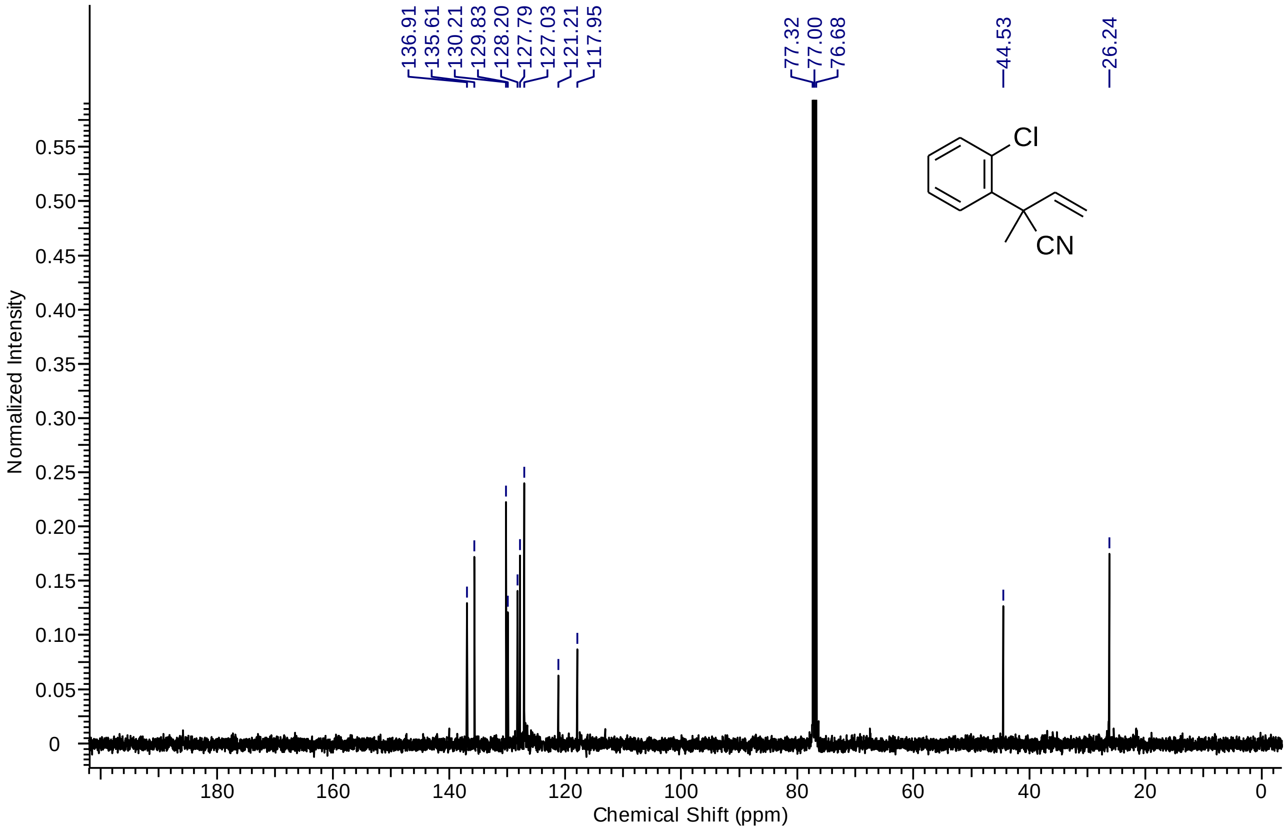

^{13}C NMR spectrum of the compound **3l** in $CDCl_3$, 100 MHz

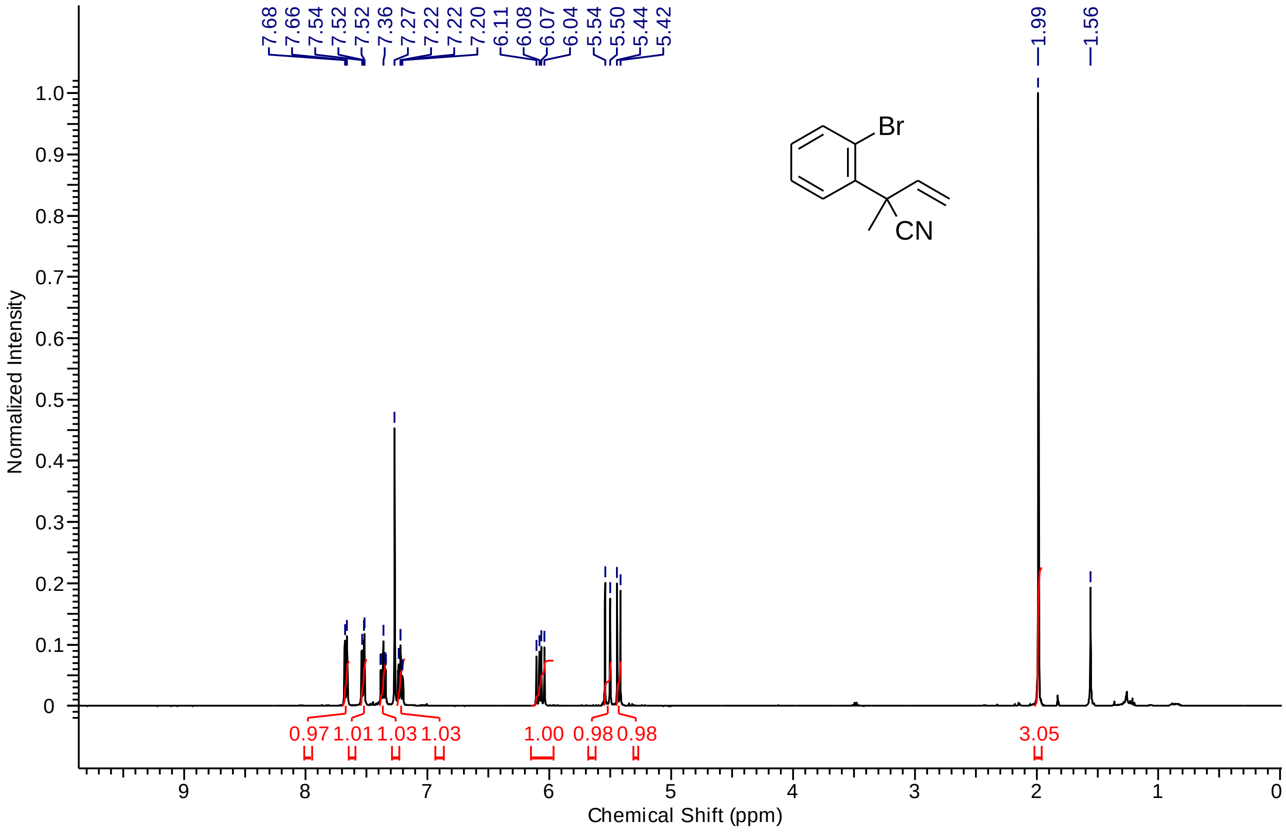

^{1}H NMR spectrum of the compound **3m** in $CDCl_3$, 400 MHz

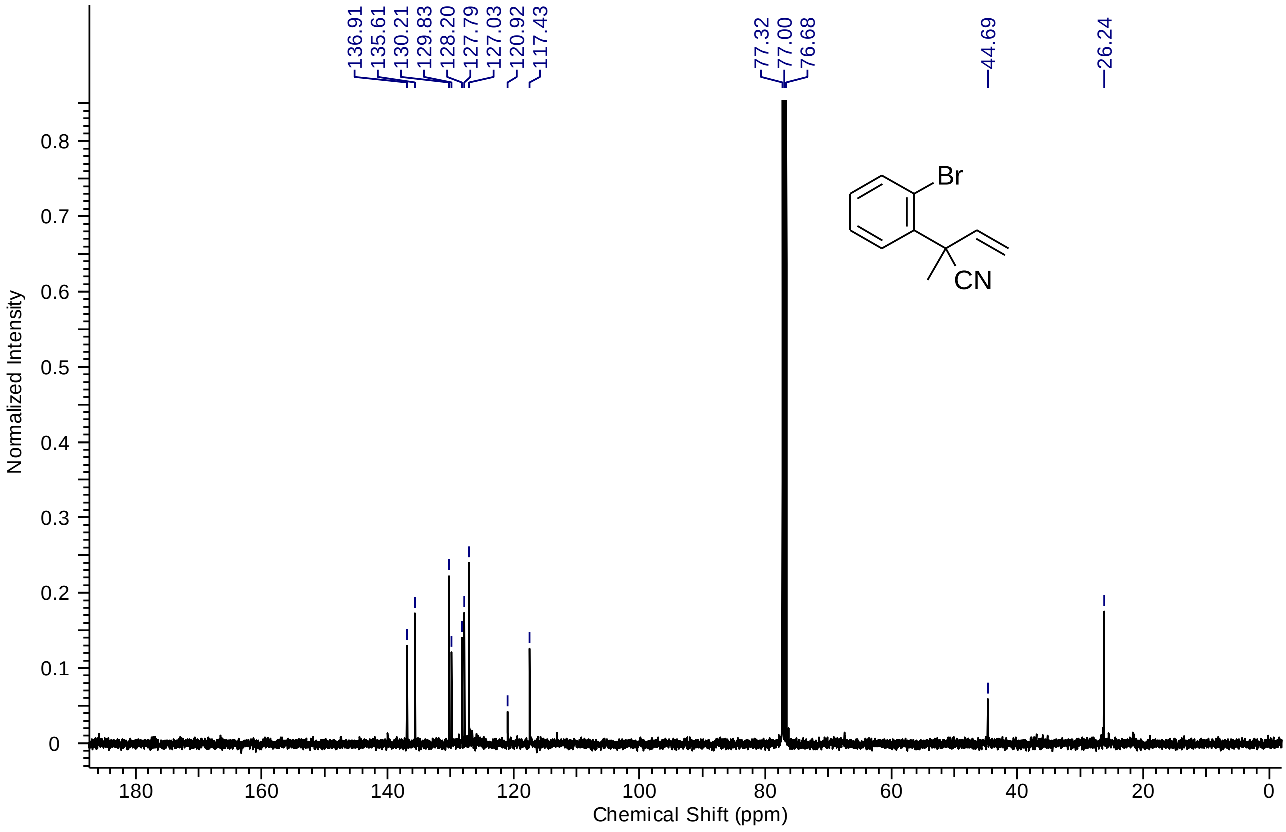

^{13}C NMR spectrum of the compound **3m** in $CDCl_3$, 100 MHz

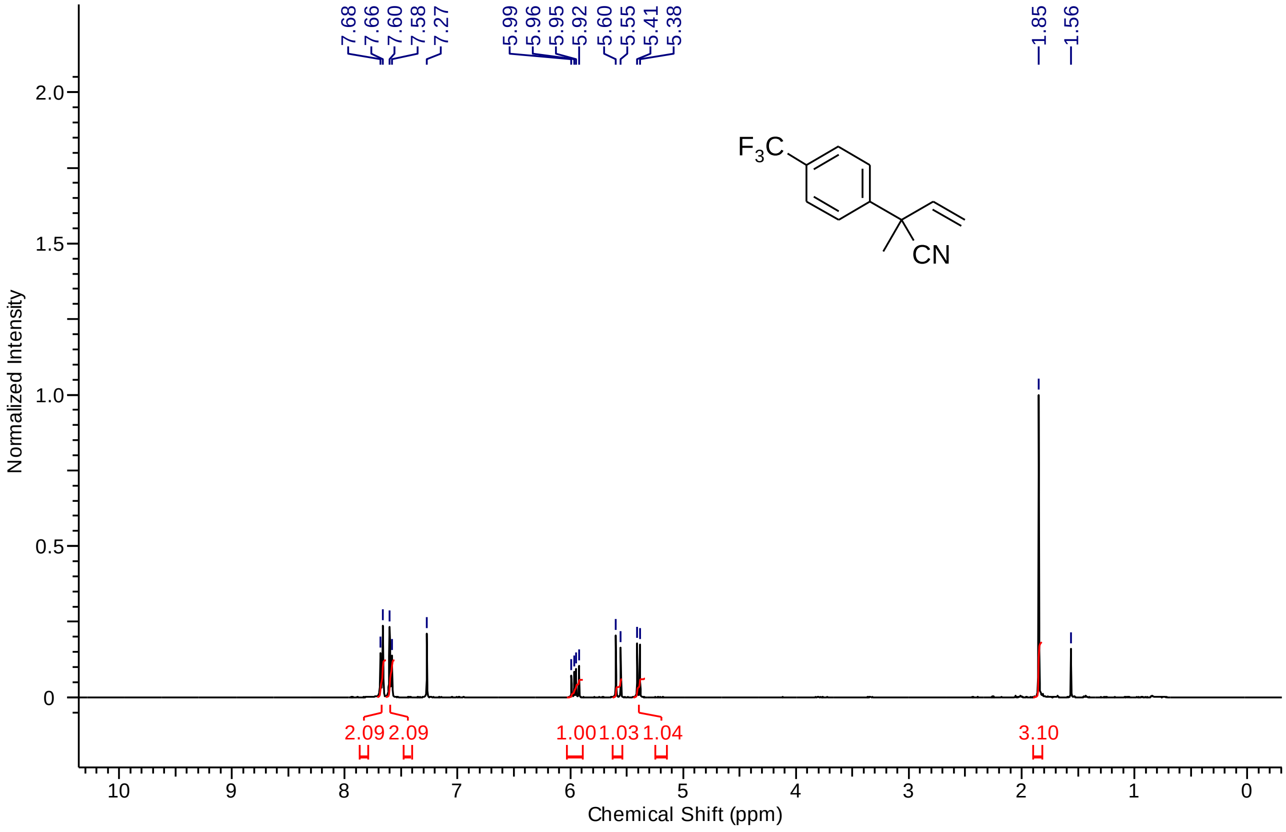

^{1}H NMR spectrum of the compound **3n** in $CDCl_3$, 400 MHz

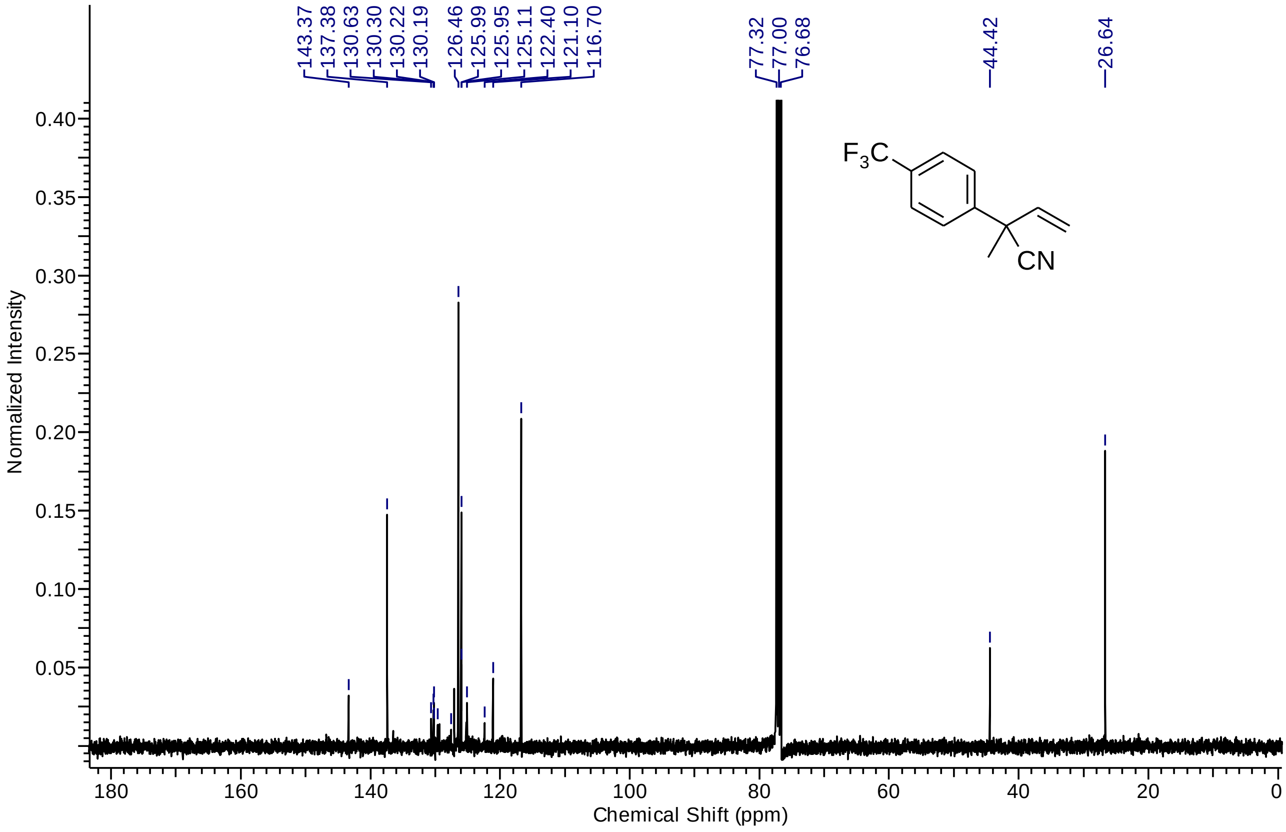

^{13}C NMR spectrum of the compound **3n** in $CDCl_3$, 100 MHz

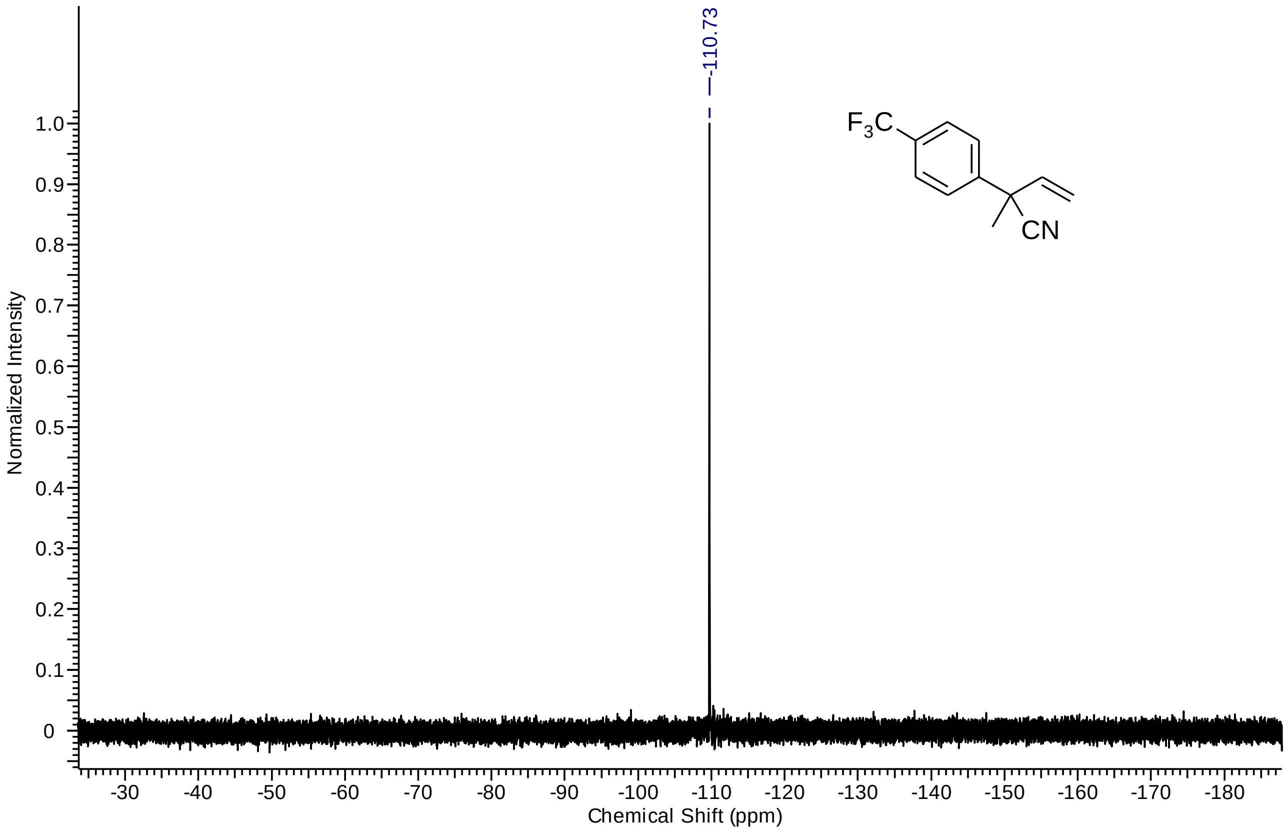

^{19}F NMR spectrum of the compound **3n** in $CDCl_3$, 376 MHz

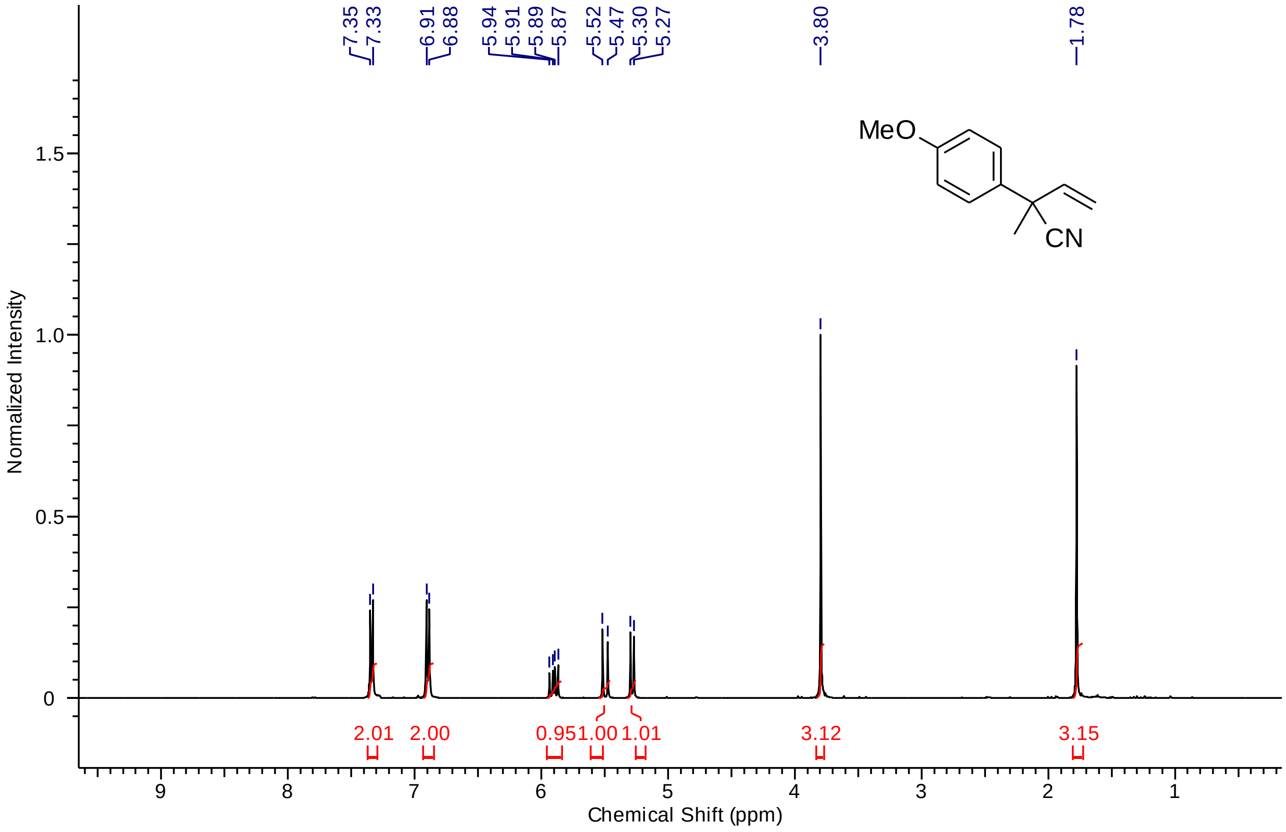

^{1}H NMR spectrum of the compound **3o** in $CDCl_3$, 400 MHz

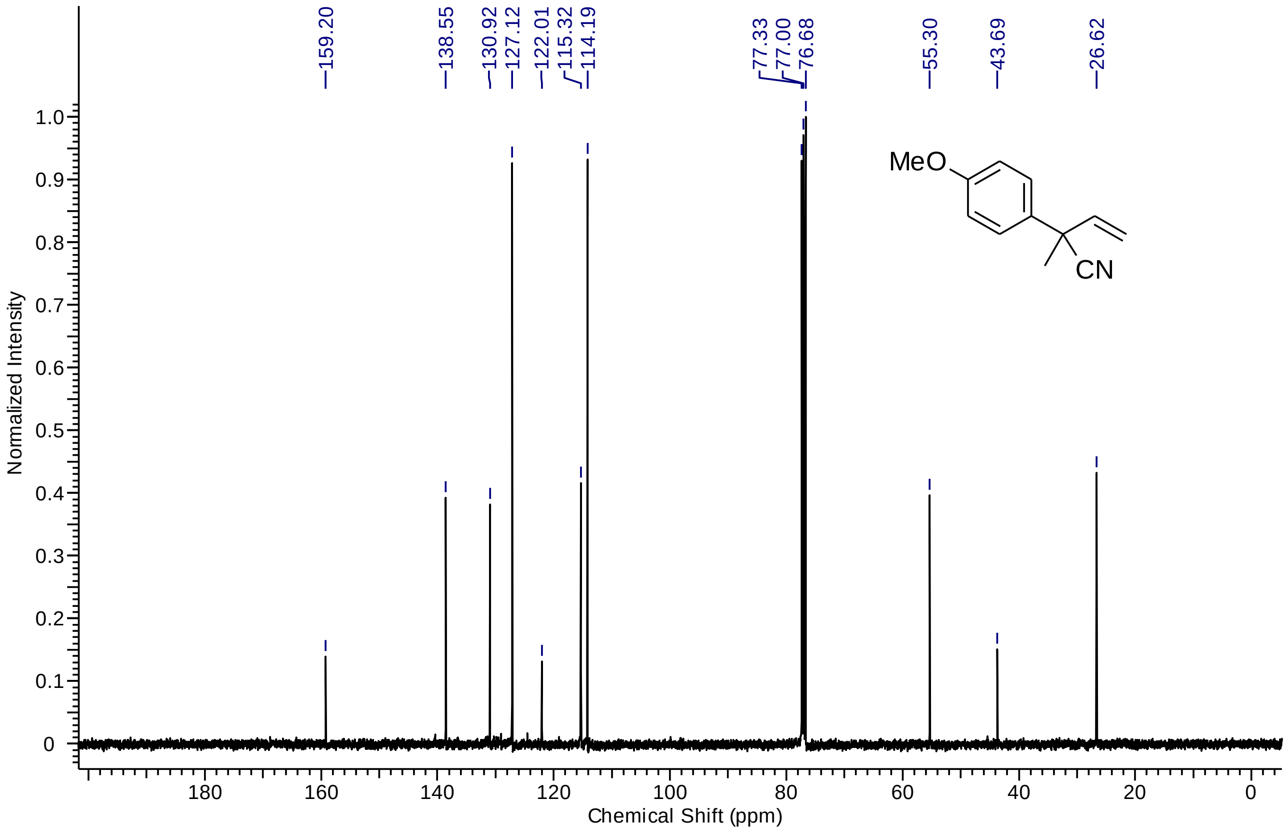

^{13}C NMR spectrum of the compound **3o** in $CDCl_3$, 100 MHz

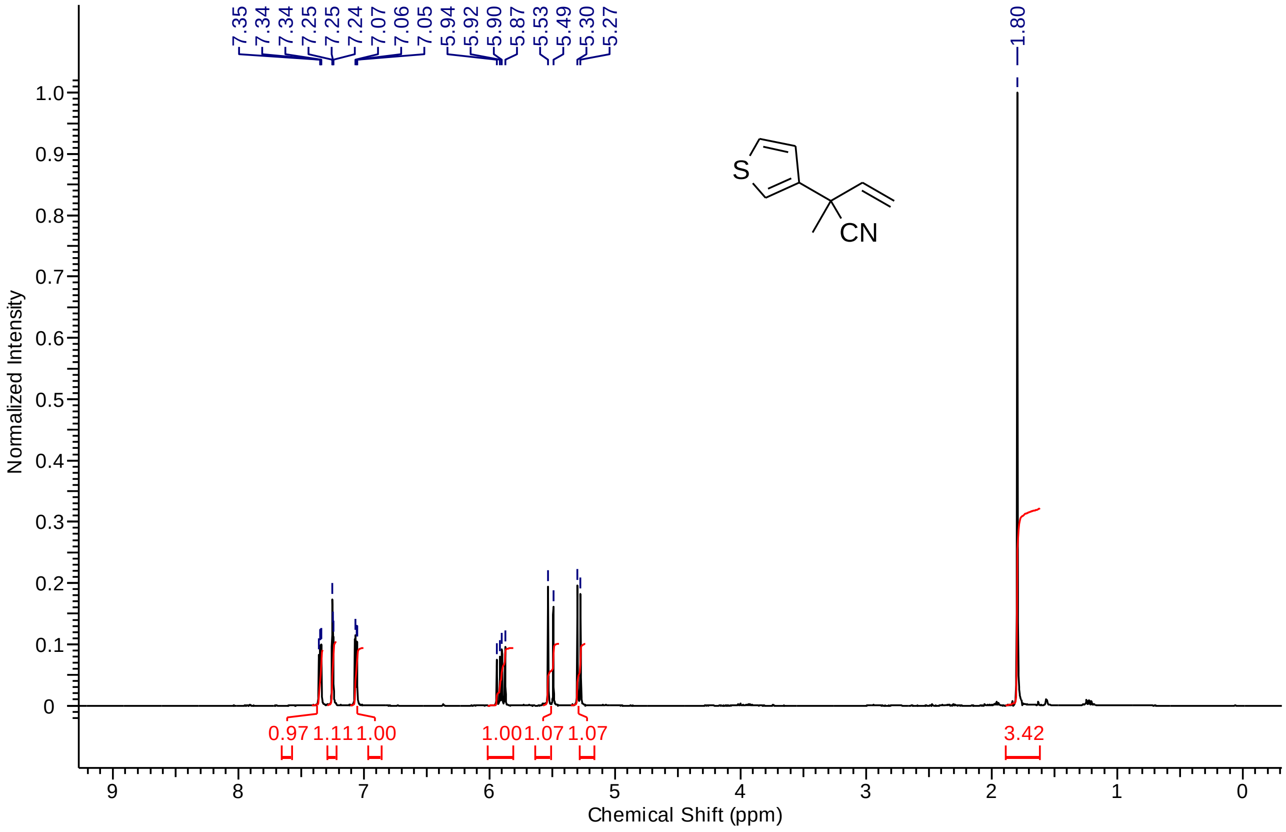

^{1}H NMR spectrum of the compound **3p** in $CDCl_3$, 400 MHz

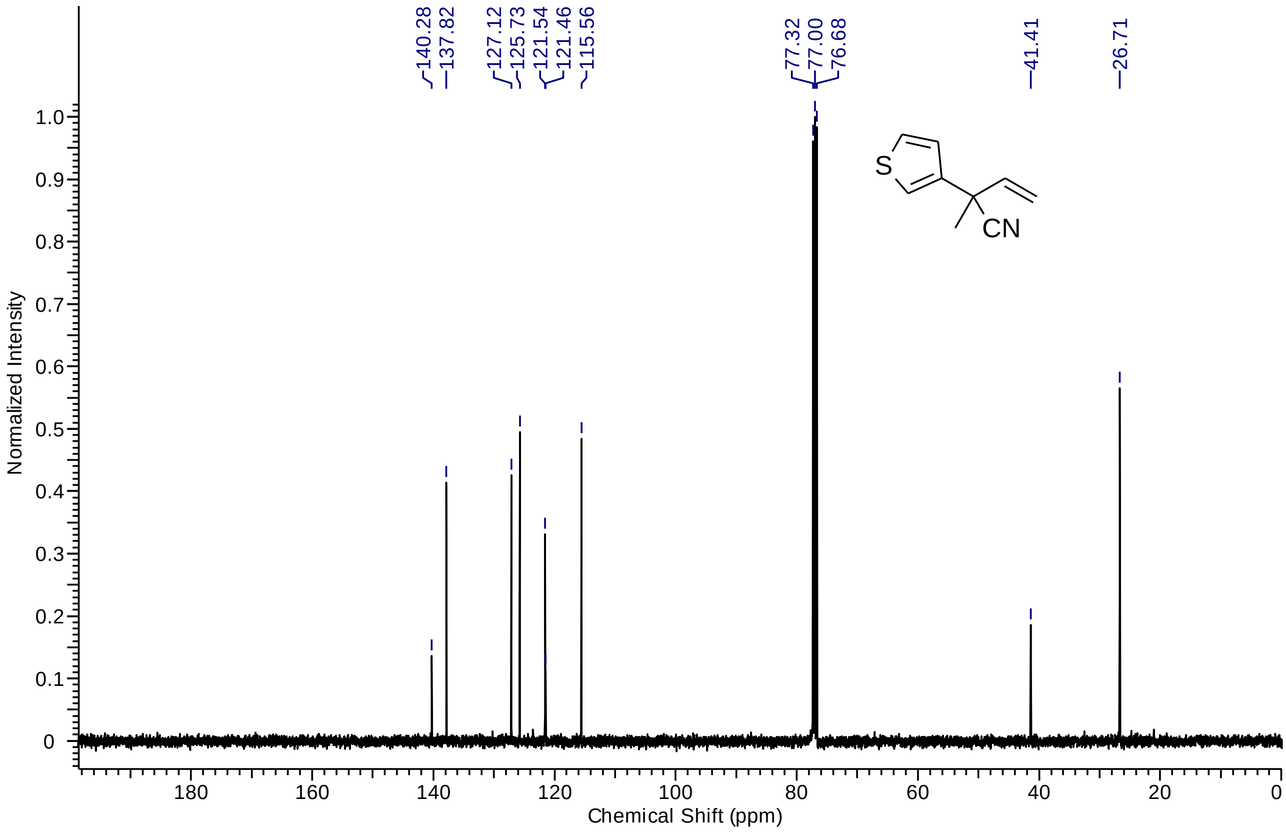

^{13}C NMR spectrum of the compound **3p** in $CDCl_3$, 100 MHz

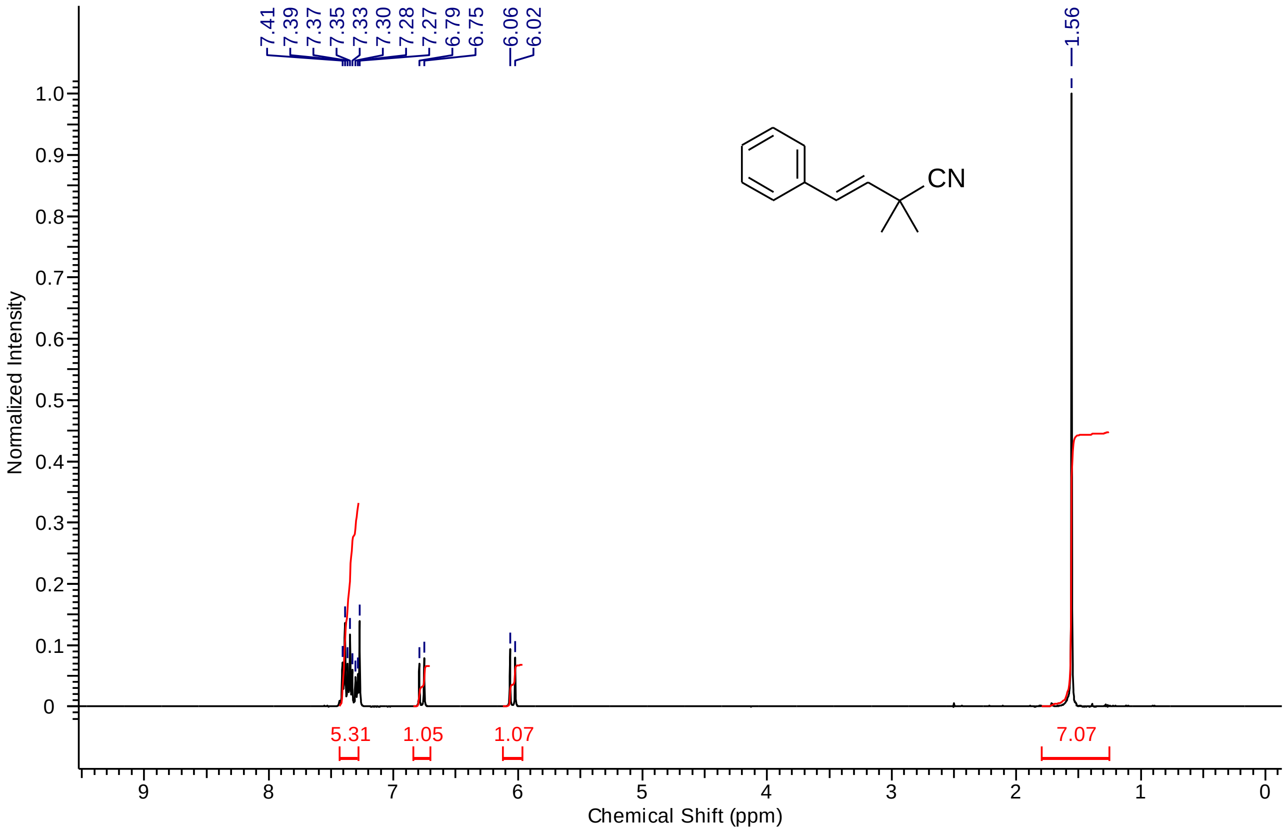

^{1}H NMR spectrum of the compound **3q** in $CDCl_3$, 400 MHz

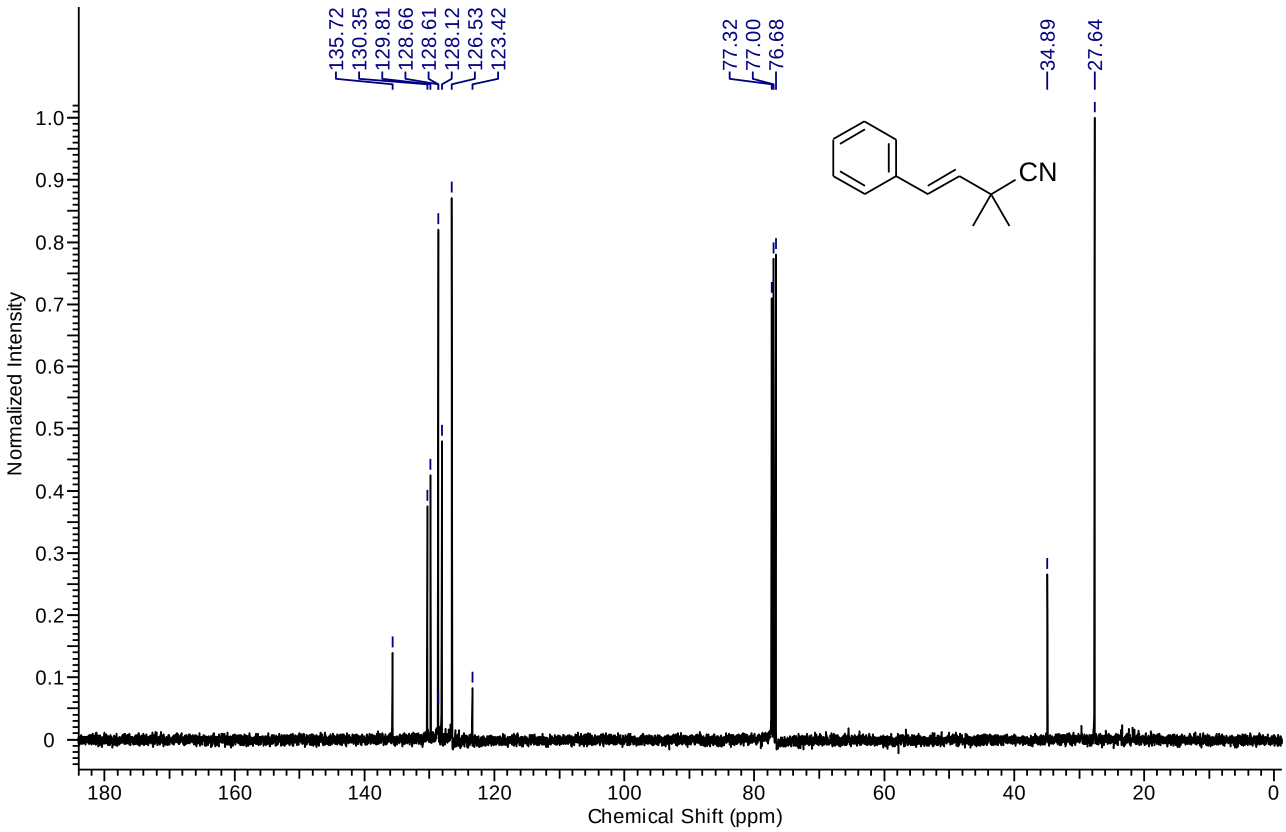

^{13}C NMR spectrum of the compound **3q** in $CDCl_3$, 100 MHz

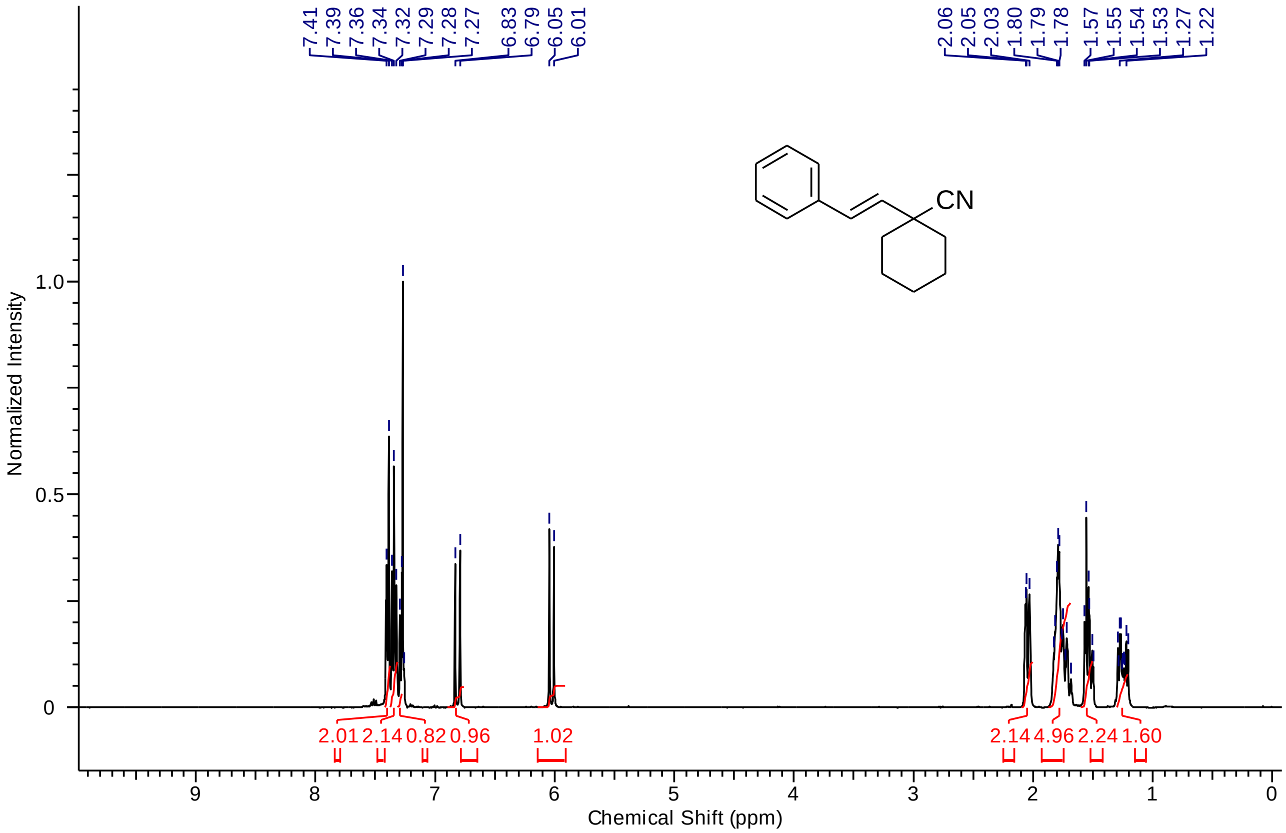

^{1}H NMR spectrum of the compound **3r** in $CDCl_3$, 400 MHz

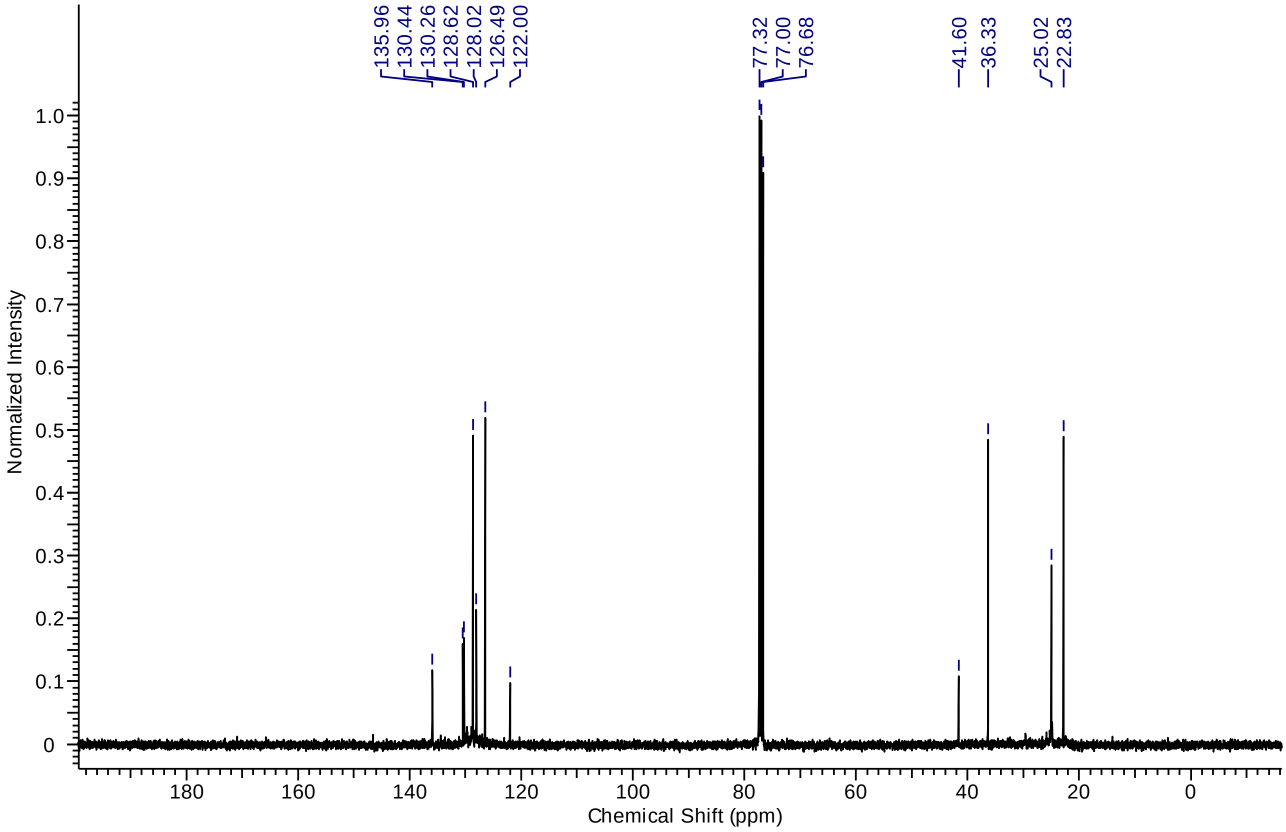

^{13}C NMR spectrum of the compound **3r** in $CDCl_3$, 100 MHz

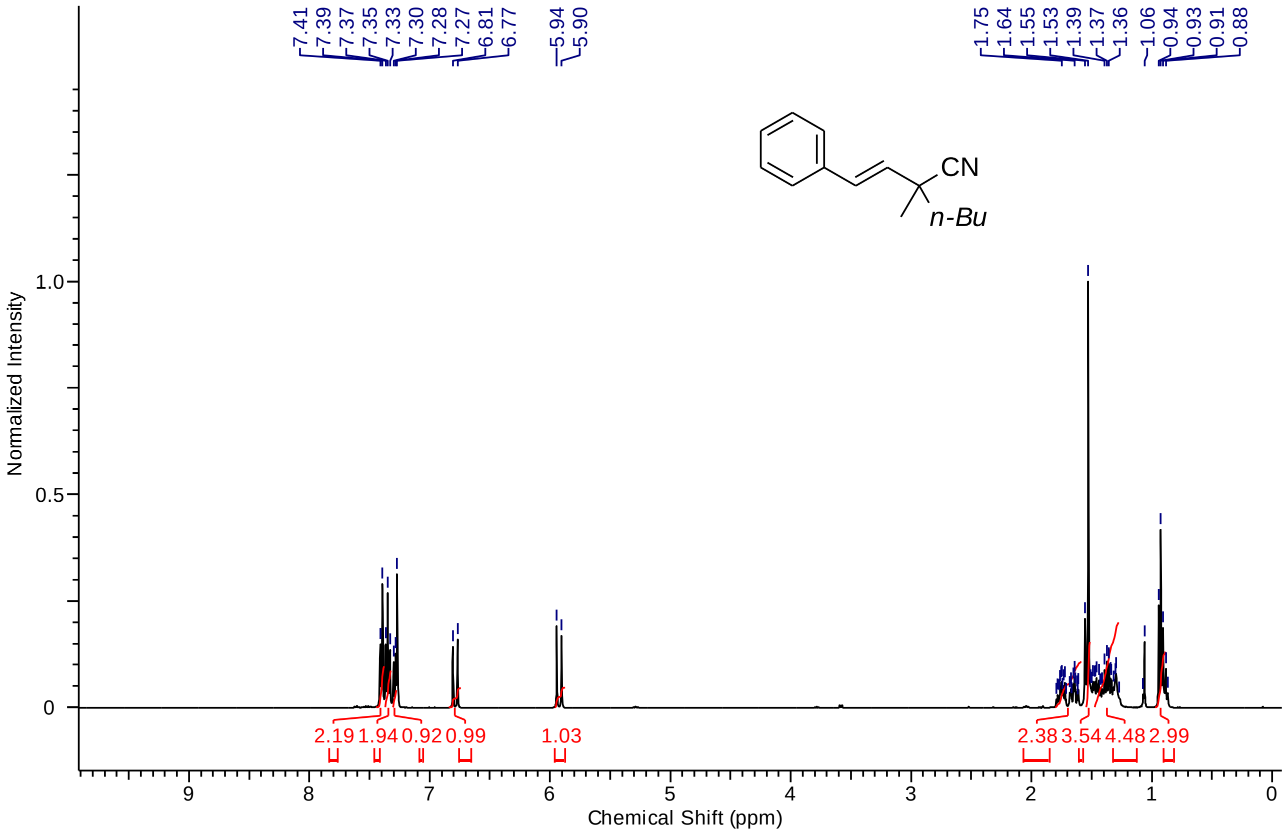

^{1}H NMR spectrum of the compound **3s** in $CDCl_3$, 400 MHz

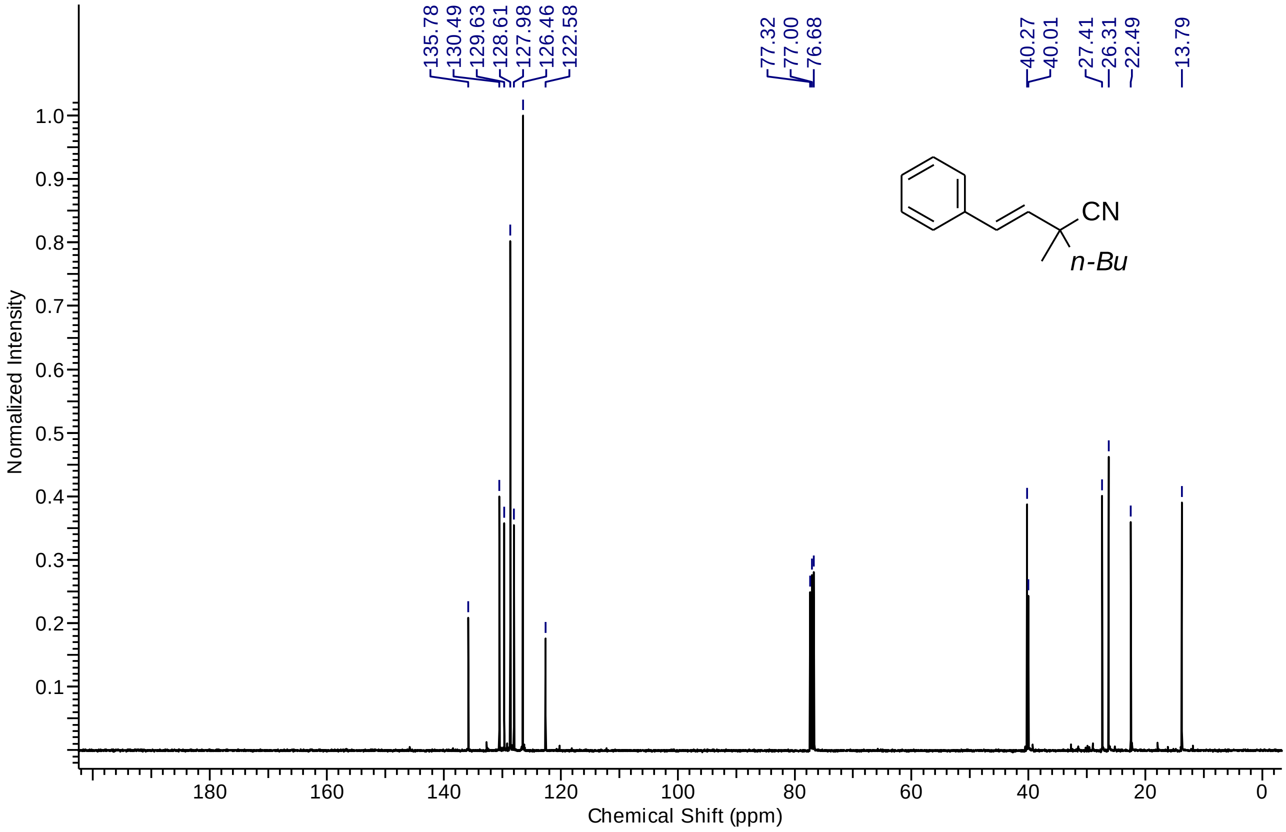

^{13}C NMR spectrum of the compound **3s** in $CDCl_3$, 100 MHz

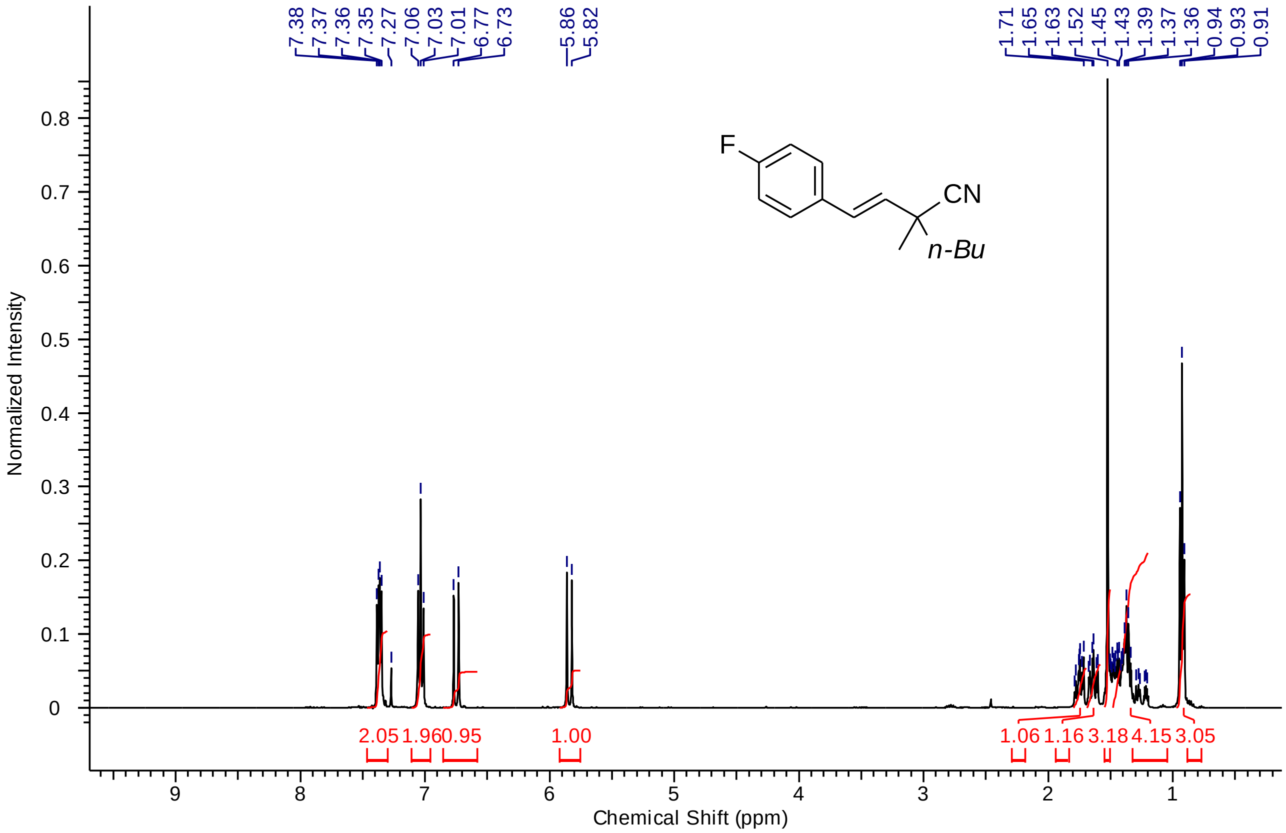

^{1}H NMR spectrum of the compound **3t** in $CDCl_3$, 400 MHz

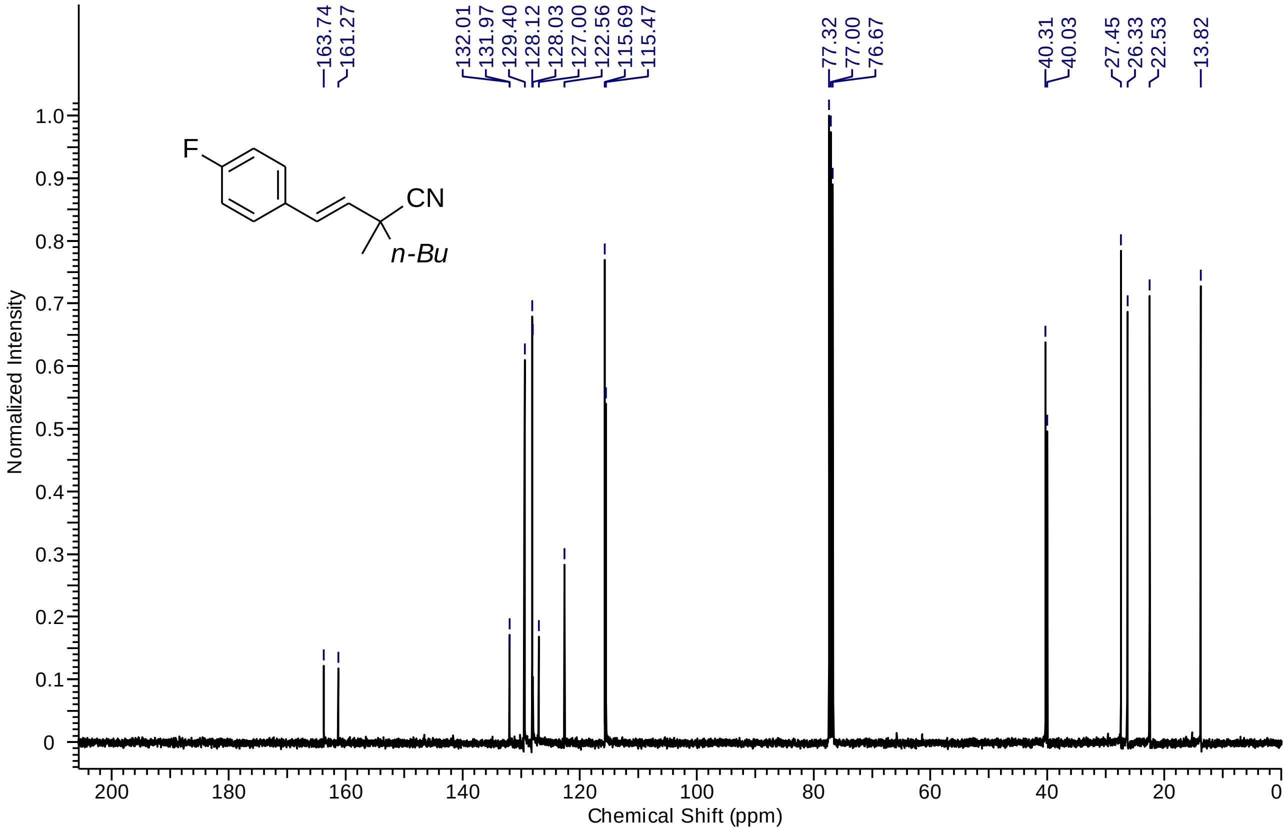

^{13}C NMR spectrum of the compound **3t** in $CDCl_3$, 100 MHz

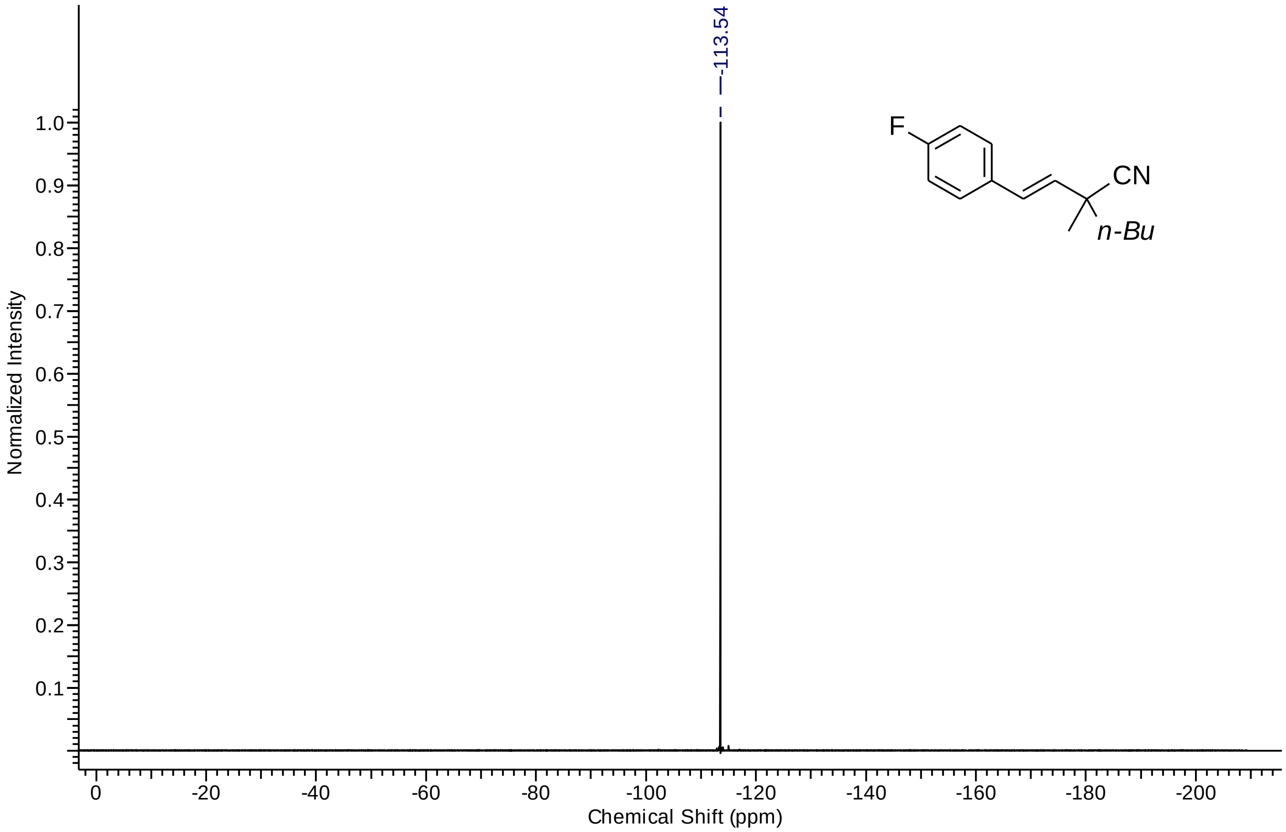

^{19}F NMR spectrum of the compound **3t** in $CDCl_3$, 376 MHz

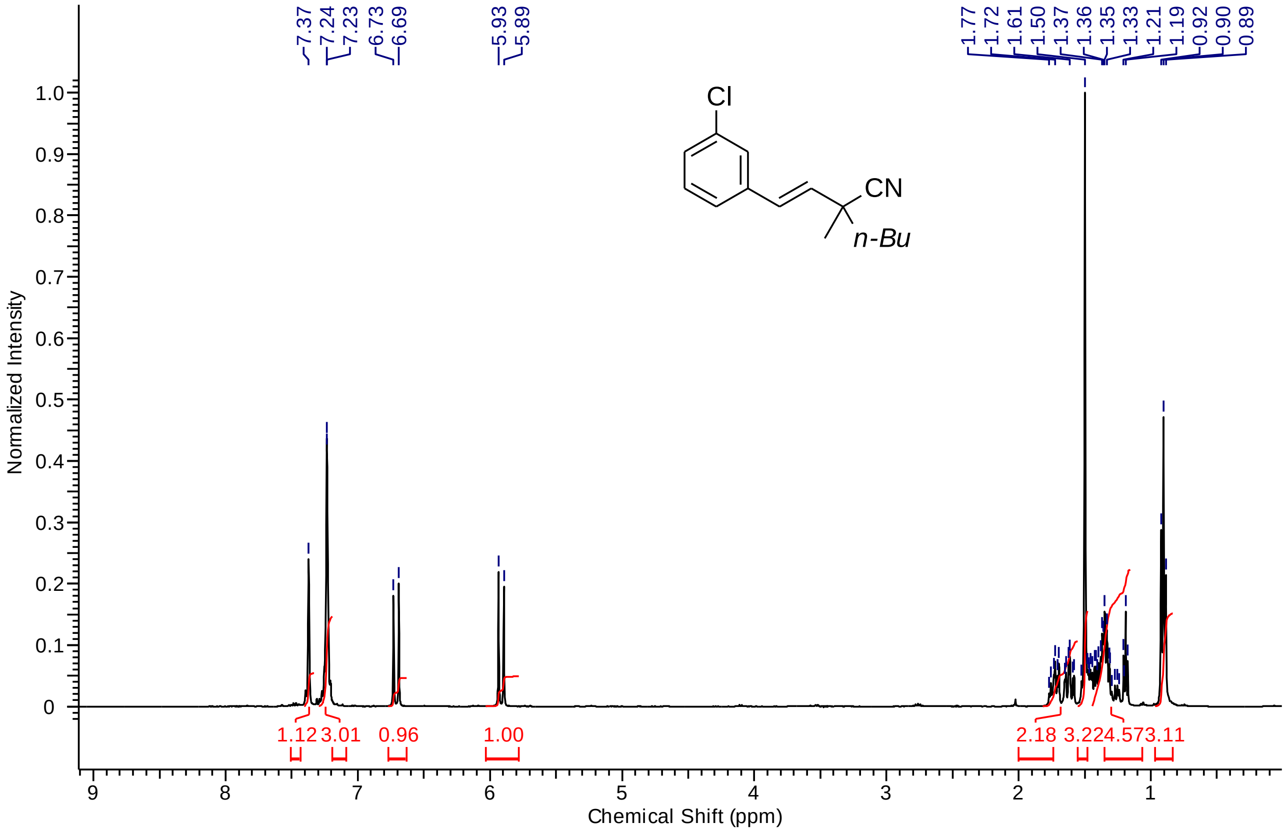

^{1}H NMR spectrum of the compound **3u** in $CDCl_3$, 400 MHz

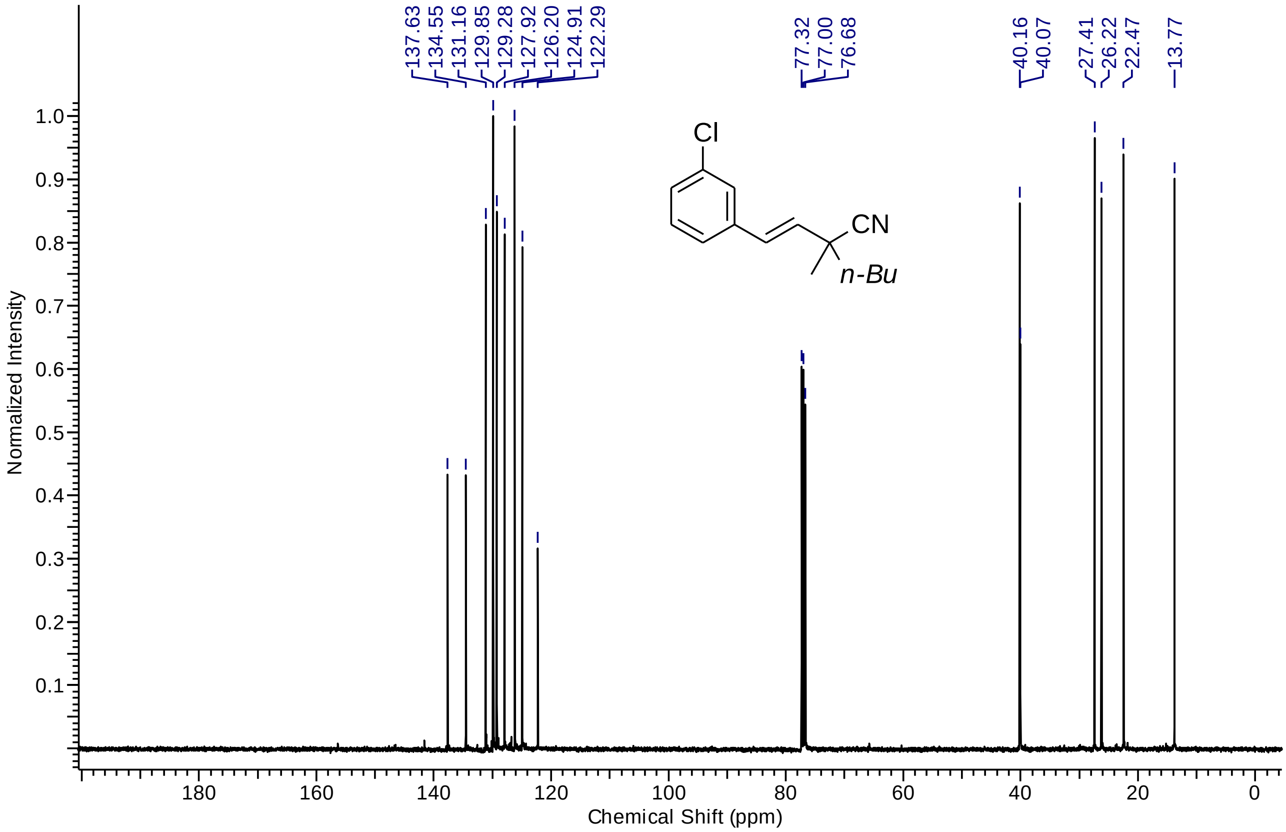

^{13}C NMR spectrum of the compound **3u** in $CDCl_3$, 100 MHz

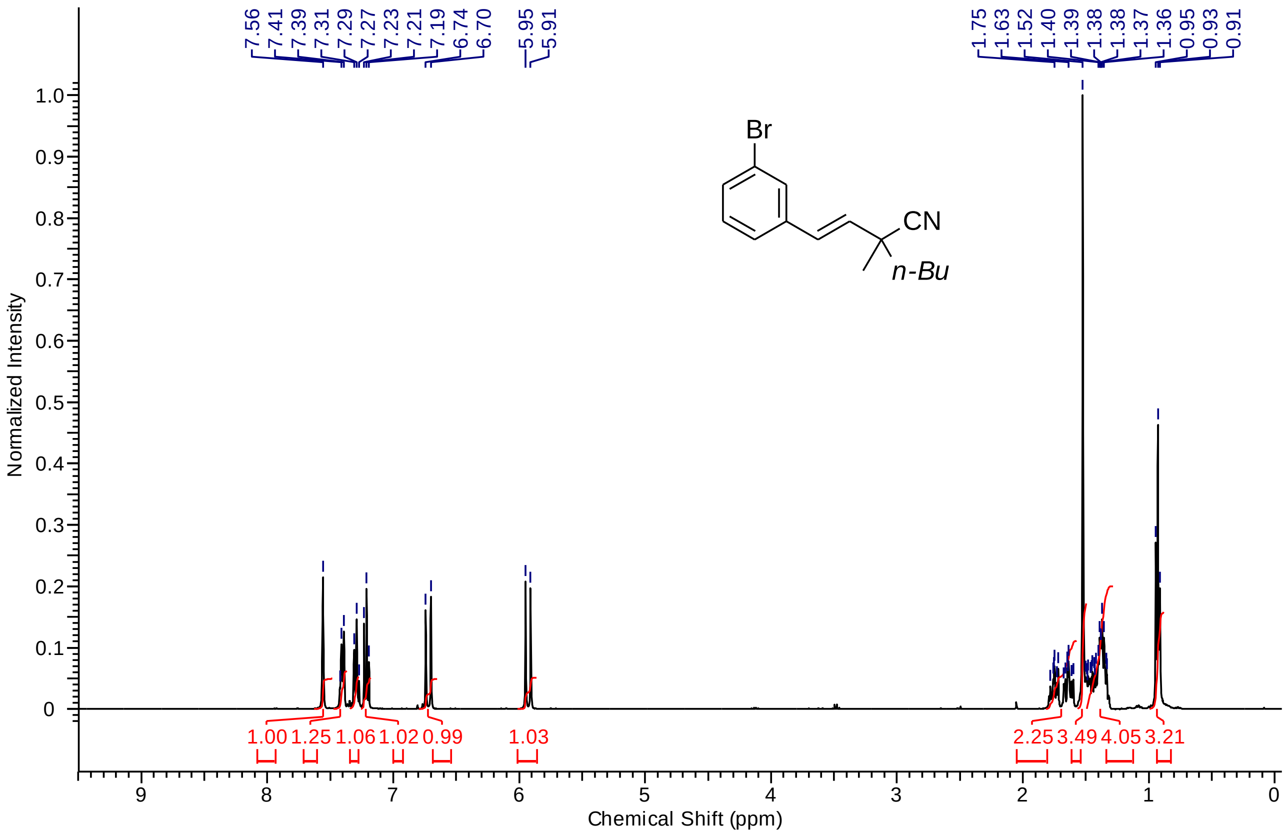

^{1}H NMR spectrum of the compound **3v** in $CDCl_3$, 400 MHz

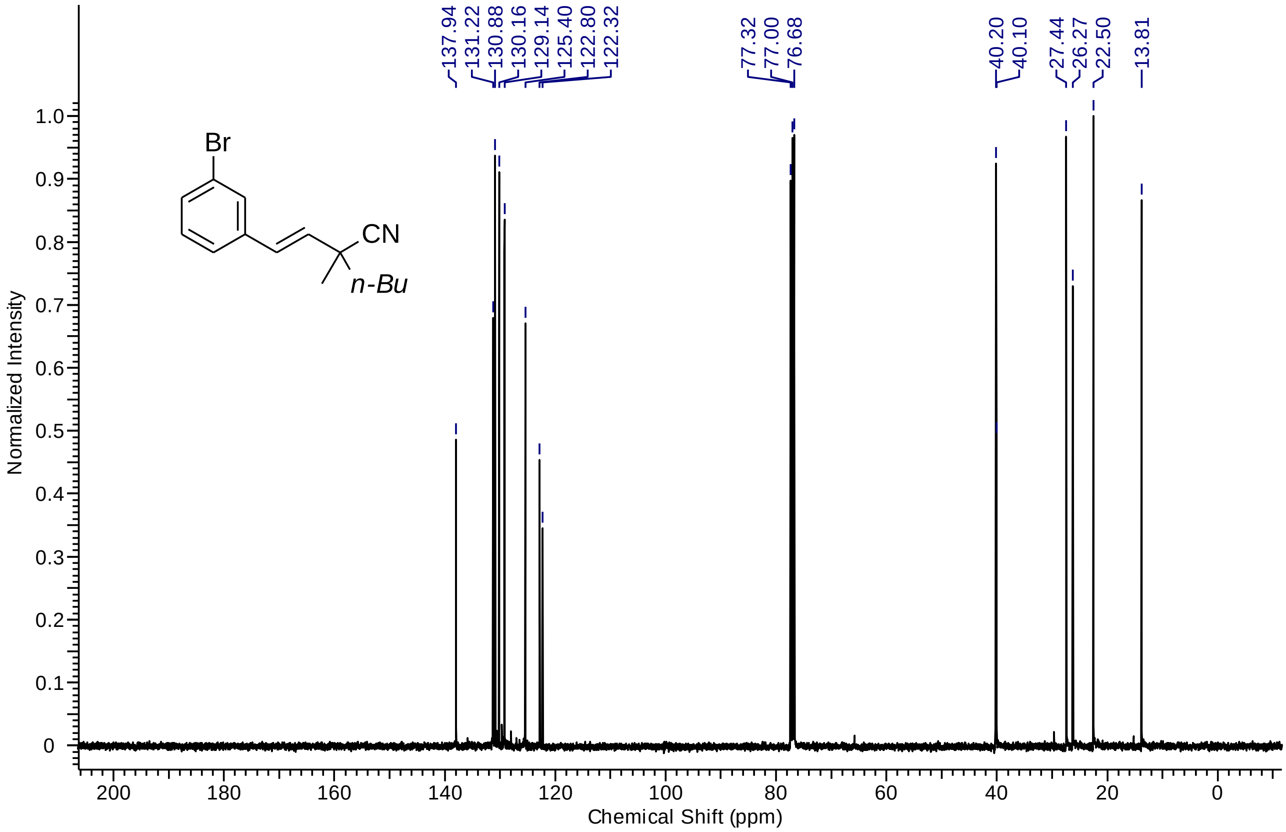

^{13}C NMR spectrum of the compound **3v** in $CDCl_3$, 100 MHz

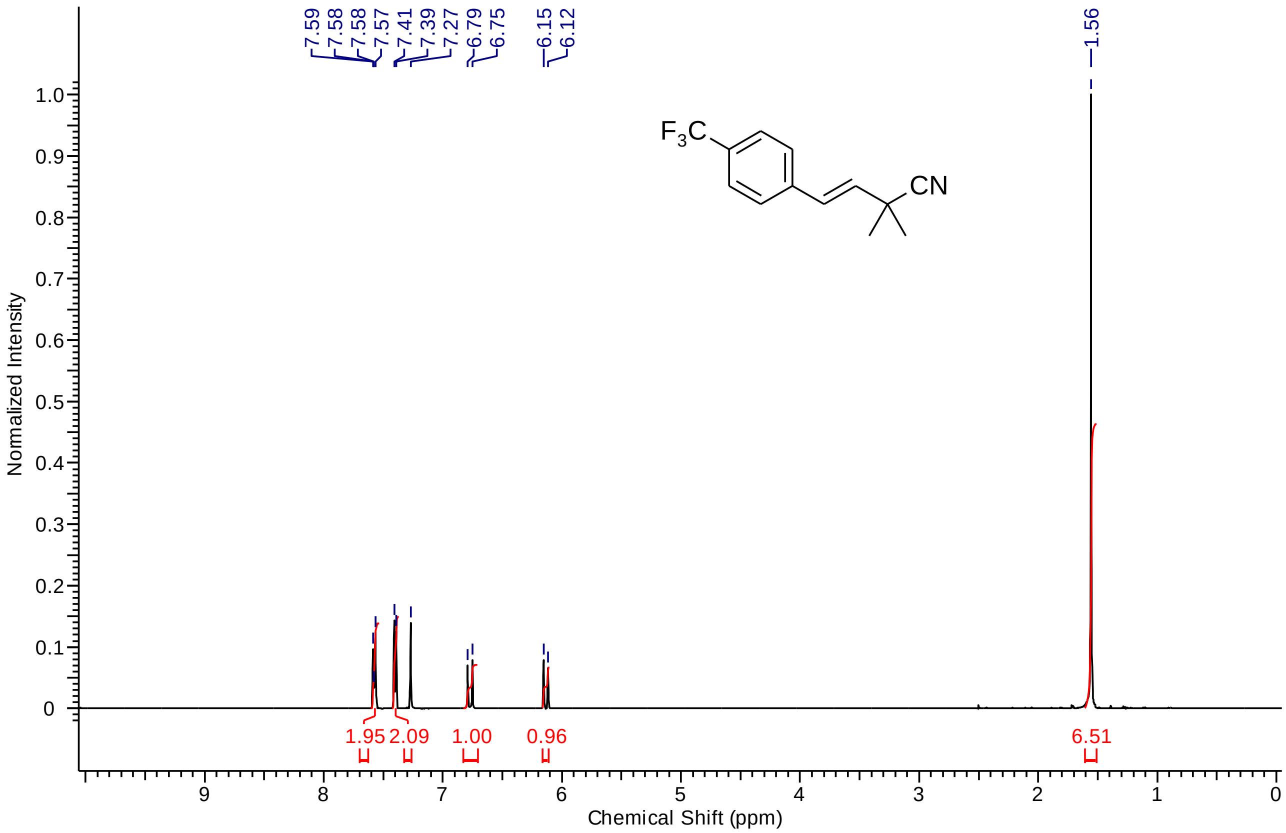

^{1}H NMR spectrum of the compound **3w** in $CDCl_3$, 400 MHz

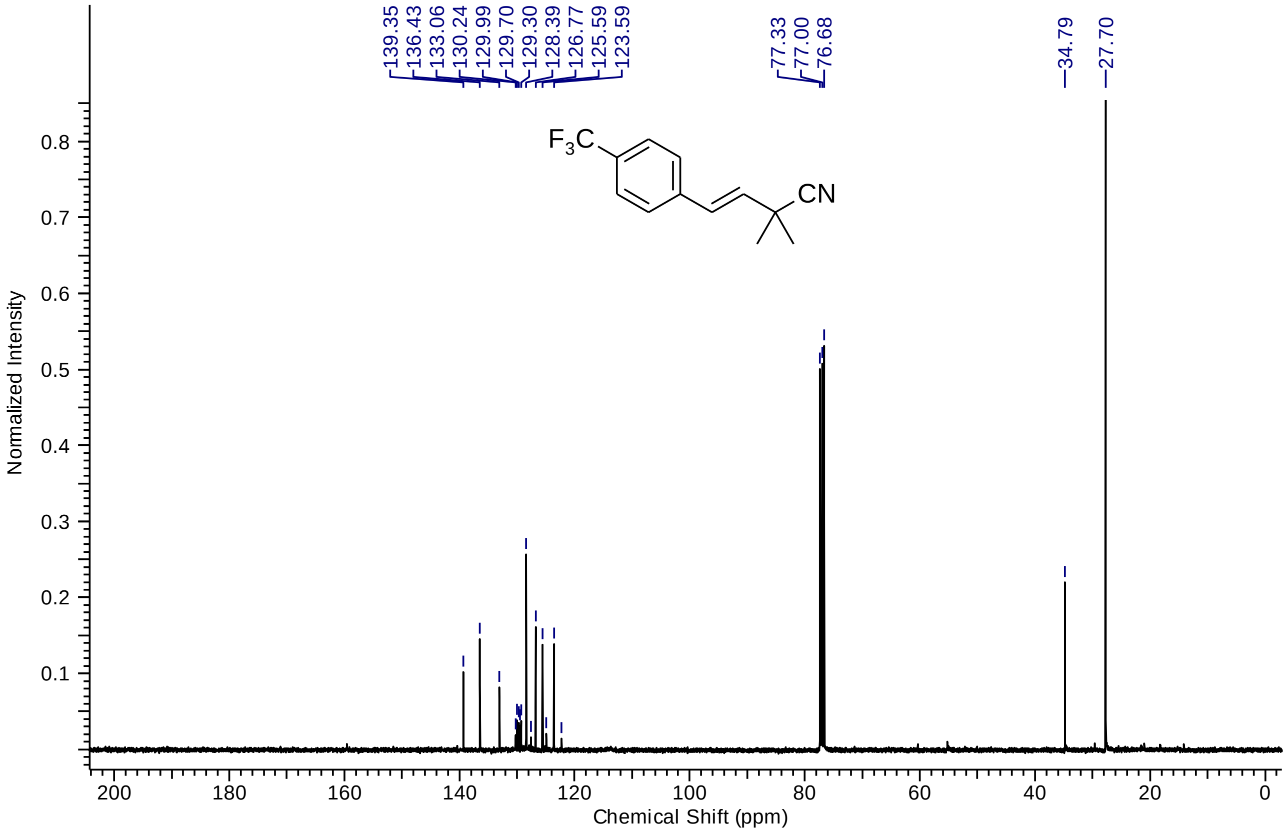

^{13}C NMR spectrum of the compound **3w** in $CDCl_3$, 100 MHz

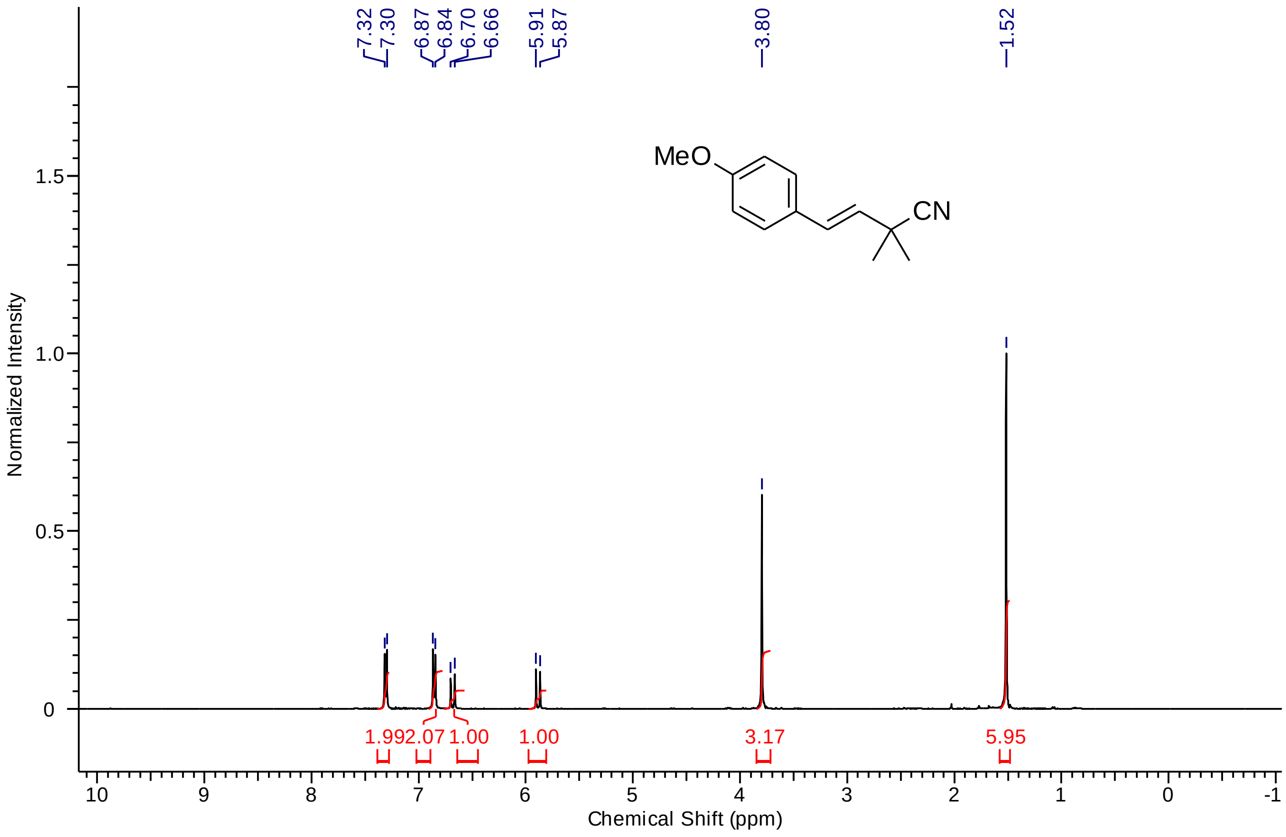

^{1}H NMR spectrum of the compound **3x** in $CDCl_3$, 400 MHz

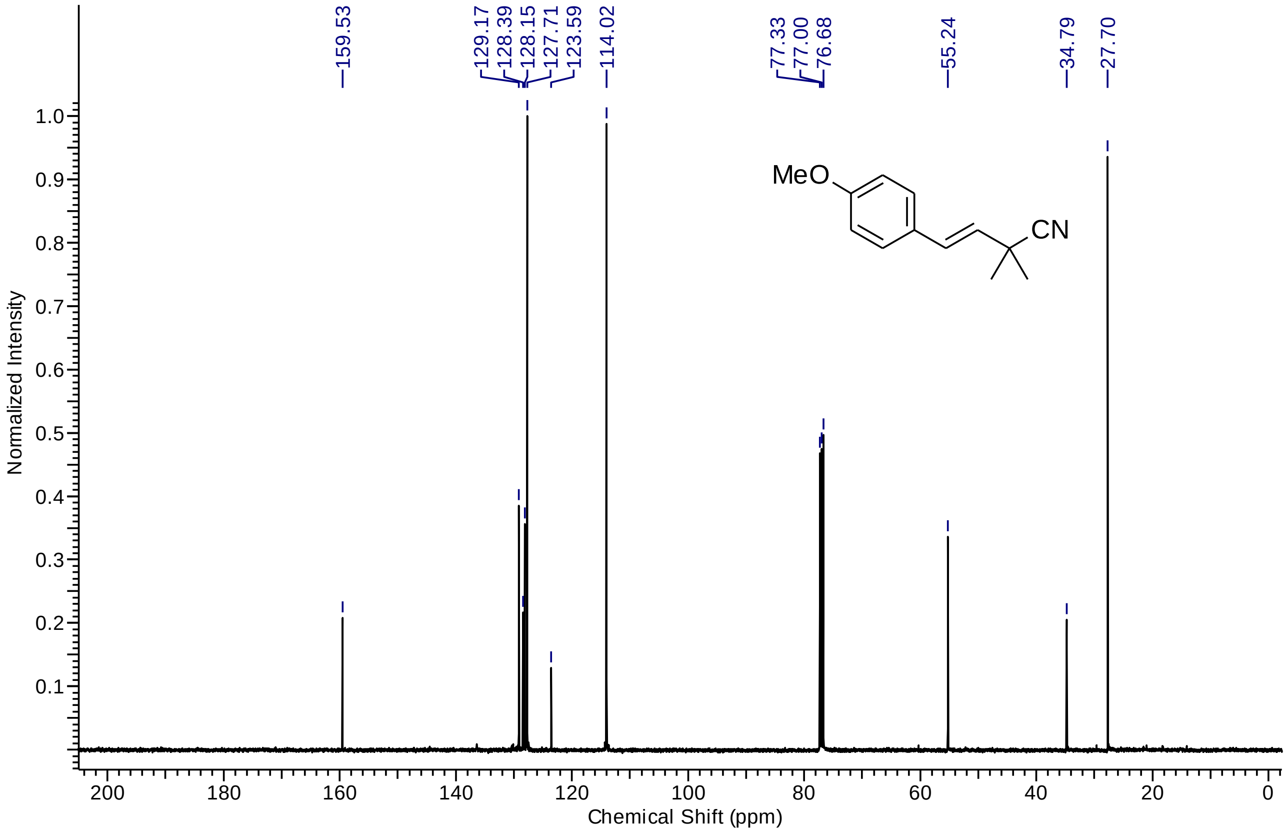

^{13}C NMR spectrum of the compound **3x** in $CDCl_3$, 100 MHz

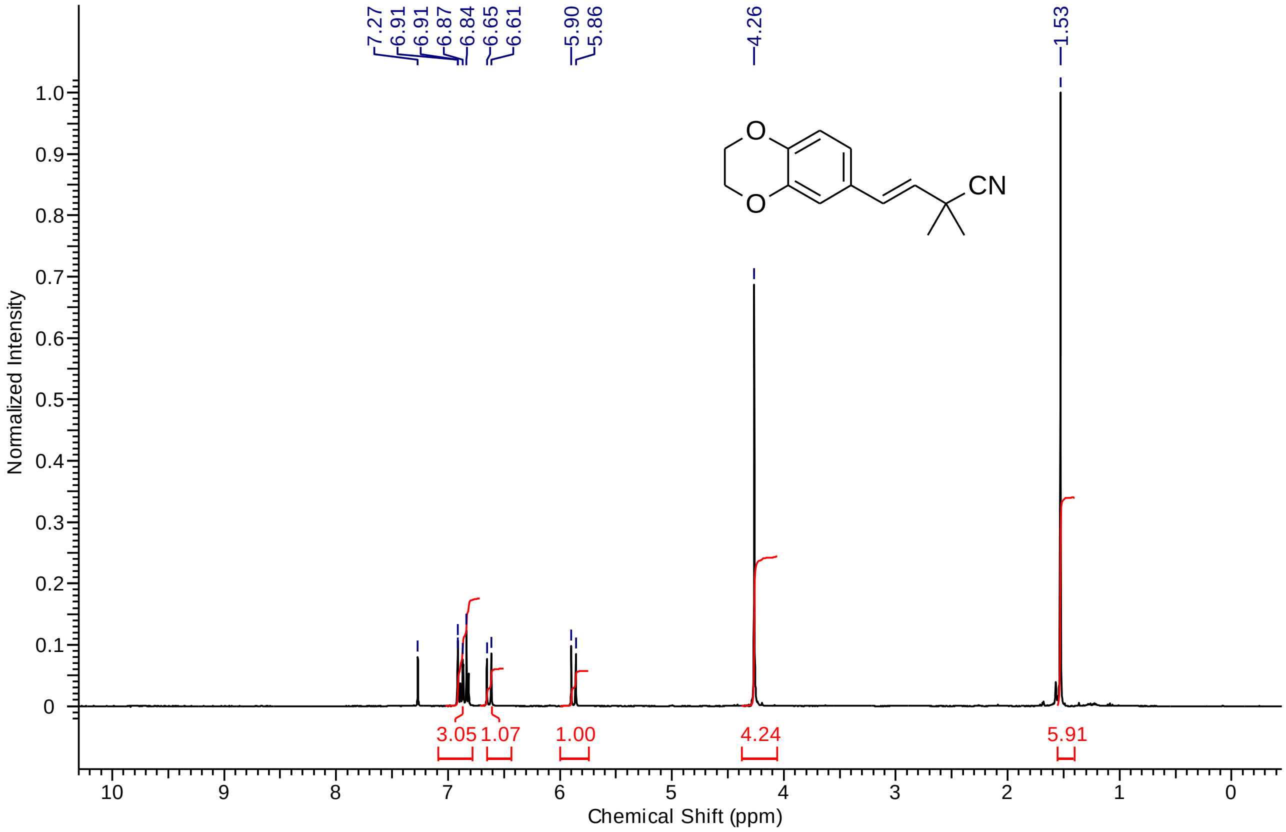

^{1}H NMR spectrum of the compound **3y** in $CDCl_3$, 400 MHz

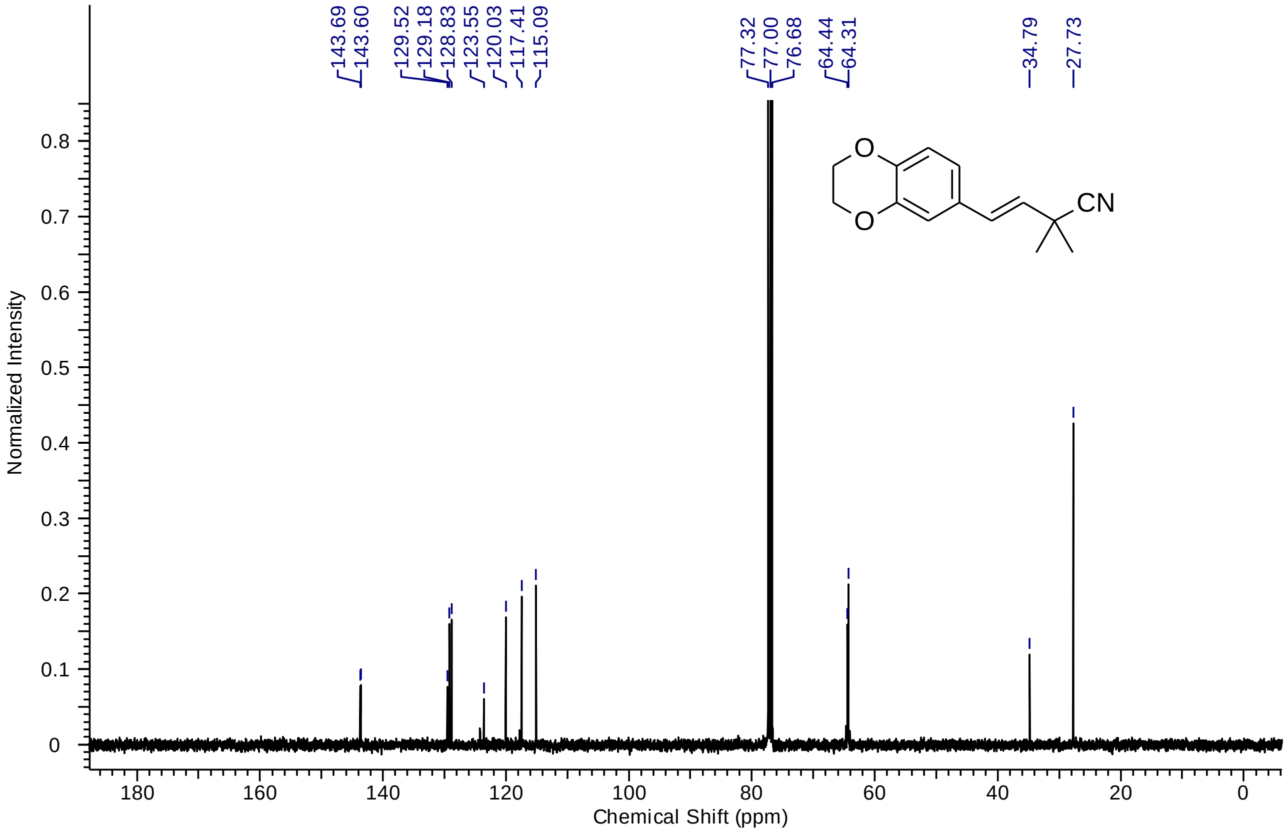

^{13}C NMR spectrum of the compound **3y** in $CDCl_3$, 100 MHz

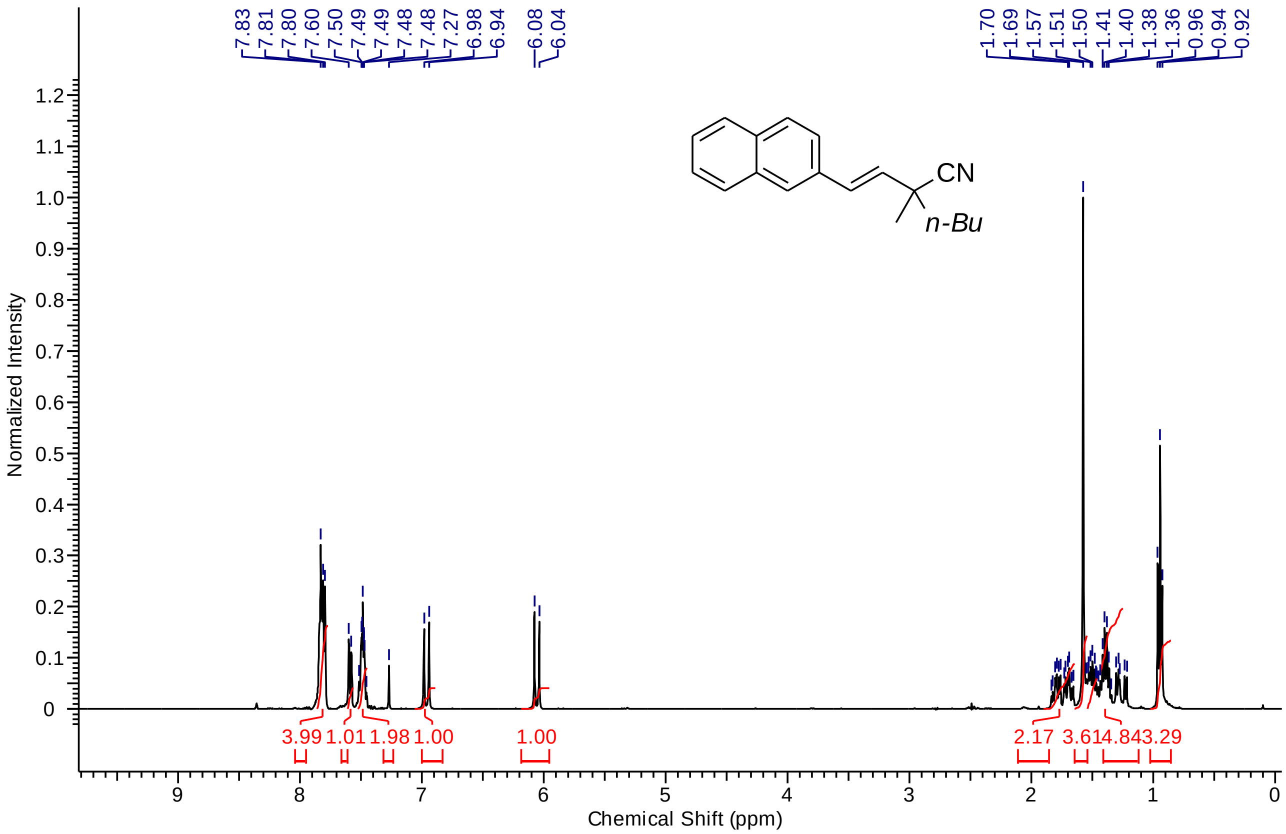

^{1}H NMR spectrum of the compound **3y** in $CDCl_3$, 400 MHz

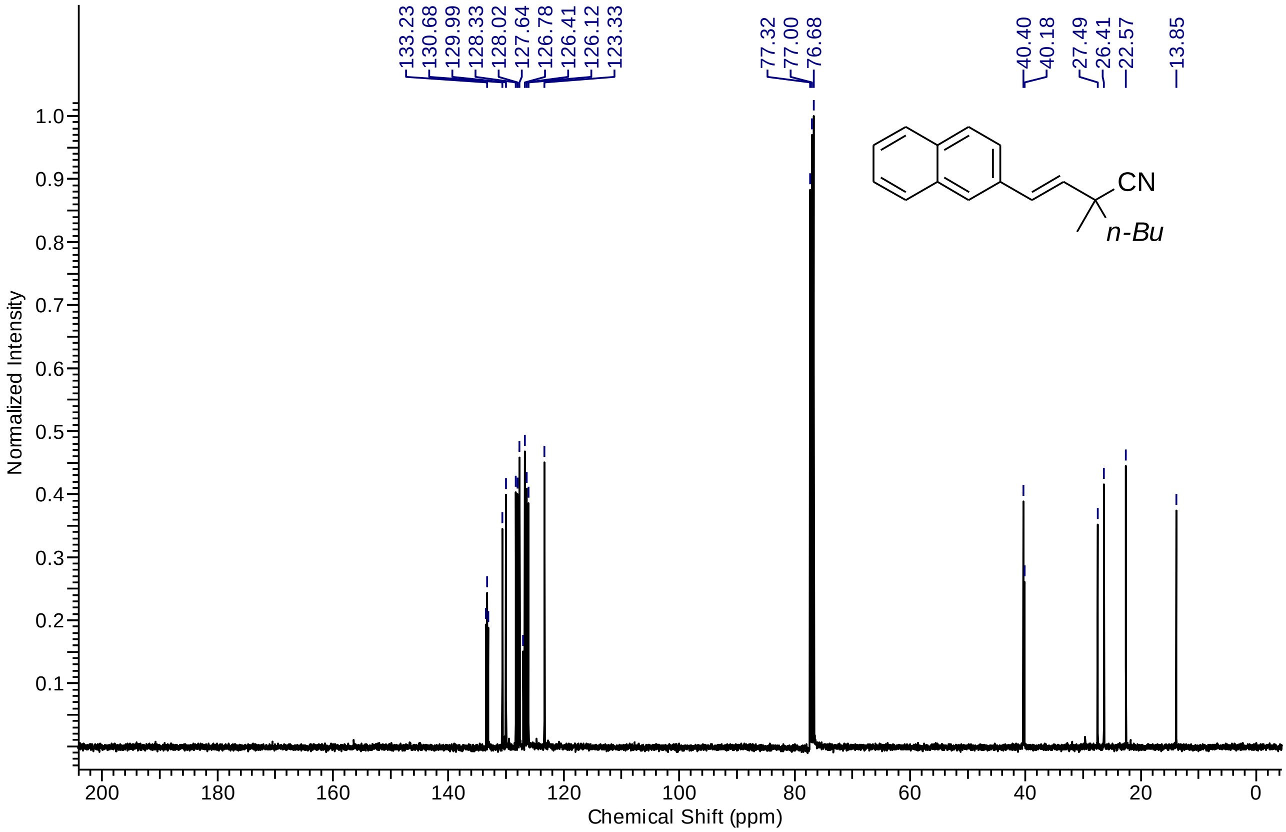

^{13}C NMR spectrum of the compound **3y**in $CDCl_3$, 100 MHz

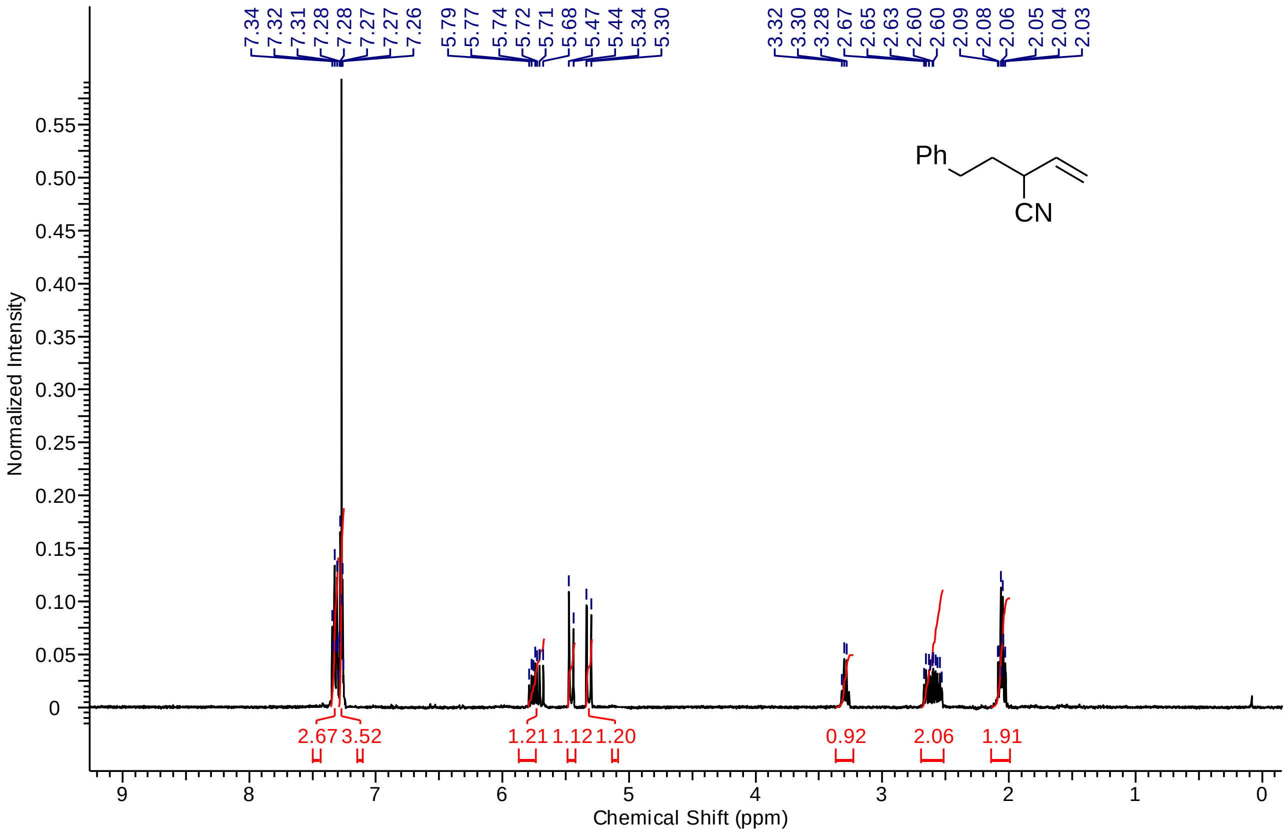

^{1}H NMR spectrum of the compound **5a** in $CDCl_3$, 400 MHz

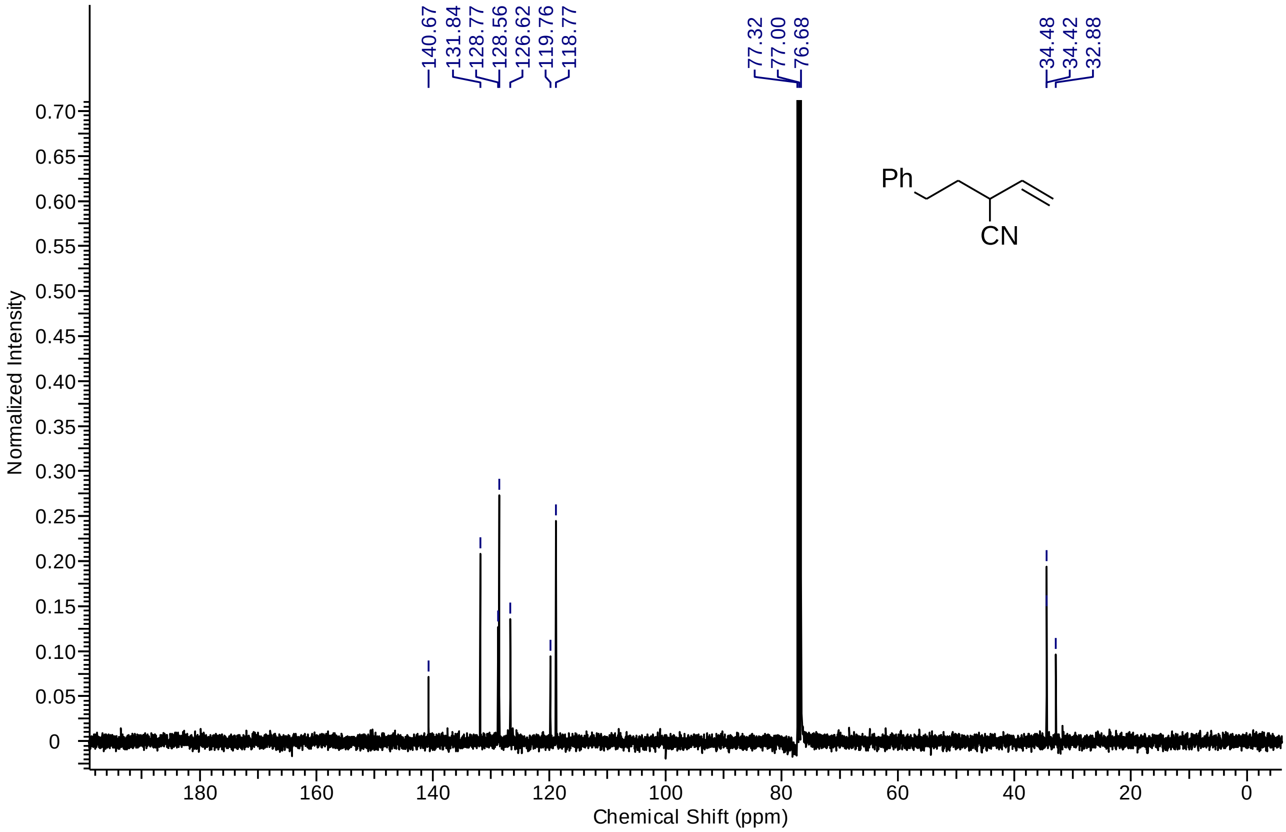

^{13}C NMR spectrum of the compound **5a** in $CDCl_3$, 100 MHz

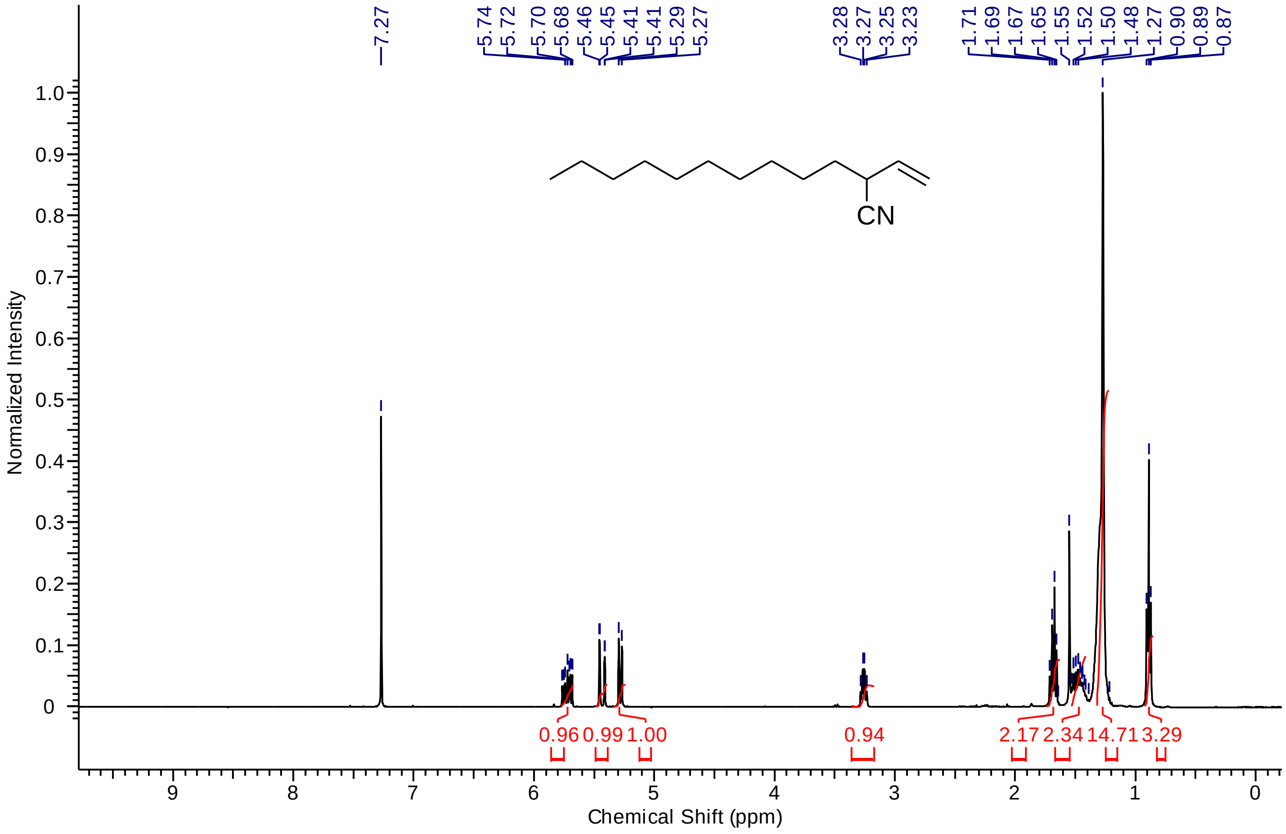

^{1}H NMR spectrum of the compound **5b** in $CDCl_3$, 400 MHz

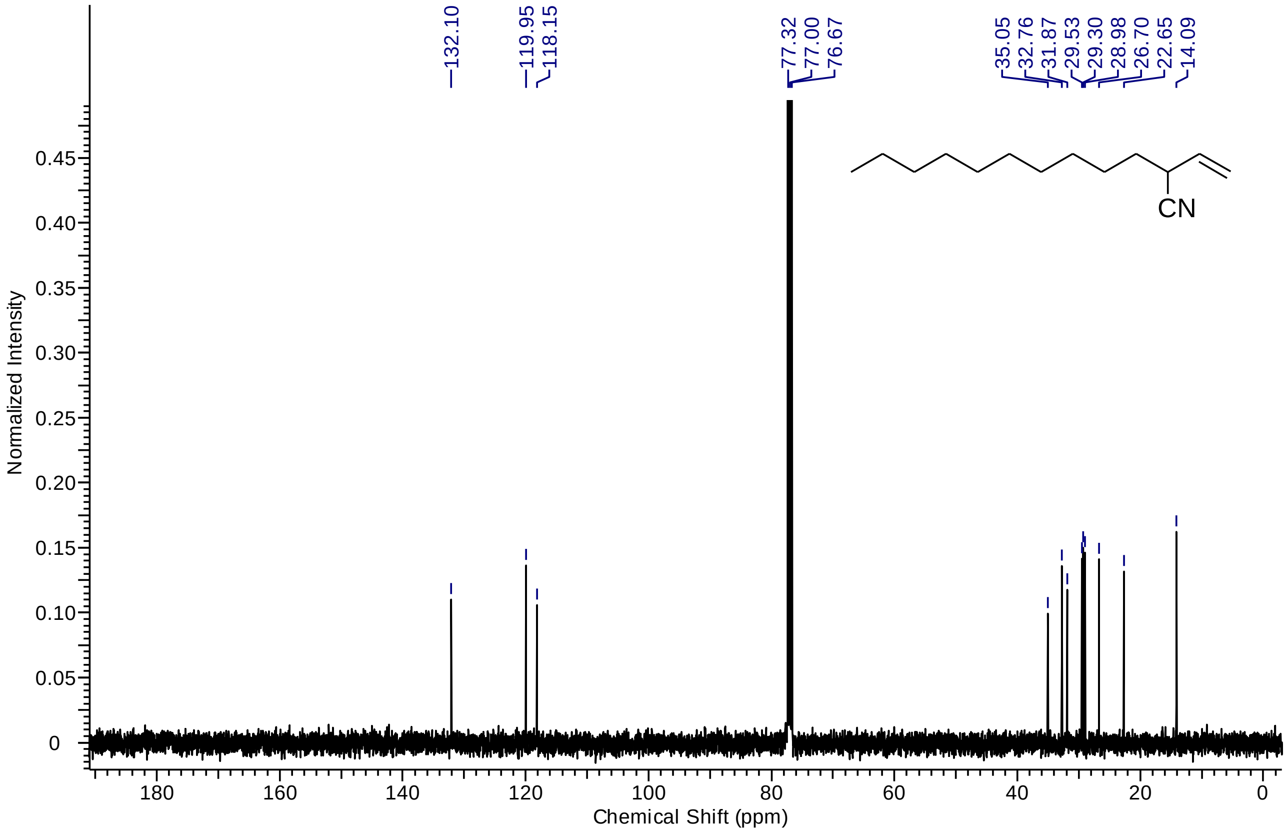

^{13}C NMR spectrum of the compound **5b** in $CDCl_3$, 100 MHz

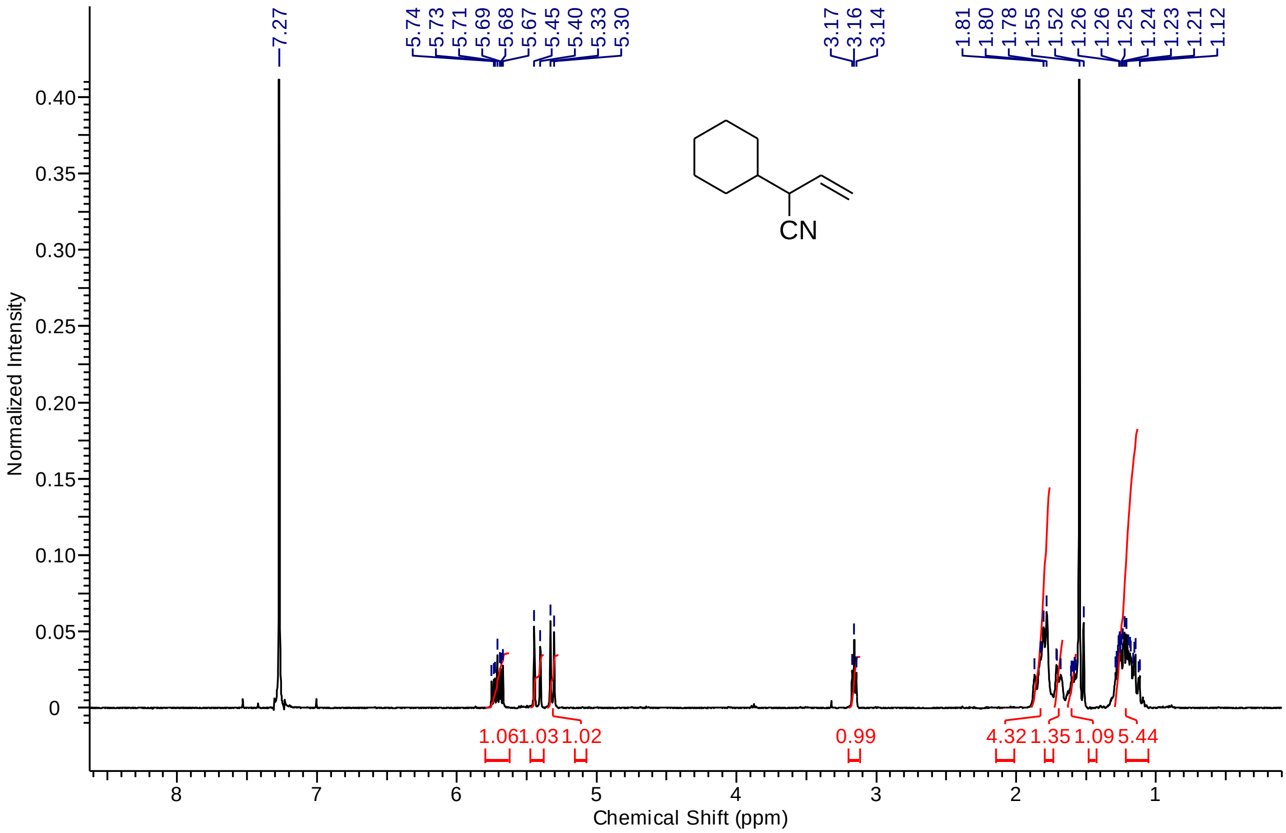

^{1}H NMR spectrum of the compound **5c** in $CDCl_3$, 400 MHz

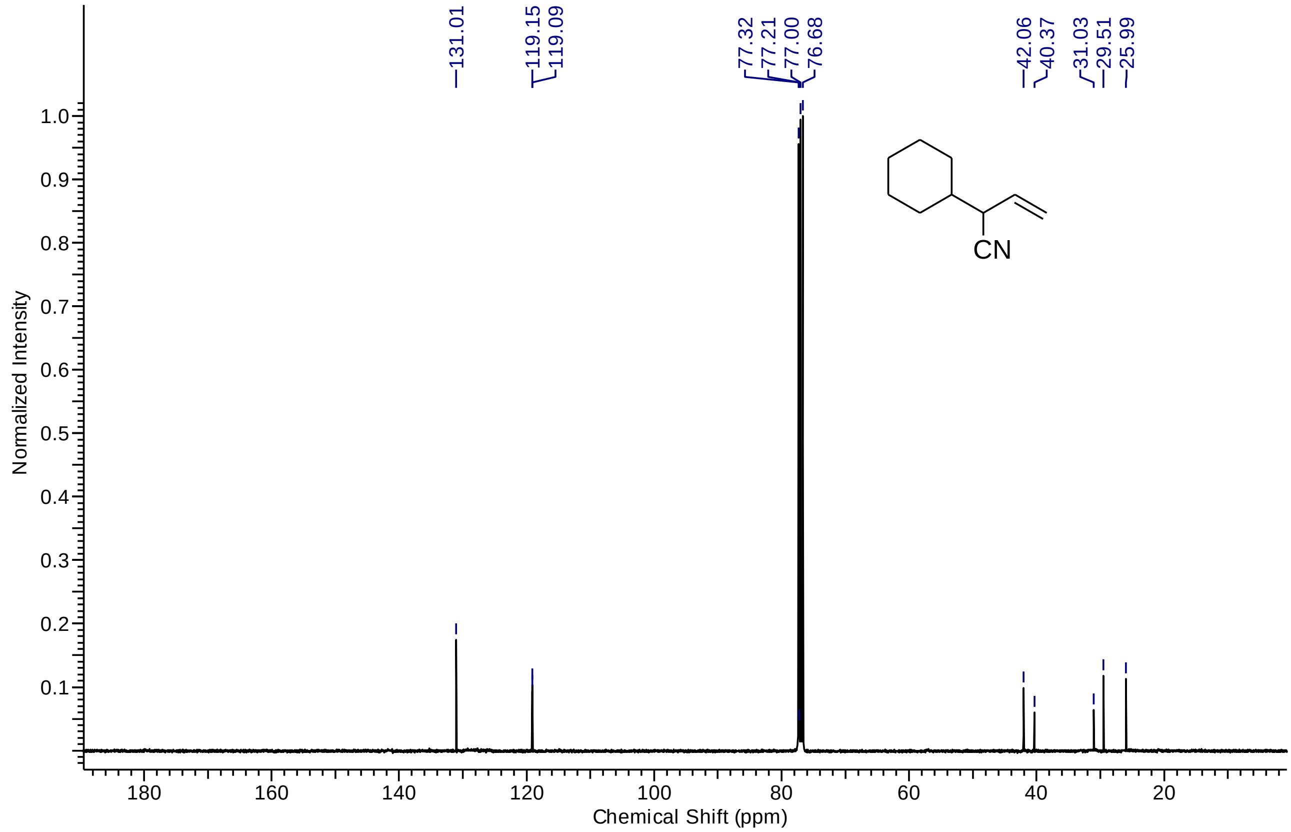

^{13}C NMR spectrum of the compound **5c** in $CDCl_3$, 100 MHz

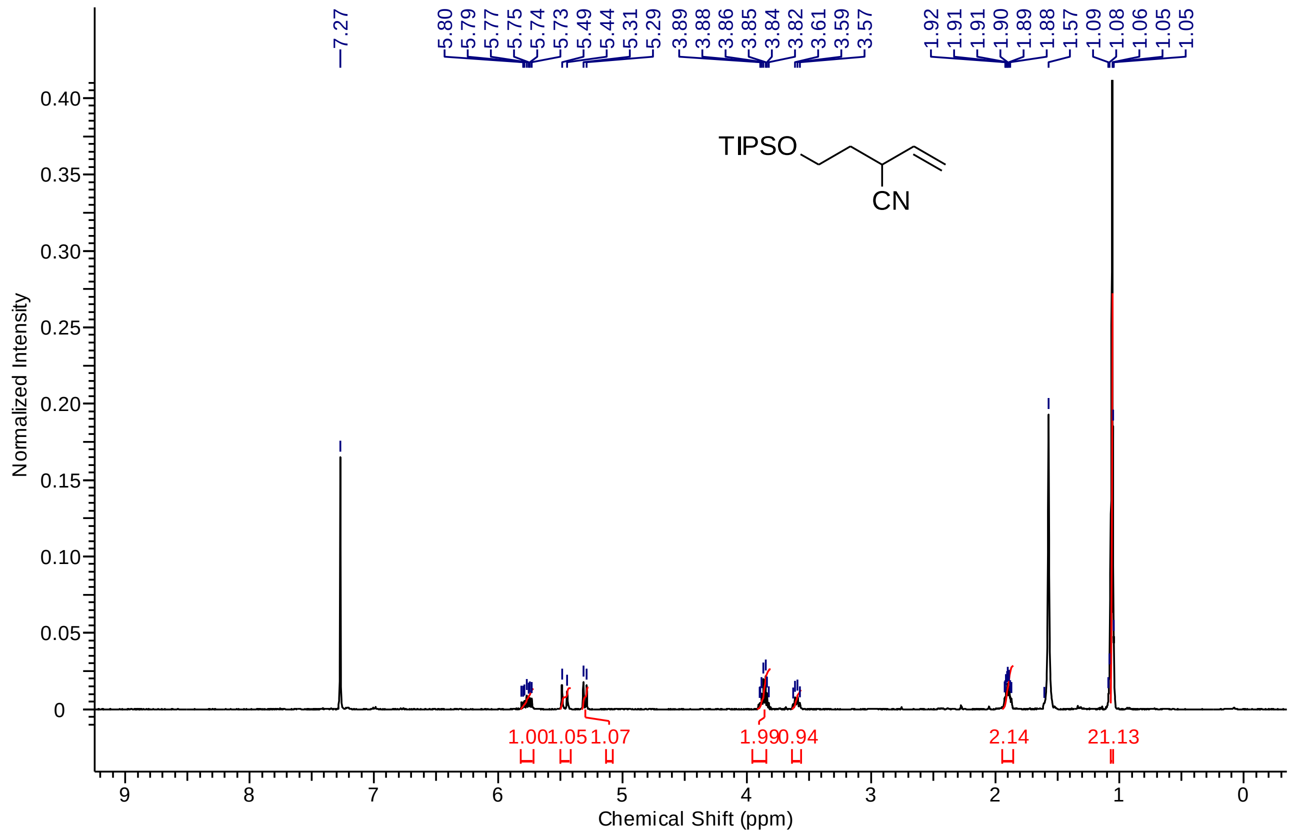

^{1}H NMR spectrum of the compound **5d** in $CDCl_3$, 400 MHz

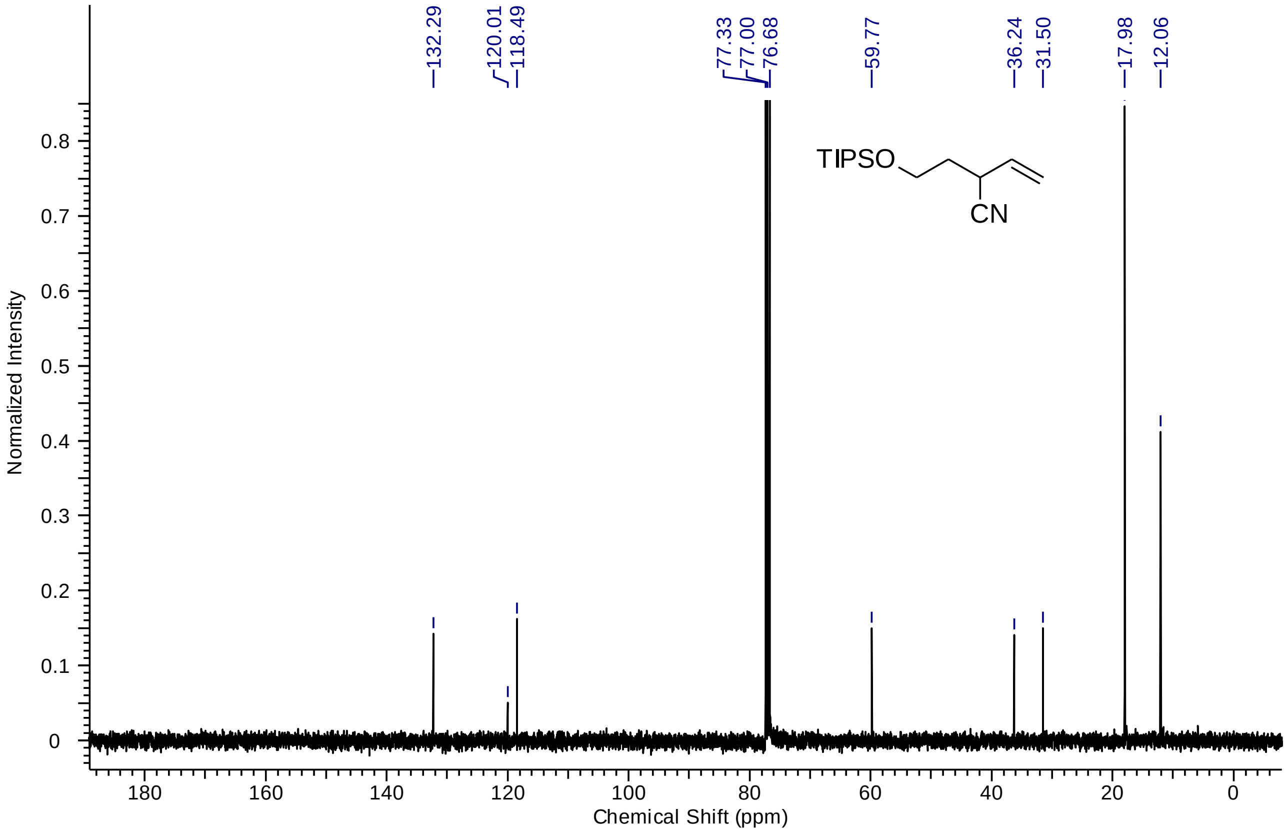

^{13}C NMR spectrum of the compound **5d** in $CDCl_3$, 100 MHz

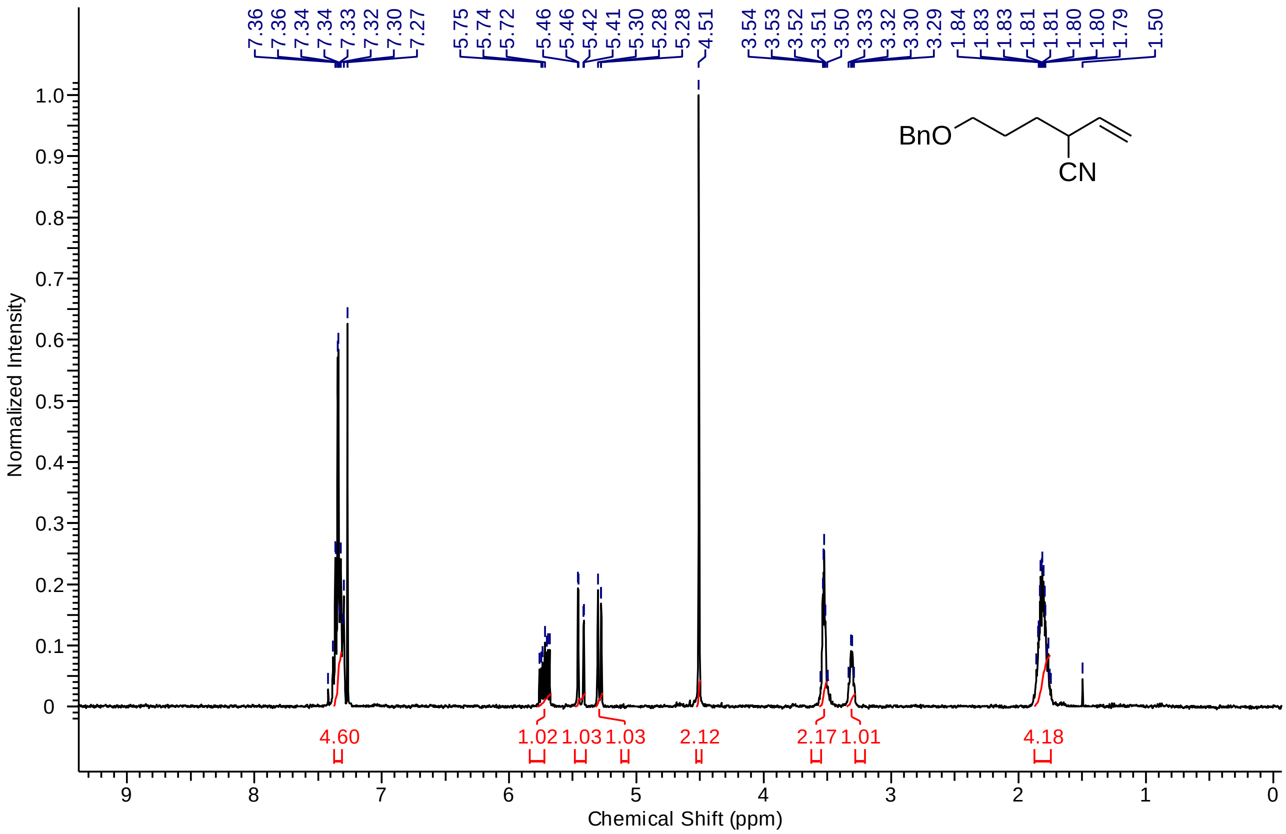

^{1}H NMR spectrum of the compound **5e** in $CDCl_3$, 400 MHz

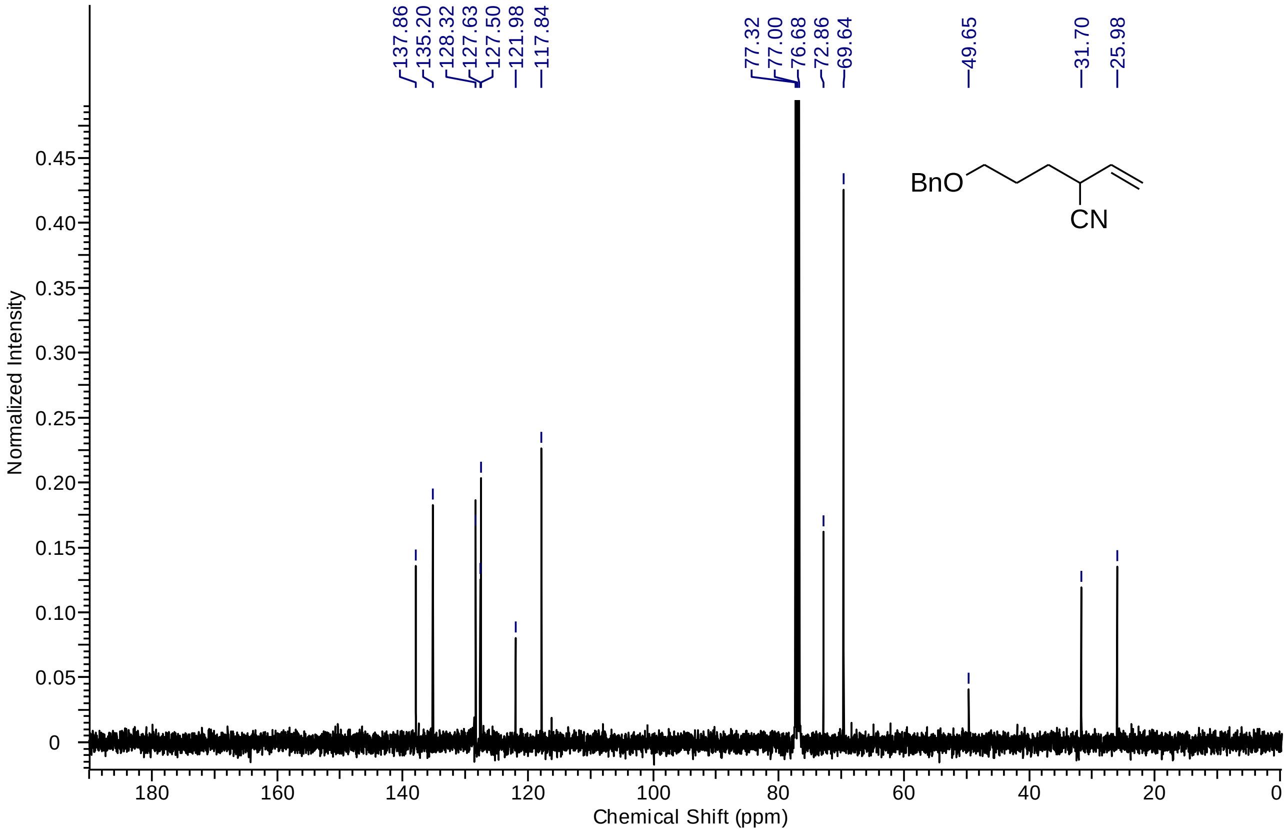

^{13}C NMR spectrum of the compound **5e** in $CDCl_3$, 100 MHz

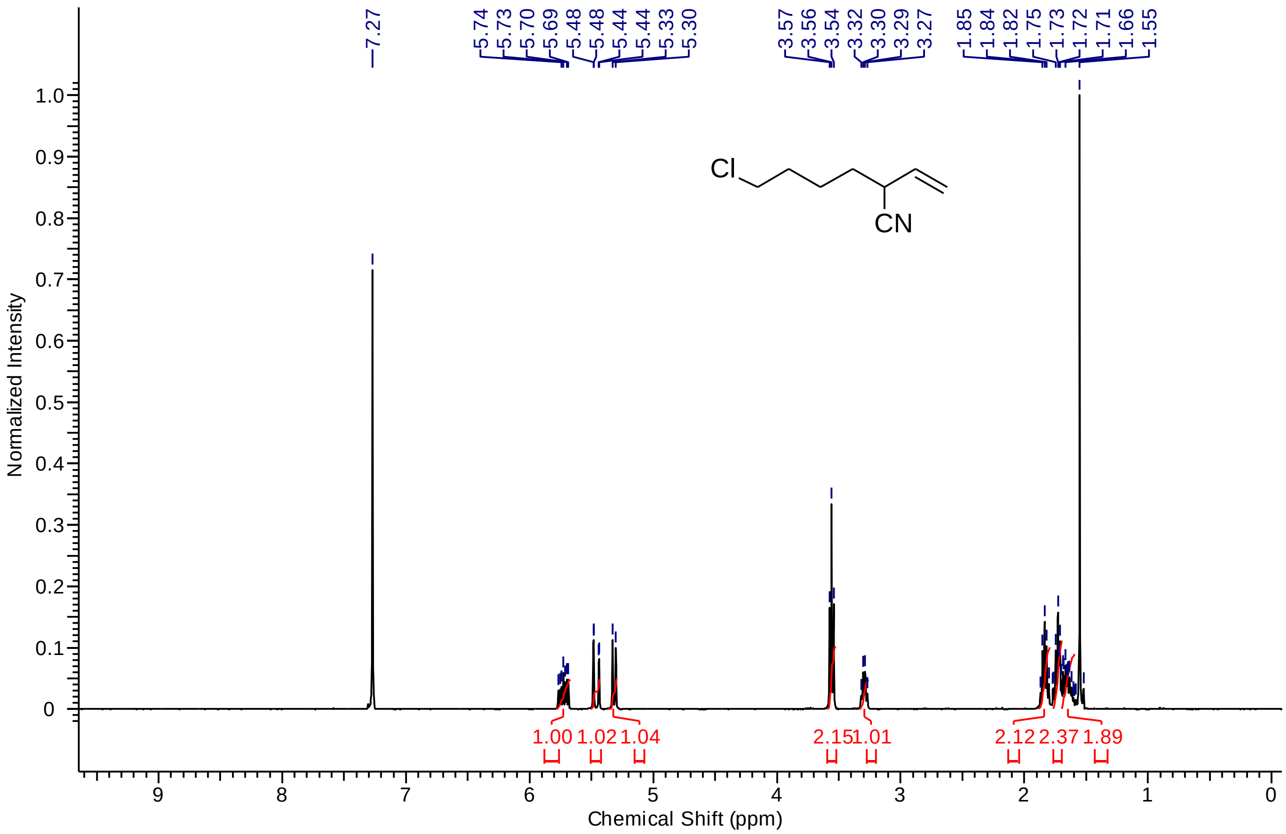

^{1}H NMR spectrum of the compound **5f** in $CDCl_3$, 400 MHz

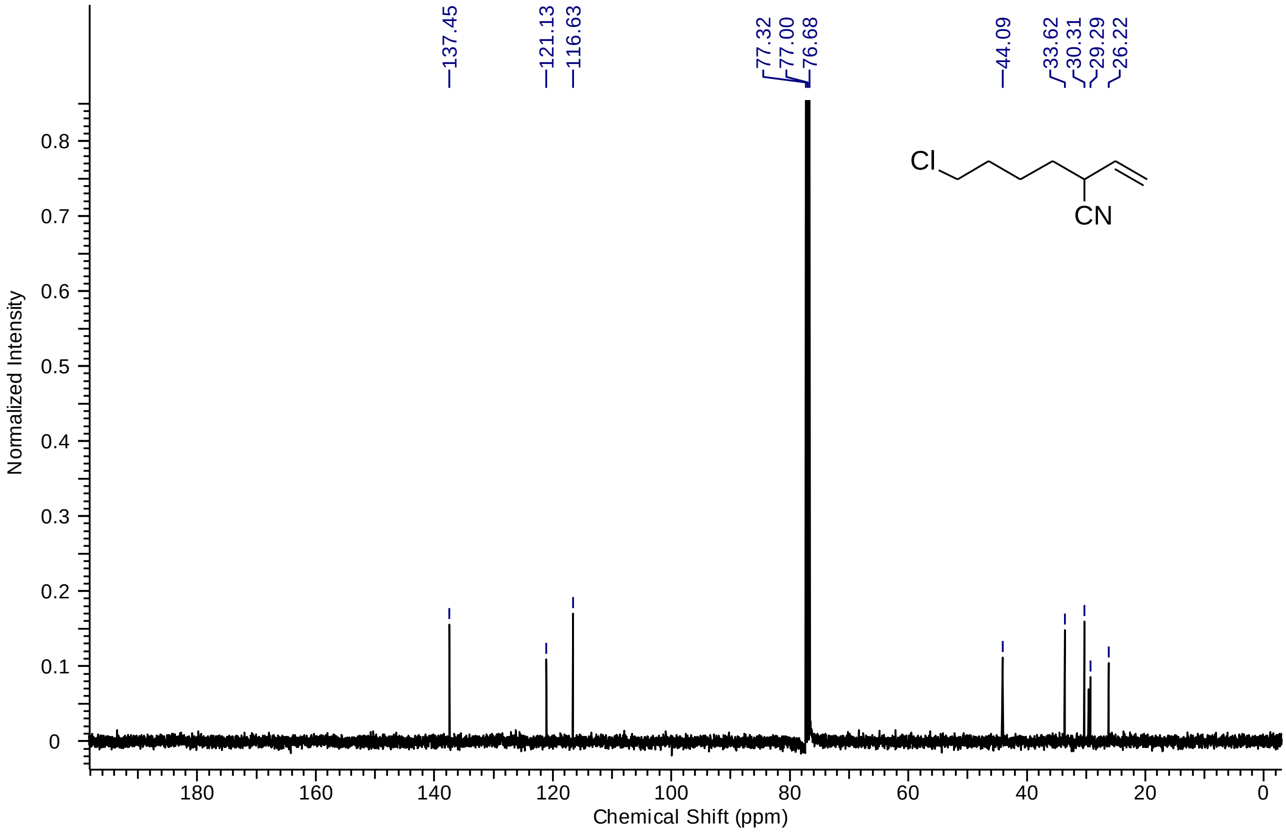

^{13}C NMR spectrum of the compound **5f** in $CDCl_3$, 100 MHz

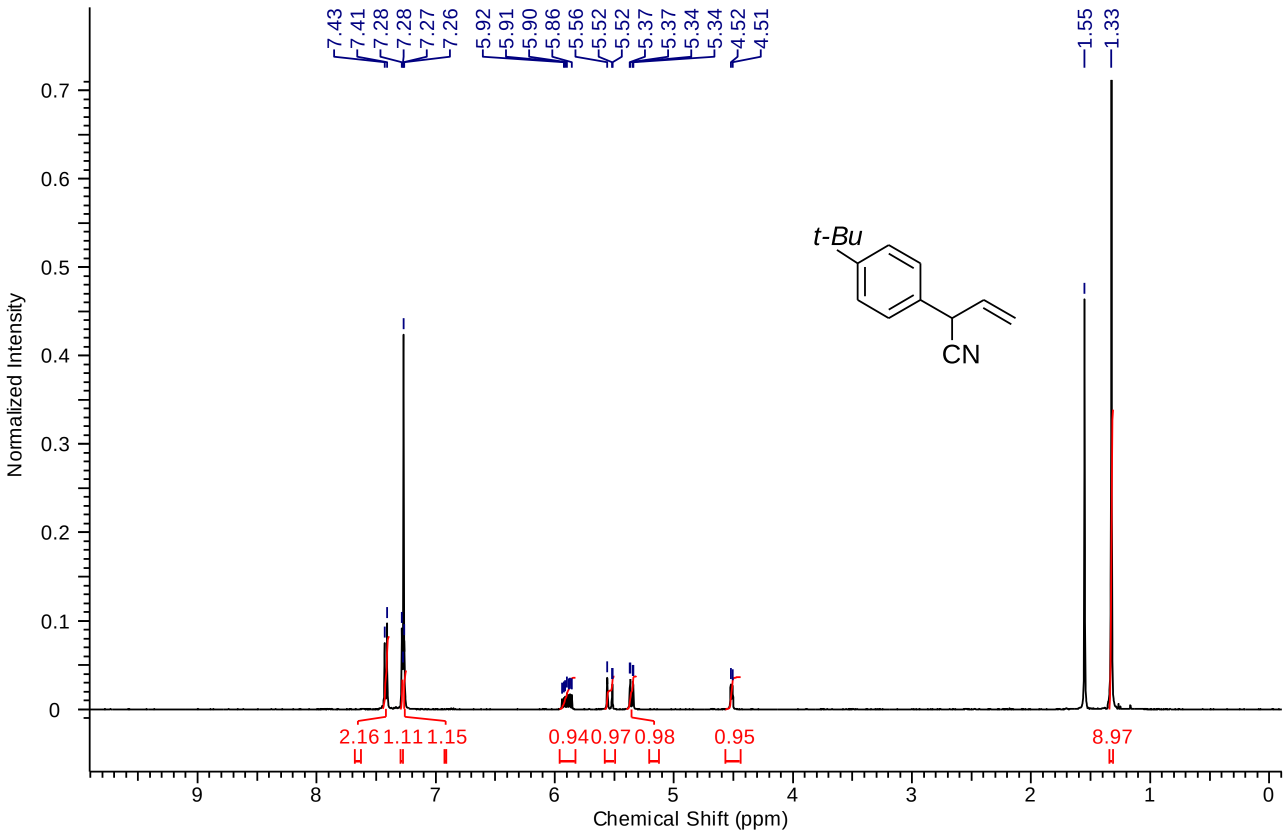

^{1}H NMR spectrum of the compound **5g** in $CDCl_3$, 400 MHz

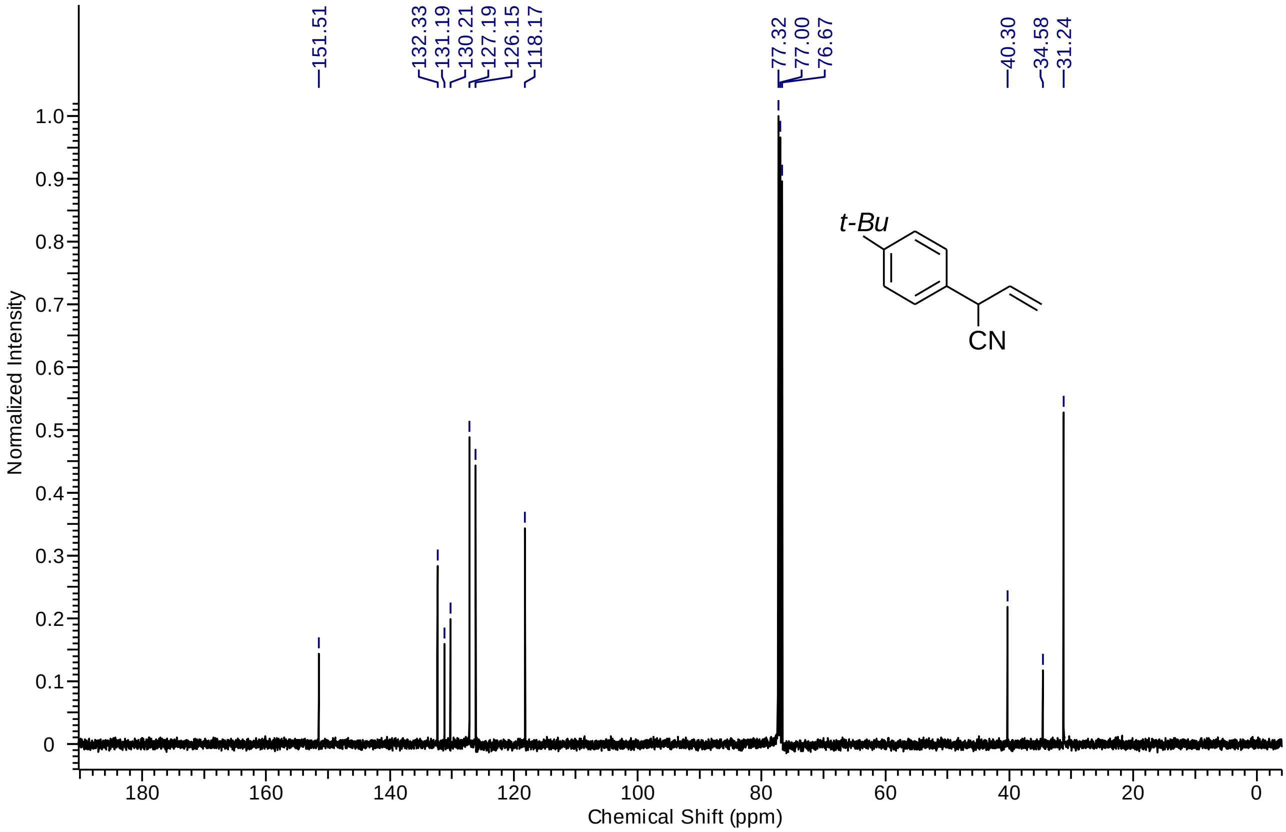

^{13}C NMR spectrum of the compound **5g** in $CDCl_3$, 100 MHz

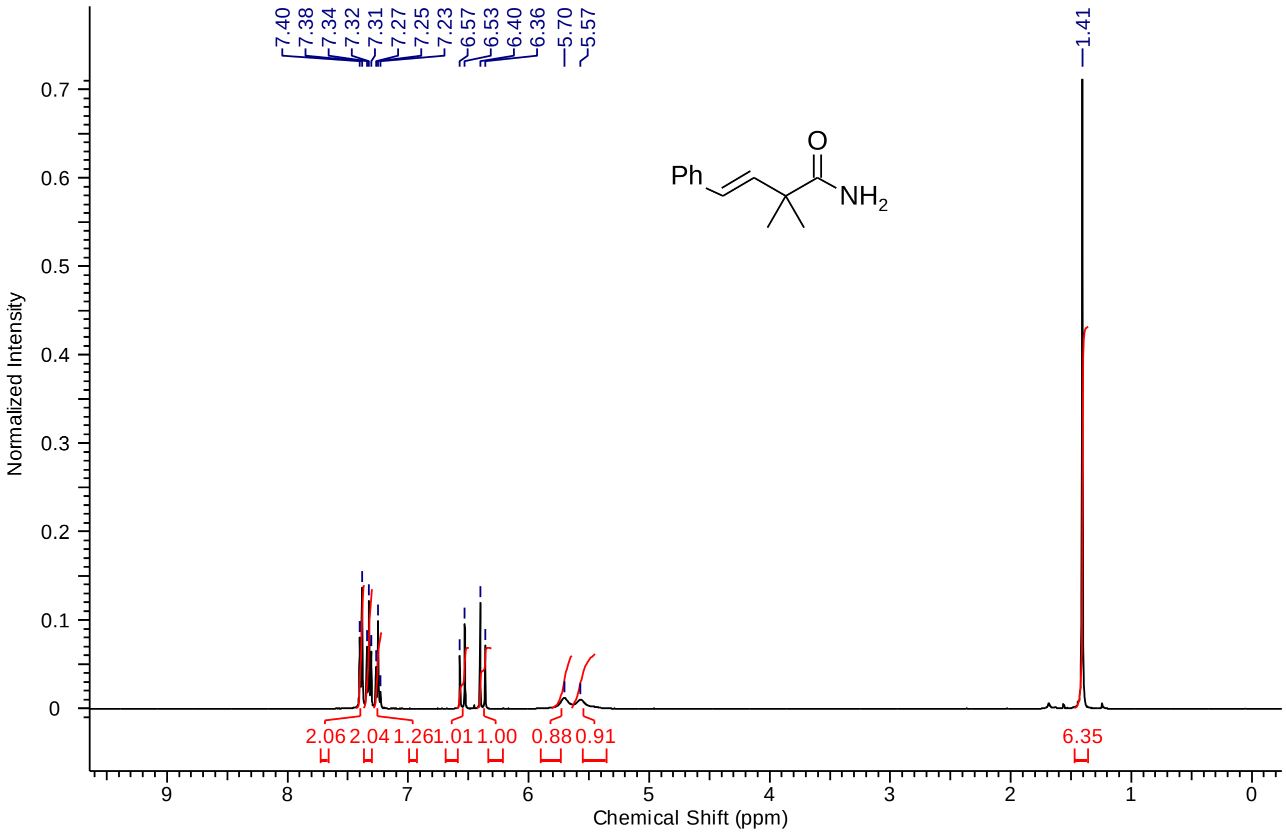

^{1}H NMR spectrum of the compound **6** in $CDCl_3$, 400 MHz

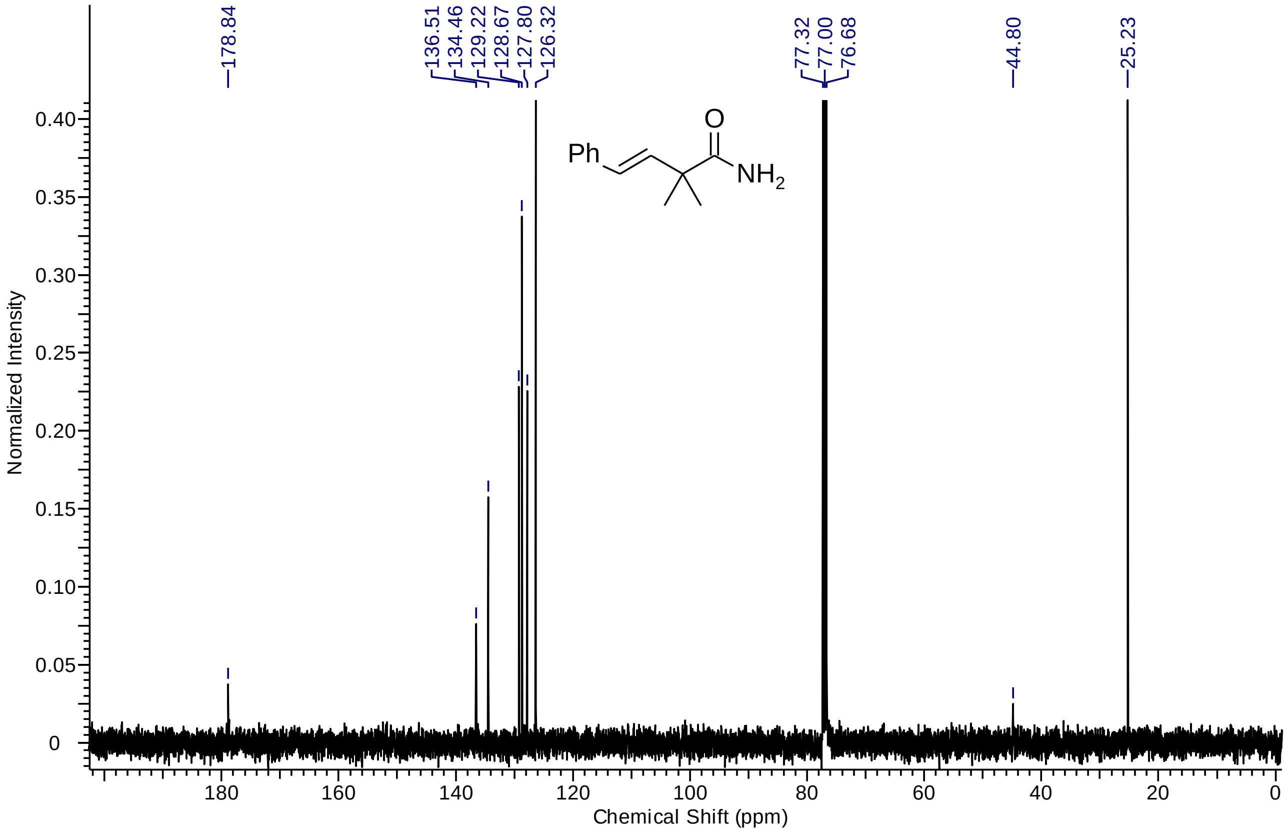

^{13}C NMR spectrum of the compound **6** in $CDCl_3$, 100 MHz

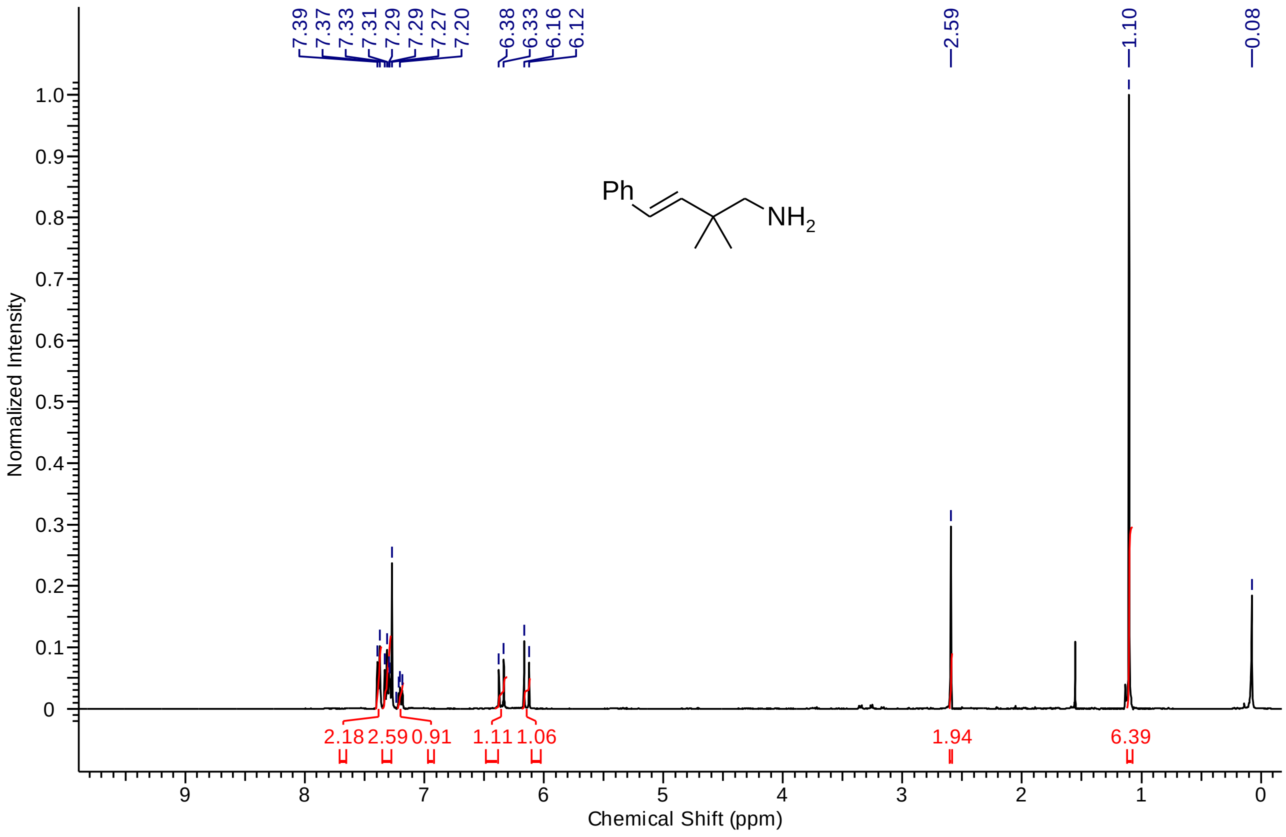

^{1}H NMR spectrum of the compound **7** in $CDCl_3$, 400 MHz

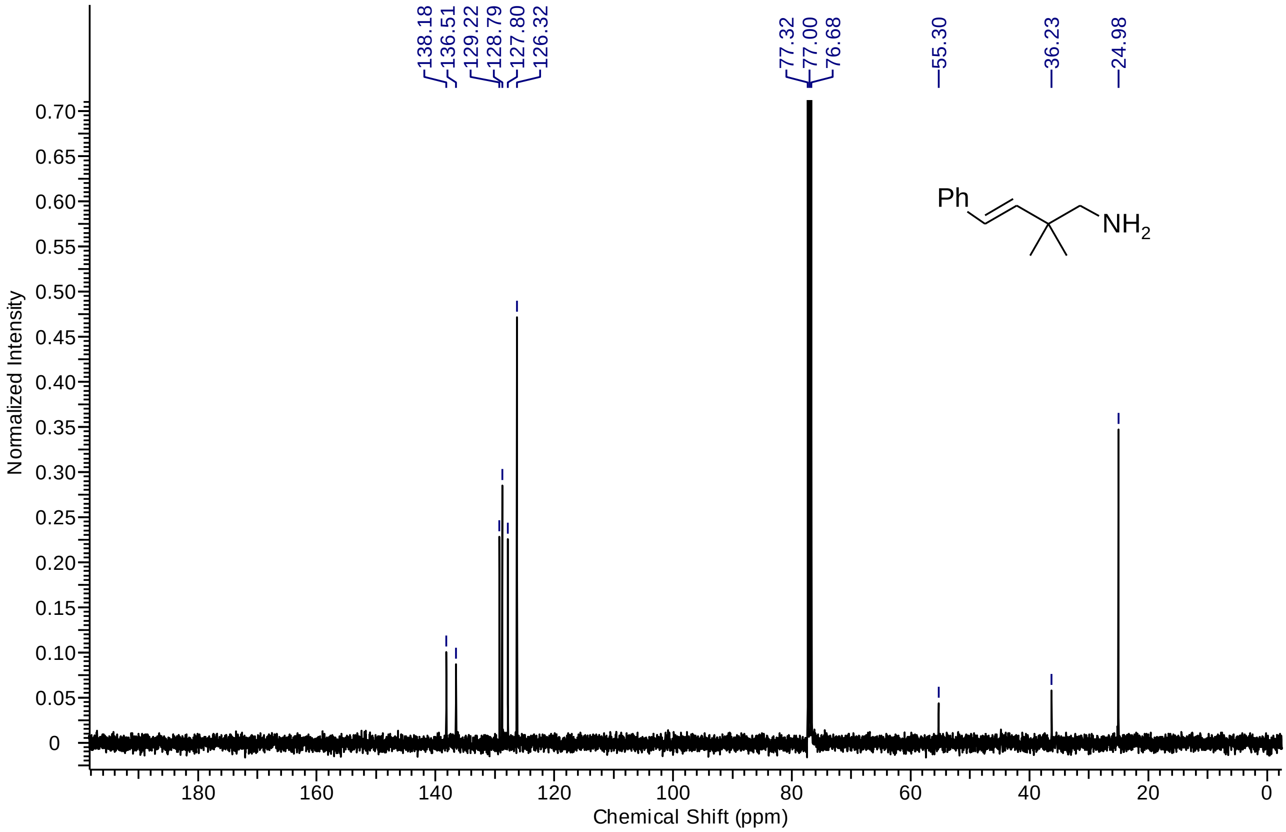

^{13}C NMR spectrum of the compound **7** in $CDCl_3$, 100 MHz

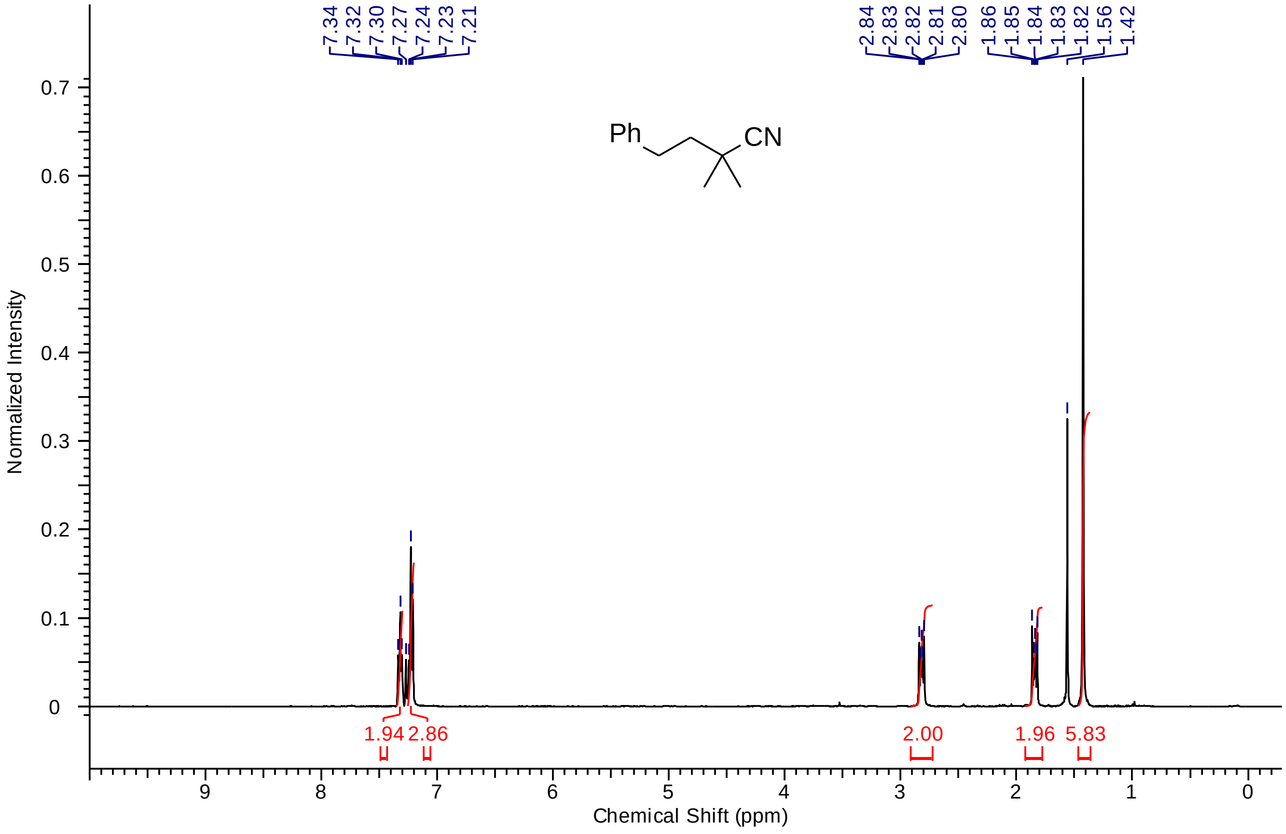

^{1}H NMR spectrum of the compound **8** in $CDCl_3$, 400 MHz

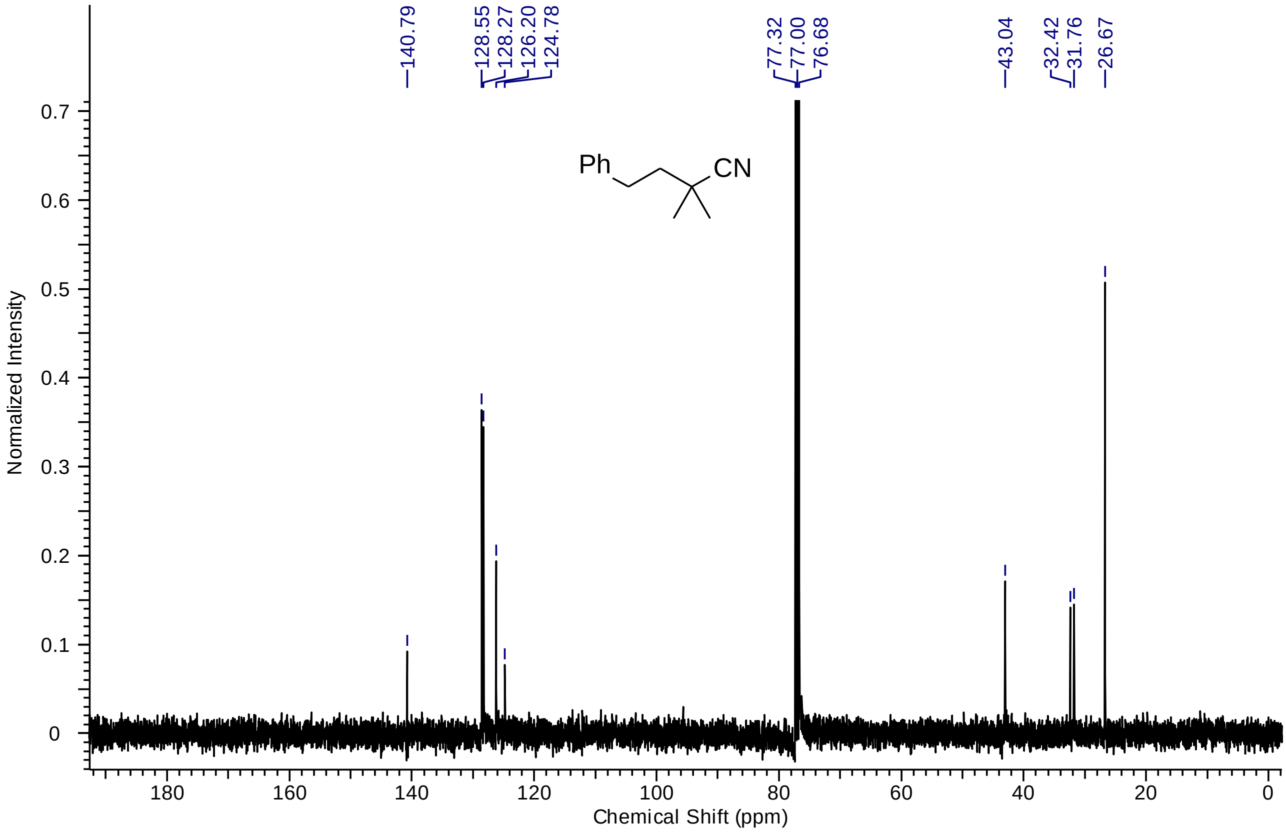

^{13}C NMR spectrum of the compound **8** in $CDCl_3$, 100 MHz

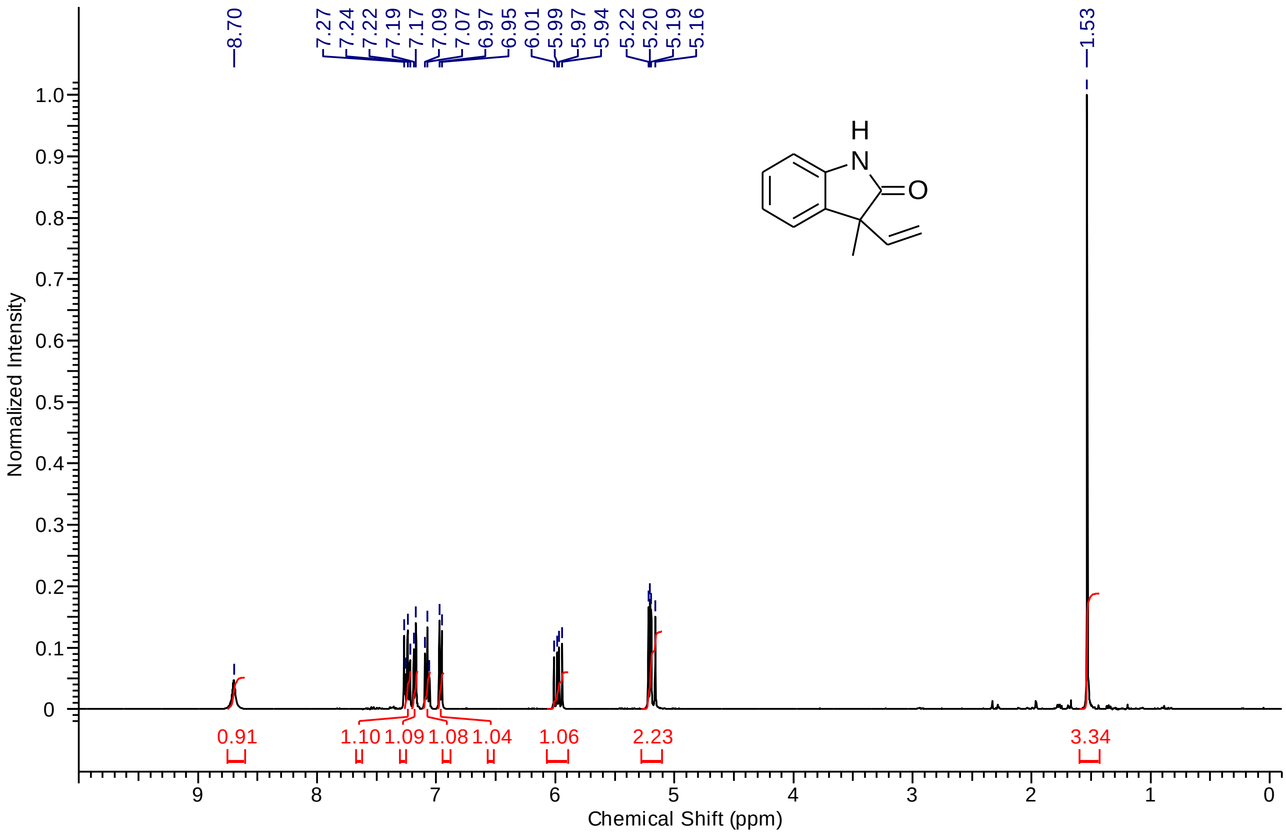

^{1}H NMR spectrum of the compound **9** in $CDCl_3$, 400 MHz

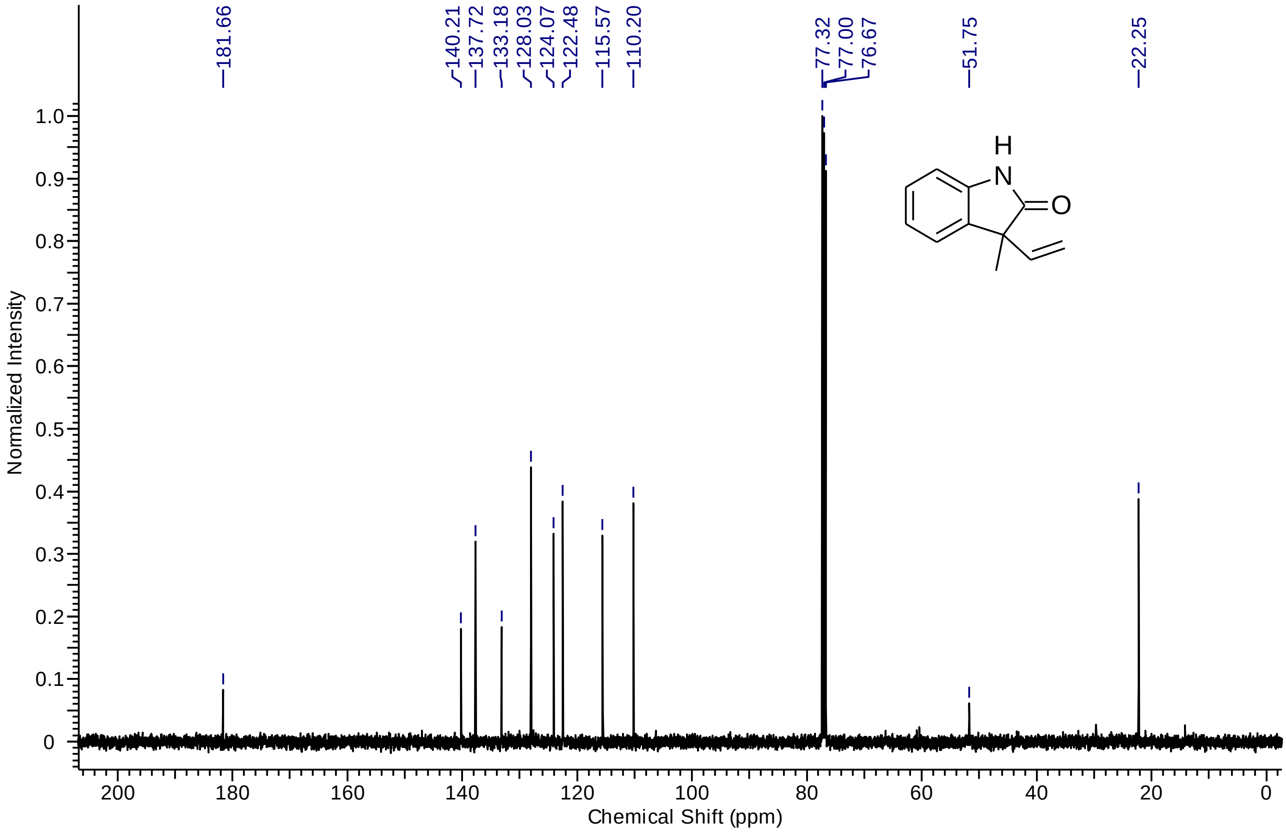

^{13}C NMR spectrum of the compound **9** in $CDCl_3$, 100 MHz
